# Correlation between the Molecular Properties of Semiconducting Polymers of Intrinsic Microporosity and Their Photocatalytic Hydrogen Production

**DOI:** 10.1021/jacs.4c08549

**Published:** 2024-10-30

**Authors:** Benjamin J. Willner, Catherine M. Aitchison, Filip Podjaski, Wanpeng Lu, Junfu Tian, James R. Durrant, Iain McCulloch

**Affiliations:** a Department of Chemistry, Chemistry Research Laboratory, Oxford University, 12 Mansfield Road, Oxford OX1 3TA, U.K.; b Department of Chemistry and Centre for Processable Electronics, Imperial College London, 80 Wood Lane, London W12 0BZ, U.K.; c Andlinger Center for Energy and the Environment and Department of Electrical and Computer Engineering, Princeton University, Princeton, New Jersey 08544, United States

## Abstract

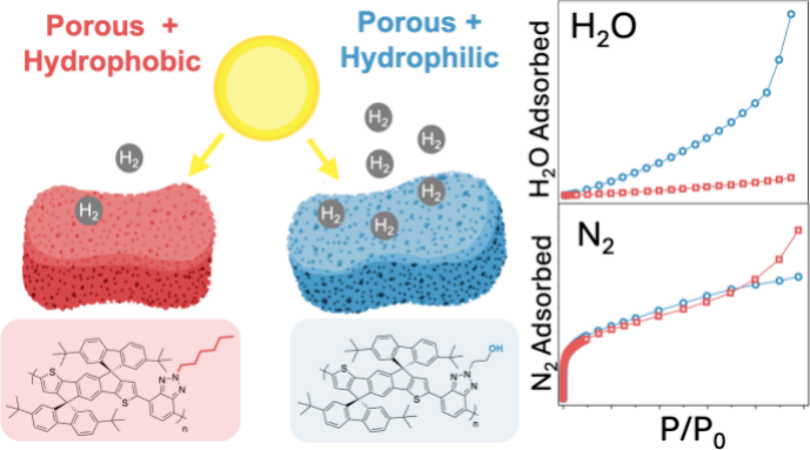

Increasing the interface area between organic semiconductor photocatalysts and electrolyte by fabricating nanoparticles has proven to be an effective strategy to increase photocatalytic hydrogen production activity. However, it remains unclear if increasing the *internal* interface by the introduction of porosity has as clear benefits for activity. To better inform future photocatalyst design, a series of polymers of intrinsic microporosity (PIMs) with the same conjugated backbone were synthesized as a platform to independently modulate the variables of porosity and relative hydrophilicity through the use of hydrophilic alcohol moieties protected by silyl ether protecting groups. When tested in the presence of ascorbic acid and photodeposited Pt, a strong correlation between the *wettable* porosity and photocatalytic activity was found, with the more wettable analogue of two polymers of almost the same surface area delivering 7.3 times greater activity, while controlling for other variables. Transient absorption spectroscopic (TAS) investigation showed efficient intrinsic charge generation within 10 ps in two of the porous polymers, even without the presence of ascorbic acid or Pt. Detectable hole polarons were found to be immediately extracted by added ascorbic acid, suggesting the generation of reactive charges at regions readily accessible to electrolyte in the porous structures. This study directs organic semiconductor photocatalysts design toward more hydrophilic functionality for addressing exciton and charge recombination bottlenecks and clearly demonstrates the advantages of wettable porosity as a design principle.

## Introduction

To decarbonize the global economy and meet CO_2_ emission reduction targets, renewable H_2_ will play an increasingly important role and must be produced efficiently at scale if its potential is to be realized.^1−3^ Using conventional photovoltaic and water electrolysis technology, it is possible to produce renewable H_2_ by using solar energy. However, this two-step process of generating electricity to drive electrolysis is more complex, costly and has lower theoretical efficiency limits compared to direct processes.^4,5^ Research into direct photocatalytic production of H_2_ from sunlight and water has seen growing interest because of the potential for lower costs in the long-term.^5−10^ Inorganic semiconductors typically have wide bandgaps,^11^ meaning much of the lower energy solar spectrum cannot be utilized.^12^ Organic semiconductors present advantages as photocatalysts compared to their inorganic counterparts due to their highly tunable energy levels toward the solar spectrum,^13,14^ high extinction coefficients,^15^ and their potential for low-cost solution processability which enables greater control of morphology.^16^

Elucidating the material properties that give the highest photocatalytic activity for organic semiconductors is challenging because H_2_ evolution rates depend on the coalescence of numerous interrelated properties, and varying each property in isolation is difficult.^17,18^ In photocatalysts, the energies of the frontier molecular orbitals (FMOs) must provide sufficient electrochemical potential to drive proton reduction and its complementary oxidation half-reaction, while simultaneously maintaining an optical bandgap tailored to the solar spectrum.^9,19,20^ However, consideration of the FMO energies alone is not sufficient to design active organic semiconductor photocatalysts. Other important factors include designing suitable interfaces to promote efficient exciton separation,^21,22^ the ability of excitons and polarons to diffuse through the semiconductor to interfaces,^23^ the nature and distribution of cocatalysts,^24−26^ as well as photocatalyst particle size,^27−29^ hydrophilicity^30−32^ and corresponding dispersibility in the aqueous testing medium.^20^ These properties are, in turn, determined by the molecular structure of the photocatalyst and how it is processed.

For heterogeneous catalysis, the interface area between the catalyst and electrolyte has a strong influence on activity because it determines the number of potential active sites.^33,34^ Photocatalysis using organic semiconductors is complicated by the need to separate tightly bound photogenerated Frenkel-type excitons.^35,36^ This can be achieved by blending it with another semiconductor that exhibits offset FMO energy levels to generate a type-II heterojunction^21,37^ or through the photocatalyst’s interface with redox species in the electrolyte such as charge mediators in Z-schemes^38^ or sacrificial electron donors (ascorbic acid or triethylamine). Such species can reductively separate excitons,^22^ liberating election polarons to drive proton reduction on the semiconductor. However, it should be noted that sacrificial electron donors are used as an experimental tool to decouple proton reduction and water oxidation half-reactions. This helps elucidate structure–property relationships on the pathway to overall water splitting, including through the use of Z-schemes which can utilize low-energy photons.

For processable organic semiconductor photocatalysts, increasing *external* interfacial area with electrolyte by reducing particle size via miniemulsion^21,28,37^ or nanoprecipitation^27,39−41^ methods has proven an effective strategy to increase photocatalytic activity. This is because excitons can typically only diffuse 5 to 15 nm through the semiconductor before they decay.^35,42−44^ However, even for such nanoparticles, if excitons are photogenerated in the bulk of the nanoparticle at distances from an interface greater than the semiconductor’s exciton diffusion length (and intrinsic charge generation pathways are absent), they are typically unable to separate into catalytically active charges. Such internal photoexcitations are usually counterproductive because photons are absorbed whose energy cannot be productively utilized, at the expense of photoexcitation in regions within reach of interfaces.^17^

Introduction of porosity can generate large internal interfacial areas in photocatalysts. This can be achieved by the synthesis of network-conjugated polymers, which can covalently “lock-in” void space by interconnection of cross-linking monomers. Such network polymers in the form of covalent organic frameworks or conjugated microporous polymers can achieve considerable surface areas well over 1000 m^2^ g^–1^.^45,46^ While these high interfacial areas would be expected to be favorable for photocatalysis due to the reduced distance of photoexcitation from the electrolyte interface, microporous photocatalysts have often failed to realize this expected improvement in activity, calling into question the merit of porosity as a design principle.^14,45,47−50^ One problem is that in order to generate porosity in cross-linked polymers, branched monomers are required. These units frequently involve meta linkages,^48,49,51^ which restrict conjugation, limiting both exciton and polaron transport as well as hindering absorption in the solar spectral range.^47,52,53^ Alternative ortho linkages are sterically demanding,^48^ which induce dihedral twisting^52^ and limit the extent of polymerization,^51,54^ further attenuating conjugation and impairing exciton and polaron transport.^55−57^

Another difficulty with the rationalization of porosity is that apparent surface areas are derived from N_2_ adsorption isotherm measurements using Brunauer–Emmett–Teller (BET) surface area calculations.^58^ This technique gives little insight into the access of the electrolyte to the pores. Reports of a poor correlation between porosity and photocatalytic activity have predominantly been for hydrophobic materials, such as conjugated microporous polymers composed of nonpolar monomers.^14,47^ By contrast, in hydrophilic structures containing heteroatoms^59^ (in particular highly polar dibenzo[b,d]thiophene sulfone^51,60−62^ units), introducing porosity has been shown to be conducive to increased activity. This is thought to be primarily because only hydrophilic pore surfaces allow aqueous electrolyte entry^51^ and thus increase the active semiconductor-electrolyte interface area. Nonetheless, hydrophilicity in such porous polymers is often achieved through polymer backbone modification, which simultaneously alters other properties, such as light absorption and charge separation. This entanglement of simultaneously changing variables means that unambiguous investigation of the intertwined effects of porosity and relative hydrophilicity is currently lacking.

In this work, polymers of intrinsic microporosity (PIMs) are used to investigate the parameters of the porosity and hydrophilicity for conjugated polymer photocatalysts. PIMs are a class of polymers whose porosity arises from their rigid and contorted backbones. This along with low conformational freedom hinders packing in the solid state and leads to the presence of voids, which may be interconnected. In this manner, incorporation of “sterically demanding” monomers can generate linear conjugated polymers^63−67^ with high degrees of microporosity (pores <2 nm) and mesoporosity (pores 2 to 50 nm).^68^ In PIMs, microporosity is achieved without the need for cross-linking and so branched conjugation blocking units can be avoided. Perhaps even more significantly, PIMs’ linear structures can enable solubility in organic solvents, which allows better processing, characterization of optoelectronic properties and batch-to-batch variables such as molecular weight and purity.

**Figure 1:**
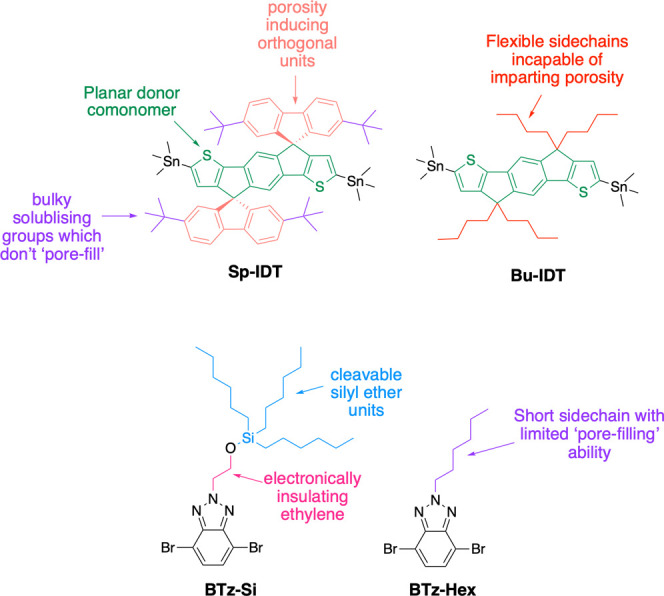
Design of monomers combined to form the polymer series.

Here, PIMs and nonporous polymers possessing the same conjugated backbone were synthesized to investigate the effects of porosity and relative hydrophilicity. This was achieved by the design of two indacenodithiophene (IDT)-based monomers. Spiro groups involve two orthogonal rings linked by a shared sp^3^-hybridized atom.^69^ Their incorporation into conjugated polymers have been shown to impart intrinsic microporosity due to inhibiting packing in the solid state.^60^ Porosity-inducing *tert*-butyl spirofluorene units were incorporated onto the methylene bridge units on either side of the IDT resulting in the monomer Sp-IDT (Figure 1). The additional placement of *tert*-butyl groups on the orthogonal spirofluorene units increases their steric bulk to further assist void formation and can aid solubility.^70−73^ In order to compare Sp-IDT intrinsically microporous polymers to polymers with the same conjugated backbones lacking the ability to induce intrinsic microporosity, Bu-IDT (Figure 1) with flexible linear aliphatic side-chains was designed to produce nonporous polymers.

Next, to modulate the relative hydrophilicity of the polymers, a 1,2,3-benzotriazole comonomer, BTz-Si (Figure 1) was functionalized with a silyl ether-protected alcohol. This silyl ether-protecting group was able to be removed post-polymerization to reveal hydrophilic alcohol functional groups on the polymers, acting as a “chemical switch” to increase relative hydrophilicity. Ethylene spacers between the silyl ether and conjugated unit were incorporated to electronically insulate the polymer backbones from side-chain modifications. This postpolymerization strategy thus ensured the protected and deprotected polymers maintained the same number of repeat units and polydispersity, as well as the same conjugated backbone better enabling a fair comparison. In general, PIMs cannot possess excessive conformationally flexible side chains as these are able to fill in the voids generated by rigid and contorted backbones.^74^ We used silyl ether protecting groups with large conformationally flexible porosity-blocking side chains, which meant that as well as revealing hydrophilic alcohols for enhanced wettability, deprotection also acts as a switch to “turn on” porosity. To allow the generation of porous hydrophobic polymers, a 1,2,3-benzotriazole comonomer with hexyl side chains was designed, BTz-Hex (Figure 1). It had a limited ability to “fill in” the pores owing to its significantly less sterically bulky side-chain compared to trihexylsilyl ethyl ethers in BTz-Si. The series of polymers and their deprotected counterparts synthesized from combinations of these monomers were used to investigate the effects of relative hydrophilicity and porosity on their exciton and charge dynamics and their resultant photocatalytic activity.

## Results

### Polymers

**Figure 2:**
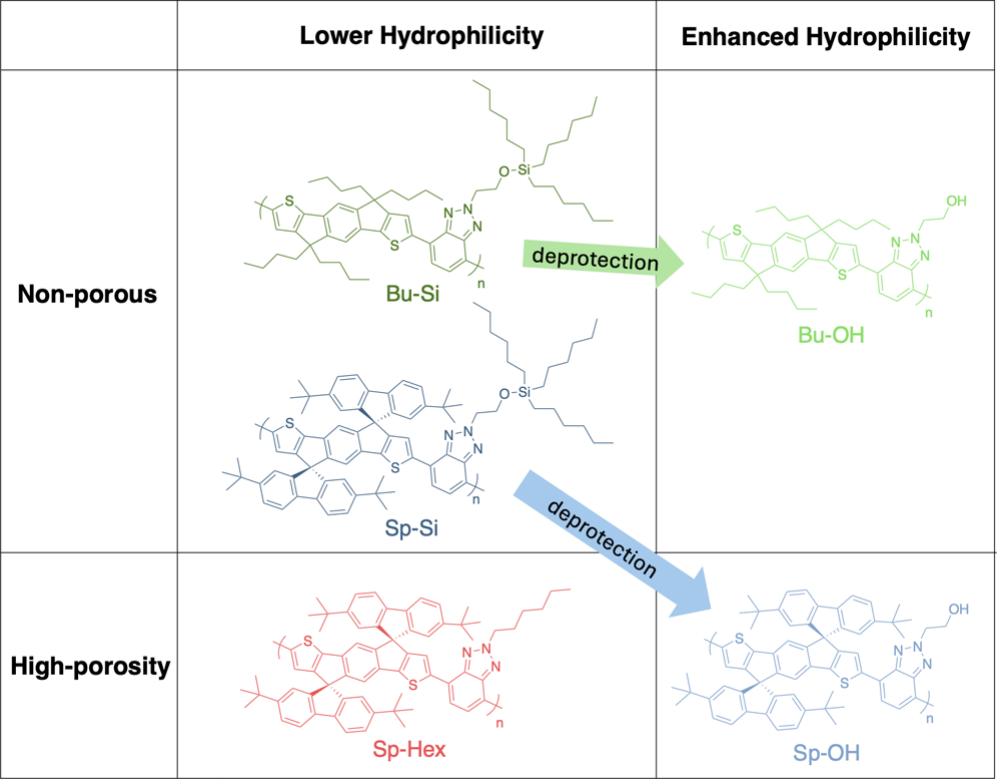
Polymers are categorized by porosity and relative hydrophilicity. **Bu–OH** and **Sp–OH** were derived by deprotecting portions of **Bu-Si** and **Sp-Si**, respectively.

Synthesis of the monomers Sp-IDT, Bu-IDT, BTz-Si, and BTz-Hex can be found in the SI. These were copolymerized under Stille conditions to form the three polymers **Sp-Si**, **Sp-Hex**, and **Bu-Si**. The silyl ether-protected alcohol polymers **Sp-Si** and **Bu-Si** were quantitively deprotected using neutralized tetrabutyl ammonium fluoride (TBAF) to form their alcohol analogues **Sp–OH** and **Bu–OH** (details and verification in the SI). The properties of the polymers and their deprotected counterparts are categorized in Figure 2.

### Optoelectronic Properties

The ionization potentials (IPs) for the solution processable polymers **Sp-Si**, **Bu-Si**, and **Bu–OH** were determined by photoelectron spectroscopy in air (PESA). Their optical bandgaps were determined from the onset of absorption by UV–vis spectroscopy (Figure S1). Their electron affinities (EA) were estimated by adding the optical bandgap to the IPs. For **Sp-Si**, **Bu-Si**, and **Bu–OH**, the IPs and EAs were estimated to be 5.1 and 3.1 eV, respectively. These values were suitable to provide a driving force for proton reduction and ascorbic acid (AA) oxidation half-reactions. IP differences between polymers measured by PESA were smaller than could be detected by the instrument (about 100 meV), and differences in optical bandgap were negligible (Figure S1), as would be expected given they all possessed the same conjugated backbone. This allowed the optical absorption and band positions to be held constant, as other variables were modified.

### Porosity and Relative Hydrophilicity

**Figure 3:**
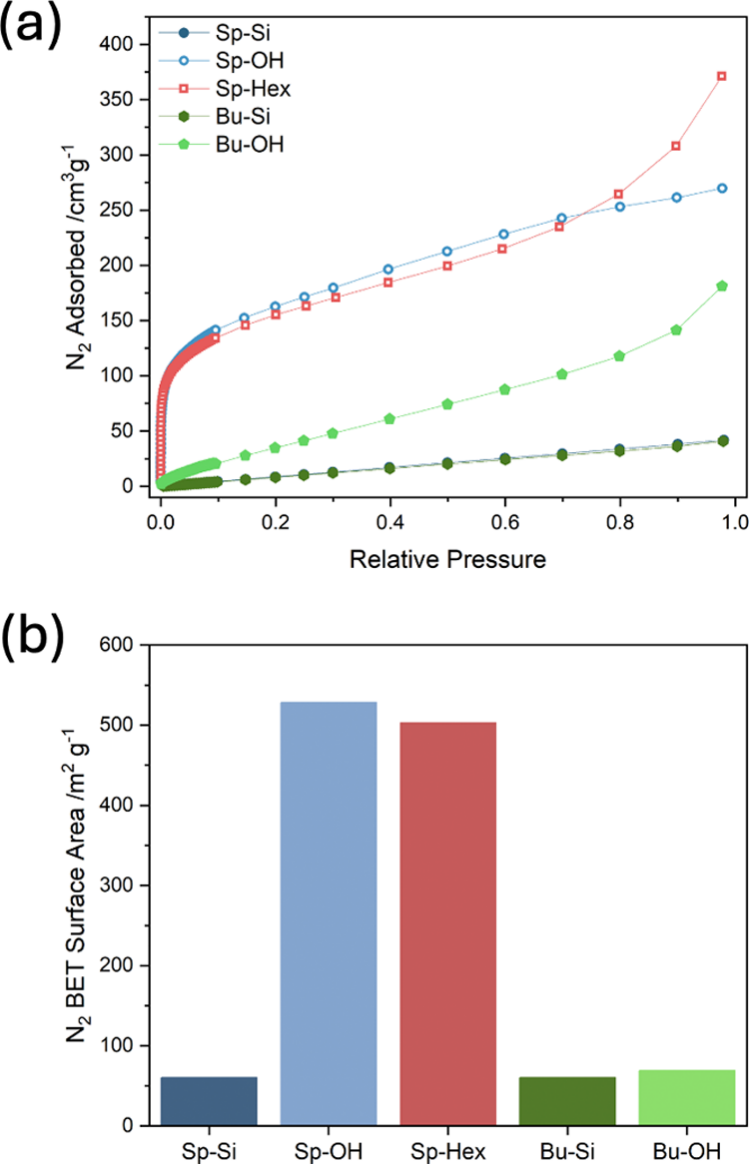
(a) N_2_ adsorption isotherms for all polymers with desorption omitted for clarity (note that **Sp-Si** and **Bu-Si** overlap). (b) BET surface areas calculated from the corresponding N_2_ adsorption isotherms for the linear regions of the BET plots between relative pressures of 0.01 and 0.03.

N_2_ sorption isotherms were measured to estimate the apparent BET surface areas of the polymers shown in Figure 3 and summarized in Table 2.

The deprotection of **Sp-Si** to form **Sp–OH** was accompanied by a large increase in surface area from 60 to 528 m^2^ g^–1^ with the latter material exhibiting a largely type-I adsorption isotherm following deprotection (Figure 3). This suggests that the large flexible trihexyl silyl ether side chains were intercalating into any pore sites that could arise from inefficient packing due to the *tert*-butyl spirofluorene groups, and their removal allowed unimpeded pore formation. This “pore blocking” effect by large side chains has been reported for other porous materials.^75,76^

**Figure 4:**
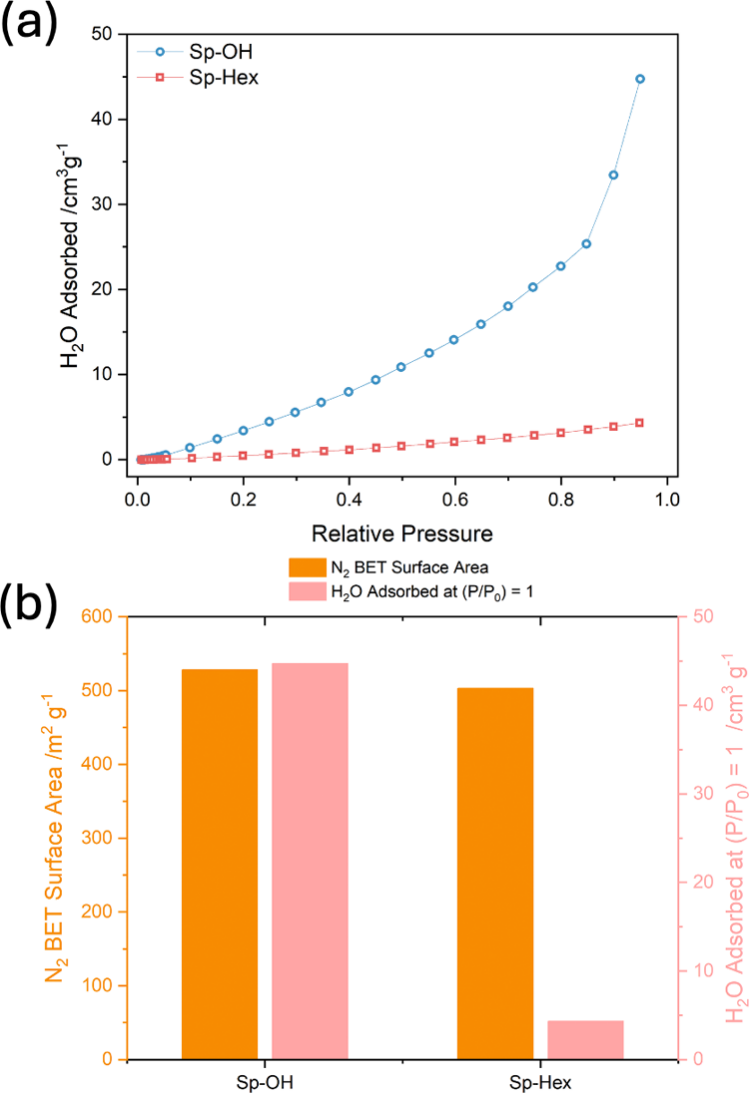
(a) Water adsorption isotherms for **Sp–OH** and **Sp-Hex** with desorption omitted for clarity. (b) Comparison of BET surface areas calculated from the N_2_ isotherms (orange left axis) and the maximum volume of water adsorbed at (*P*/*P*_0_)=1 (pink right axis).

**Sp-Hex** showed a similar BET surface area to **Sp–OH** of 503 m^2^ g^–1^ owing to its less sterically bulky hexyl side-chain which is more similar in size to the ethyl alcohol side-chain of **Sp–OH**, compared to the bulky multiple side chains on **Sp-Si**. Given that the only significant difference between **Sp-Hex** and **Sp–OH** was the presence of alcohol moieties, comparison of these polymers allowed investigation of the effects of relative hydrophilicity for polymers of a very similar N_2_ BET surface area. Given that **Sp–OH** and **Sp-Hex** were porous, water sorption isotherms were undertaken to measure water affinity (Figure 4a). As can be seen from the scale bars, water sorption is less pronounced than N_2_ sorption, indicating that water molecules do not penetrate the whole volume estimated by BET. When comparing these data sets (Figure 4b), the alcohol-bearing **Sp–OH** showed 11.3 times the maximum water sorption compared to nonpolar **Sp-Hex**, despite comparable N_2_ sorption BET surface areas. This suggests differential access of water to the porous volumes in the two polymers for photocatalytic applications caused by the presence of alcohol moieties.

Finally, as predicted, **Sp-Si**, **Bu-Si**, and **Bu–OH** showed BET surface areas of 60, 60, and 69 m^2^ g^–1^, respectively, displaying isotherms characteristic of nonporous materials (Figure 3). This is because **Sp-Si** possesses large conformationally flexible trihexyl silyl ether protecting groups. Meanwhile, **Bu-Si** and **Bu–OH** lack the orthogonal *tert*-butyl spirofluorene groups necessary to induce intrinsic microporosity in the first place. In order to characterize the relative water affinities of these soluble and nonporous polymers, films were spin-coated onto glass and contact angles were measured, shown in Table 1. This shows that the silyl ether-protected polymers are relatively hydrophobic, and the deprotection of **Bu-Si** to form **Bu–OH** is accompanied by a modest decrease in the contact angle by 14°, consistent with an increase in relative hydrophilicity and wettability.

**Table 1:** Contact Angles of Water Droplets on Polymer Films Spin-Coated onto Glass from 5 mg mL^–1^ Chloroform Solutions, Averaging across Two Samples with Three Repeats (Details in the SI)

**polymer film**	**contact angle/°**	**standard deviation/°**
Sp-Si	99.4	0.7
Bu-Si	100.9	1.2
Bu–OH	87.3	1.1

### Photocatalytic Activities and Particle Size Distributions

**Figure 5:**
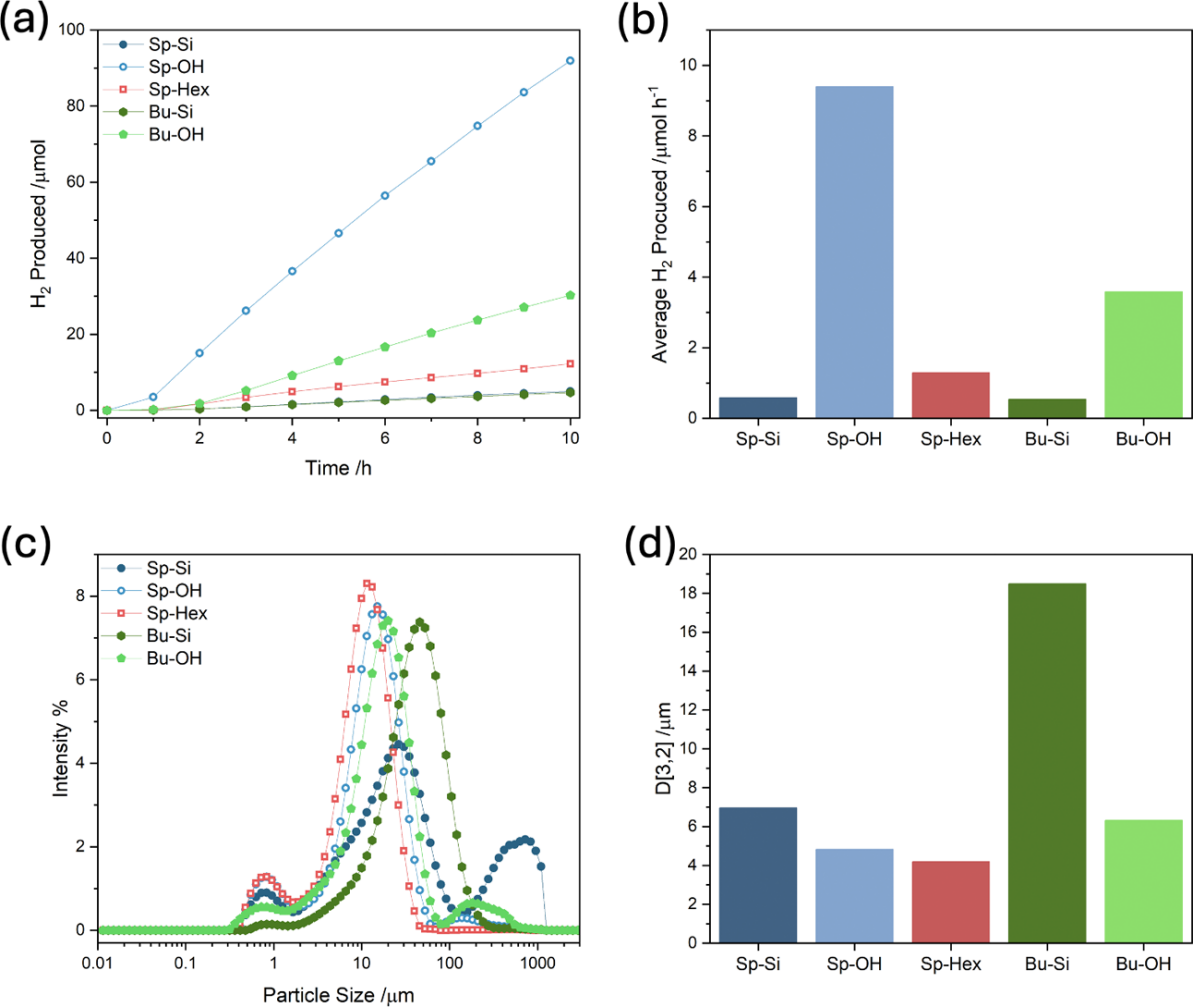
(a) Cumulative H_2_ produced over 10 h for 5 mg of polymer dispersed in NMP/water, 0.2 M neutralized AA with 3 wt % photodeposited Pt relative to polymer (full conditions in the SI, note **Sp-Si** and **Bu-Si** overlap). (b) Average H_2_ production rates between 4 and 10 h (illumination area 15.9 cm^2^). (c) Static light scattering particle size distributions for aqueous suspensions of polymers prepared in the same manner as photocatalytic testing. (d) Surface weighted mean particle sizes D[3,2] calculated from the static light scattering particle size distributions.

Having established the polymers’ porosities and relative hydrophilicities, samples were prepared for photocatalytic testing by dispersing the polymers in water/NMP mixtures, using neutralized ascorbic acid as electron donor and Pt as cocatalysts under visible light illumination (conditions in SI). The use of cosolvents such as NMP is common in photocatalytic material testing owing to its ability to aid polymer dispersion and hinder aggregation.^22,77−79^ Control reactions using NMP without semiconductor showed no H_2_ production, and use of NMP as a cosolvent has been reported elsewhere.^80^ H_2_ evolution profiles for the polymers tested under the same conditions are shown in Figure 5a. Because the *internal* interface area was the primary variable under investigation, it was also important to take account of the *external* interface areas of the polymer dispersions under photocatalytic conditions, to ensure that this was not the dominant factor driving any differences in photocatalytic activity. Static light scattering measurements were undertaken to characterize the particle size distributions for polymers dispersed in the same manner as they were tested, shown in Figure 5c, with the surface weighted mean particle sizes, D[3,2] shown in Figure 5d.

### Correlating Properties to Activity

The polymer properties and activities are summarized in Table 2.

**Table 2:** Summary of Properties and Photocatalytic Rates of H_2_ Production, as Plotted in Figures 3–5

**polymer**	**N**_**2**_**BET surface area/m**^**2**^**g**^**–1**^	**H**_**2**_**O adsorbed at (*P*/*P***_**0**_**)=1/cm**^**3**^**g**	**D[3,2] mean particle size/μm**	**average H**_**2**_ **produced/μmol h**^**–1**^
Sp-Si	60		7.0	0.58
Sp–OH	528	45	4.8	9.40
Sp-Hex	503	4	4.2	1.29
Bu-Si	60		18.5	0.54
Bu–OH	69		6.3	3.58

For all of the polymers, greater relative hydrophilicity correlates with increased activity. For the hydrophobic polymers lacking alcohol moieties with the same conjugated backbone, **Sp-Si**, **Sp-Hex**, and **Bu-Si**, the most active was **Sp-Hex** which was porous, though it also had the smallest particle size.

The effect of deprotection on the photocatalytic activity was dramatic: both silyl ether protected polymers **Sp-Si** and **Bu-Si** exhibited significantly increased photocatalytic activity following deprotection. However, in each case different variables were altered. When **Sp-Si** was deprotected to form **Sp–OH**, activity increased by a factor of 16.2, from 0.58 to 9.40 μmol h^–1^ (with **Sp–OH** giving an EQE of 0.11% at 550 nm, Figure S7). Here, porosity increased by a factor of 8.8, from 60 to 528 m^2^ g^–1^, while at the same time average particle size decreased by a factor of 0.7, from 7.0 to 4.8 μm (and hence external interface area increased), as would be expected given increased hydrophilicity aids aqueous dispersibility.^20,60,81^ In this case, the large increase in activity is attributed mainly to a combination of increased porosity and relative hydrophilicity, along with the decreased particle size. In the case of the nonporous polymer **Bu-Si** and its deprotected analogue **Bu–OH**, deprotection caused a proportionally smaller increase in activity, by a factor of 6.6, from 0.54 to 3.58 μmol h^–1^, while porosity did not change (with them being both nonporous), and particle size decreased slightly by a factor of 0.9, from 18.5 to 16.3 μm. Meanwhile the average contact angle of the films with water decreased by 14° following deprotection, showing an increase in relative hydrophilicity. This therefore links the activity increase following deprotection of Bu-Si to form Bu–OH to greater relative hydrophilicity and a decreased particle size.

Comparison of **Sp-Hex** to **Sp–OH** is particularly insightful in deconvoluting the influence of the three parameters, because their aqueous dispersions exhibit similar particle sizes, with averages of 4.8 and 4.2 μm, respectively, and they display similar N_2_ sorption BET surface areas of 528 and 503 m^2^ g^–1^. The only major difference between these polymers is the presence or absence of alcohol moieties, which manifests itself in the large differences in water sorption, where the maximum volume of water adsorbed was 4 cm^3^ g^–1^ for **Sp-Hex** and 45 cm^3^ g^–1^ for **Sp–OH**. Crucially, having controlled for the other variables, it is this difference in relative hydrophilicity to which the large difference in photocatalytic activity can be attributed here, being 7.3 times greater in **Sp–OH** compared to **Sp-Hex**.

### Transient Absorption Spectroscopy (TAS)

**Sp–OH** and **Sp-Hex** dispersions were an attractive choice for further investigation, given their shared conjugated backbones, N_2_ adsorption BET surface areas, and dispersion average particle sizes were similar. To gain more mechanistic insight into the effects of wettable porosity on photocatalytic activity, **Sp–OH** and **Sp-Hex** dispersions were prepared under the electrolyte conditions used for photocatalysis and probed by fs-ps transient absorption spectroscopy (TAS).

**Figure 6:**
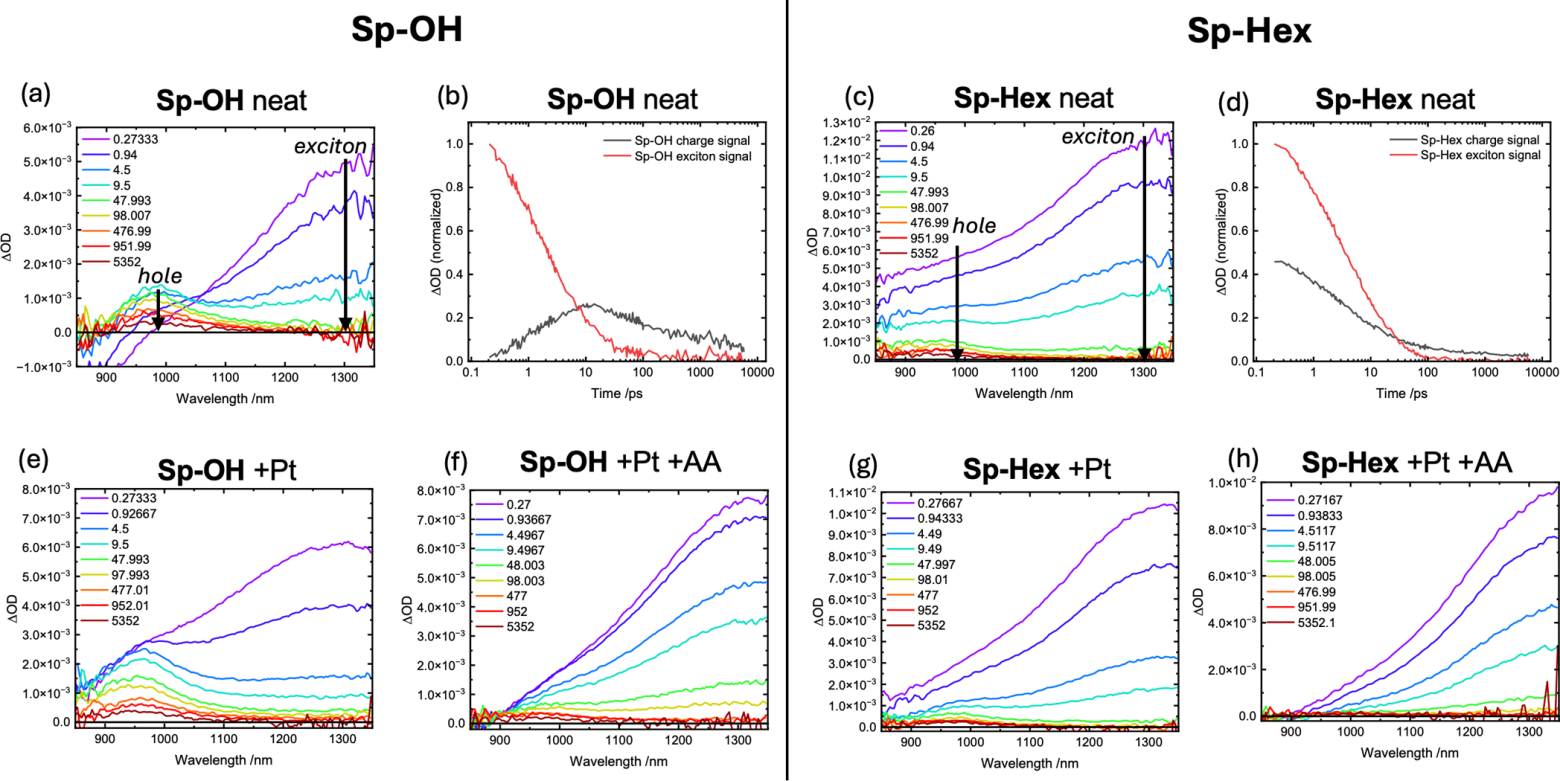
fs-ps transient absorption spectroscopy (TAS) of **Sp–OH** and **Sp-Hex** dispersions under different conditions. (a,c) neat materials in electrolyte, with the assignment of exciton and polaron signals (see Figure S10 for fluence dependence). (b,d) corresponding time traces of the exciton and charge signal following spectral deconvolution by global analysis, normalized to exciton at 0.2 ps. (e,g) respective TAS spectra with Pt added, illustrating different influence on the charge signal (see also Figure S9). (f,h) TAS spectra in the presence of both Pt and AA.

In all cases, a broad NIR photoinduced signal was obtained and characterized by two overlapping contributions: a photoinduced absorption signal peaking at ∼1300 nm, which decays within 100 ps, and a longer-lived signal peaking at ∼970 nm (see Figure 6a,c for **Sp–OH** and **Sp-Hex***neat* dispersions). Following an earlier study employing a related IDT-based polymer, these two signals can be assigned to excitons and hole polarons (charge) respectively. Their deconvoluted kinetics are shown in Figures 6b,d.^30^ The assignment of the ∼970 nm signal to holes was confirmed by photoinduced absorption spectroscopy (PIAS) measurements on the seconds time scale, where the charge accumulation properties are probed (Figure S11 and S12). This hole signal is present in both **Sp–OH** and **Sp-Hex***neat* dispersions (with only water and NMP present) on ps time scales but has a distinct evolution for the two polymers: in **Sp–OH**, it shows rise kinetics on the same time scale as exciton decay (t_50%_ ∼ 2 ps), as observed previously for g-IDTBT nanoparticles,^30^ in contrast to **Sp-Hex** where hole formation is observed within our instrument response (<250 fs). TAS and PIAS measurements in the presence of AA showed this photoinduced signal to be effectively quenched, confirming its assignment to hole polarons (Figure S9a,b). We note that our observation of ultrafast hole polaron formation is most likely associated with the formation of electron/hole pairs, with the electron polaron signal being too weak or outside our spectral window to be resolved in our studies. The hole polaron signal showed intensity-independent decay kinetics on the nanosecond time scale (Figure S10), indicative of the monomolecular (geminate) recombination of bound electron/hole pairs.

Measurements in the presence of Pt only (photodeposited as in photocatalytic conditions, but with the AA removed) reveal its crucial role (Figure 6e,g): in **Sp–OH** Pt further increased the hole polaron signal showing it selectively collects electrons on the ultrafast time scale. In contrast, for **Sp-Hex**, the addition of Pt results in a quenching of the hole signal in comparison to the neat material, indicating that Pt also acts here as a hole acceptor and thus a recombination site for electrons and holes. We note that we have highlighted the potential for Pt to act as a recombination center for photoexcitations in organic semiconductors previously.^82^ The ability of Pt to selectively accept electrons from **Sp–OH,** increasing the yield of hole polarons, is likely a key factor in its higher photocatalytic activity, as we discuss further below. Also, the accumulated holes appear to be more stable in **Sp–OH** in the presence of Pt, being beneficial for continuous reaction with replenished AA donors (see Figure S12).

Figure 6f,h shows the TAS spectra with both Pt and AA, representing photocatalytic conditions: in both materials, the hole polaron signal is significantly reduced, indicative of efficient, ultrafast hole scavenging. An analogous quenching of the hole polaron signal by AA was also observed in the absence of Pt (Figure S9). The ability of AA to scavenge holes on the ps time scale (see also Figure S9) is striking and distinct from that we have reported previously for nonporous organic nanoparticles.^30,37^ It can most obviously be attributed to the high porosity of both **Sp-Hex** and **Sp–OH,** enabling AA diffusion into the interior of the polymer particles, as we discuss further below.

These kinetic studies provide a clear understanding of the ultrafast photophysics of the **Sp-Hex** and **Sp–OH** photocatalysts. Both neat materials exhibit significant ultrafast charge generation, as indicated by our observation of hole polaron photoinduced absorption. In both materials, AA can extract these holes on the ultrafast (<1 ps) time scale. The lower photocatalytic activity of **Sp-Hex** compared to **Sp–OH** appears to result in particular from Pt acting as a selective electron acceptor/catalytic site on **Sp–OH** but as a nonselective quencher of both electrons and holes on **Sp-Hex.**

## Discussion

All polymers tested showed a strong correlation between wettable porosity and activity. This is consistent with other studies where porosity and hydrophilicity coincide,^51,60^ while in this case the variables of relative hydrophilicity and porosity have been varied independently as far as possible, enabling effects to be more directly evaluated. Furthermore, this study highlights that water sorption isotherms are key measurements that bear a much stronger connection to photocatalytic activity than N_2_ sorption BET surface area measurements alone, given it links both porosity and affinity to the aqueous electrolyte.

TAS experiments detected the formation of holes in both **Sp–OH** and **Sp-Hex** when measured as dispersions in water and NMP alone, with no AA or Pt present, and low levels of residual Pd (less than 3 ppm by ICP-MS, Table S3). In nonporous nanoparticle systems stabilized by surfactants, the AA reaction with holes often takes place on the μs time scale,^30,37^ presumably because excitons and holes are generated at greater distances from the semiconductor–electrolyte interface where reaction with AA or separation by Pt is possible, and the presence of surfactants could hinder the kinetics of the AA reaction. However, for porous and more electrolyte-infiltrated systems such as **Sp–OH** and **Sp-Hex**, AA is now shown to react with hole polarons orders of magnitude quicker, on the ps time scale. This direct observation is possible due to the pronounced hole signals on these materials being affected by AA and suggests that hole polarons are formed in regions readily accessible to AA, which sit poised to react almost immediately. Indeed, rapid AA quenching of excitons has been reported in a different porous system.^83^ Given that AA dissolved in the electrolyte can only access regions accessible to the electrolyte itself, it follows that hole polarons are mainly being generated close to these accessible regions or that most of the volume is sufficiently accessible here to extract charges. These two features may be linked: it is possible that the increased dielectric environment provided by water helps to facilitate intrinsic charge generation and accumulation in these porous systems in the first place. Nonetheless, the differential water access between **Sp–OH** and **Sp-Hex** does not explain why **Sp-Hex** shows more rapid hole generation. It could be that the hole polarons in **Sp-Hex** are mainly generated in mesopores or on the outer particle surface, where water and AA can also access. On the other hand, the hole generation in **Sp-Hex** could be driven by intrinsic defect states where holes can reside since their appearance is immediate and less linked to decay of excitons on the neat material, as observed on **Sp–OH** (Figure 6 and Figure S9), where the charges appear to be generated from excitons. Despite the possibly stronger ability to generate holes on **Sp-Hex** intrinsically, the time-resolved spectroscopic measurements and the H_2_ production measurements point toward the fact that they are less catalytically productive on this material.

The presence of photodeposited Pt in **Sp–OH** enhanced the amplitude and elongated the lifetime of the hole signal on the fs-scale, while keeping it stable on the second scale (Figure S11 and S12). On the other hand, their presence attenuated its amplitude and shortened its lifetime in **Sp-Hex** on both fs-ps and second time scales, indicating its role as a charge recombination center, which can explain the greater activity of **Sp–OH**. These differences may be due to the wettability of the materials since Pt^0^ grows during photodeposition from dissolved H_2_PtCl_6_ at interface areas with electrolyte where electrons accumulate. If these electron accumulation interface sites are more accessible to dissolved H_2_PtCl_6_ (by increased hydrophilicity), Pt may distribute more selectively at electron accumulation sites and not interfere with the holes that are being quenched (beforehand) by AA. This important and unexpected finding potentially suggests another benefit of the inclusion of hydrophilic functional groups: in photocatalysts, these surface groups may have a beneficial influence on the Pt photodeposition process to occur more selectively at places where reactive electrons reside (and not holes), though further investigation is needed to ascertain this effect more generally.

Finally, additional photocatalysis tests varying the AA concentration (Figure S8) showed a strong dependence of activity on the AA concentration. This suggests that in these systems where excitons are already converted into charges, fast and continuously efficient hole extraction is a bottleneck in the photophysical mechanism chain. This highlights its crucial role in sustained high photocatalytic efficiency, which seems to require efficient replenishing of the donor, and differences in activity could be due to mass transport effects and pore utilization. Indeed, other studies on porous photocatalysts have claimed that beyond a critical porous channel depth in crystalline materials, mass transport becomes prohibitive and additional interior surface area in this region is photocatalytically unproductive.^83^ This effect may be more pronounced in PIMs due to their amorphous structures.

## Conclusions

It was found that N_2_ sorption BET surface areas for a series of PIMs were poorly predictive of their photocatalytic activity when other factors were accounted for; rather, the presence of wettable porosity had a much stronger link to activity. Water sorption isotherms were shown to exhibit a much stronger correlation and hence point to only wettable areas being relevant for photocatalysis and not those measured by N_2_ gas sorption. TAS investigation revealed that there was intrinsic and fast (<10 ps) charge generation in both **Sp–OH** and **Sp-Hex**, and that the detectable hole polarons were able to react with AA extremely rapidly, on the 200 fs-10 ps time scale, and hence much faster than typically observed in nonporous organic photocatalysts. Our data suggest the generation of charges at regions readily accessible to AA in the porous structures – being a generally beneficial property for photocatalysis and for reducing its bottleneck – charge and exciton recombination. In addition, the greater propensity of the Pt cocatalyst in **Sp-Hex** to act as a site of electron and hole recombination may partially account for its lower activity compared to **Sp–OH**, with differences in mass transport in the two porous structures being a likely further cause of the differences in activity based on the difference in water sorption isotherms. This study reaffirms the growing transition from hydrophobic organic semiconductor photocatalysts toward increasingly hydrophilic ones, to capitalize on the potential photophysical effects of water’s increased dielectric response on charge generation and stabilization,^30^ as well as the ability to enable rapid reaction with species in the electrolyte. While porous hydrophilicity is a desirable property for single semiconductor systems, which may be achieved by other cross-linked network polymers, its incorporation into PIMs may be particularly advantageous when permitting sufficient solution processability to enable the codissolution of such polymers whose FMOs are offset to permit the formation of type-II heterojunction systems,^84^ which could further hinder recombination and enhance polaron lifetime by spatial separation of electrons and holes onto the different domains.^30^ More speculatively, utilization of differential access to pores by pore size or surface engineering could potentially allow selective interactions with redox shuttles in Z-schemes to hinder unfavorable recombination for use in overall photocatalytic water splitting.

## Supporting Information Available

All experimental methods and characterization (PDF)

## ABBREVIATIONS

AA: ascorbic acid
BTz: benzotriazole
IDT: indacenodithiophene
IP: ionization potential
NIR: near-infrared
PESA: photoelectron spectroscopy in air
PIAS: photoinduced absorption spectroscopy
PIM: polymer of intrinsic microporosity
TAS: transient absorption spectroscopy
