# Supplementary material for: Correlation between the Molecular Properties of Semiconducting Polymers of Intrinsic Microporosity and Their Photocatalytic Hydrogen Production
Source: J Am Chem Soc. 2024 Oct 30;146(45):30813–23. doi: 10.1021/jacs.4c08549 (PMC11565637; doi:10.1021/jacs.4c08549)
Supplement: Supplementary file 1 — ja4c08549_si_001.pdf [file ja4c08549_si_001.pdf]

SUPPORTING INFORMATION FOR: CORRELATION BETWEEN THE MOLECULAR PROPERTIES OF SEMICONDUCTING POLYMERS OF INTRINSIC MICROPOROSITY AND THEIR PHOTOCATALYTIC HYDROGEN PRODUCTION

Benjamin J. Willner,* Catherine M. Aitchison,* Filip Podjaski,† Wanpeng Lu,* Junfu Tian,* James R. Durrant† and Iain McCulloch*‡

*Department of Chemistry, Oxford University, Chemistry Research Laboratory, 12 Mansfield Road, Oxford, OX1 3TA, UK

‡Andlinger Center for Energy and the Environment and Department of Electrical and Computer Engineering, Princeton University, Princeton, NJ, 08544, USA

†Department of Chemistry and Centre for Processable Electronics, Imperial College London, 80 Wood Lane, London W12 0BZ, U.K.

1. Characterisation of Solution-Processable Polymers
2. Silyl Ether Deprotection
3. Sample Preparation and Photocatalytic Testing
4. External Quantum Yield
5. Ascorbic Acid Concentration Study
6. Additional Illumination Area Normalised Photocatalytic Activity
7. Additional TAS Data
8. Redox Potential Estimates for Proton Reduction and Ascorbic Acid Oxidation
9. Sample Preparation for Laser Diffraction Analysis to Determine Particle Size
10. Inductively Coupled Plasma Mass Spectrometry Residual Pd Measurements (ICP-MS)
11. Isotherm Measurements
12. Calculated Pore Size Distributions
13. Synthesis and compound characterisation

1. Characterisation of Solution-Processable Polymers

Polymer films were spin coated onto glass slides from 5 mg mL^{-1} chloroform solutions, which were made up by sonicating and stirring at 60 °C for 1 h to aid dissolution. These spin cast films were used to measure thin film ultraviolet-visible spectra (UV-Vis) using a Shimadzu UV-1800 spectrometer, and photoemission spectroscopy in air (PESA) spectra using a KP Technology APS02 system. The intercept of the PESA signal to the baseline was used to estimate the ionisation potential (IP) of the polymer films. The optical bandgaps were estimated using the intercept of the absorption to the baseline from the UV-vis spectra. Subtracting the optical bandgap from the ionization potential energy was taken as an estimate of electron affinity (EA). Molecular weight gel permeation chromatography (GPC) data was acquired using an Agilent 1260 Infinity II series instrument using chlorobenzene as the solvent. Energy dispersive X-ray spectroscopy (EDX) measurements were conducted on a Carl Zeiss Merlin SEM with an Oxford instruments Xmax 150 EDX. Samples were measured at 30 kV and 30 nA beam current.

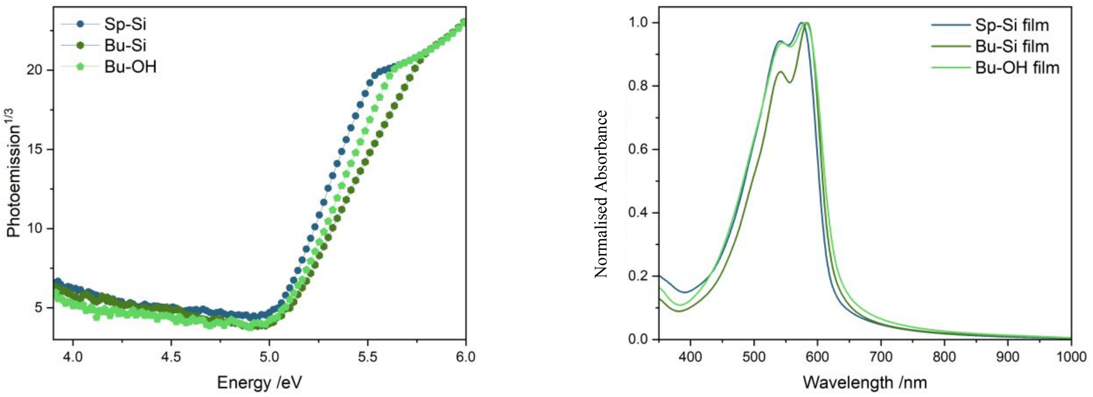

Figure S1. Photoemission spectroscopy in air (PESA) and ultraviolet-visible (UV-vis) spectroscopy for thin films of **Sp-Si**, **Bu-Si** and **Bu-OH**.

Polymer	Absorbance Onset / nm	Absorption Peak(s) /nm	Optical Bandgap /eV	(PESA) IP /eV	EA /eV	M_N /g mol^{-1}	M_W /g mol^{-1}	Poly-dispersity
Sp-Si	621	542, 575	2.0	5.1	3.1	24107	61073	2.53
Bu-Si	625	542, 583	2.0	5.1	3.1	27027	45072	1.67

Table S1. Summarised characterisation data for Sp-Si and Bu-Si obtained from UV-vis spectroscopy, PESA and GPC in chlorobenzene

2. Silyl Ether Deprotection

In a large microwave vial, 1.26 g TBAF trihydrate was dissolved in 20 mL THF and 1 mL acetic acid (to neutralise the basicity of the TBAF). The sample of polymer was added to this, sealed and sonicated for 30 min and stirred at 80 °C overnight. This was then poured into methanol and filtered, washing with more methanol and acetone to remove any residual TBAF or silyl groups and dried in a vacuum oven for several hours.

Figure S2 shows following TBAF treatment, silyl protons are absent while there is retention of aromatic protons indicating no degradation to the conjugated backbone, and the appearance of a new peak at 2.87 ppm, which relative to the peaks at 4.63, 4.02 ppm integrates to a ratio of 2:2:1, consistent with the presence of an alcohol in **Bu-OH**.

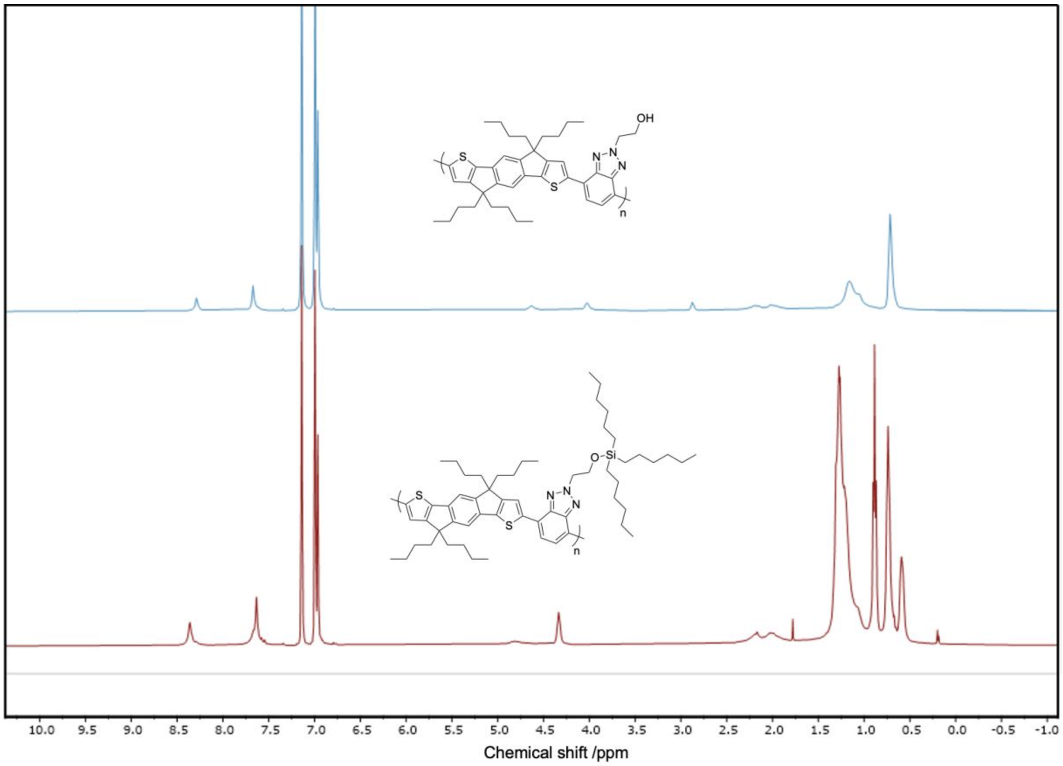

Figure S2. 400 MHz ^{1}H NMR in C_6D_5Cl of **Bu-Si** (bottom) before TBAF treatment and **Bu-OH** (top) after TBAF treatment.

Sp-OH was not soluble enough for NMR analysis. Instead, the presence of Si in the protecting group was used as an atomic label, and its disappearance was used to test for deprotection, shown in Figure S3. The spectra, after normalisation to the S peak (which is unaffected during deprotection), show the disappearance of the Si signal to below detectable levels, as well as an attenuation in the C signal which is consistent with the loss of the silyl ether functional groups in the deprotected polymer.

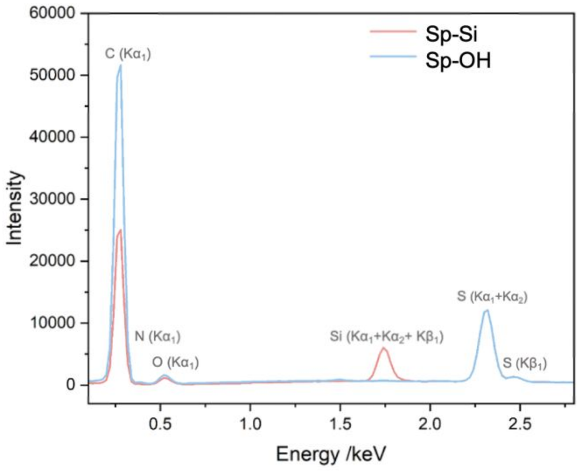

Figure S3. Energy dispersive X-ray (EDX) spectrum for **Sp-Si** (before TBAF treatment) and **Sp-OH** (after TBAF treatment) measured over three different areas of polymer chosen at random, normalised to the S peak (which is unaffected during deprotection),

As a further test, the monomer **13** containing the silyl ether protecting group was subjected to the same deprotection conditions as the polymer Sp-Si bearing this group. At the same time, 2H-benzotriazole-2-ethanol was separately synthesised and the NMR spectrum recorded shown in Figure S4 (top). Following deprotection of **13** an extra peak g arises, where peaks b, c and g integrate to a ratio of 2:2:1. Comparison of these NMR spectra support the identity of deprotected **13** as 2H-benzotriazole-2-ethanol. Combined with the EDX data, as well as the stark reduction in solubility of **Sp-OH** compared to **Sp-Si**, supports the identity of TBAF treated **Sp-Si** as **Sp-OH.**

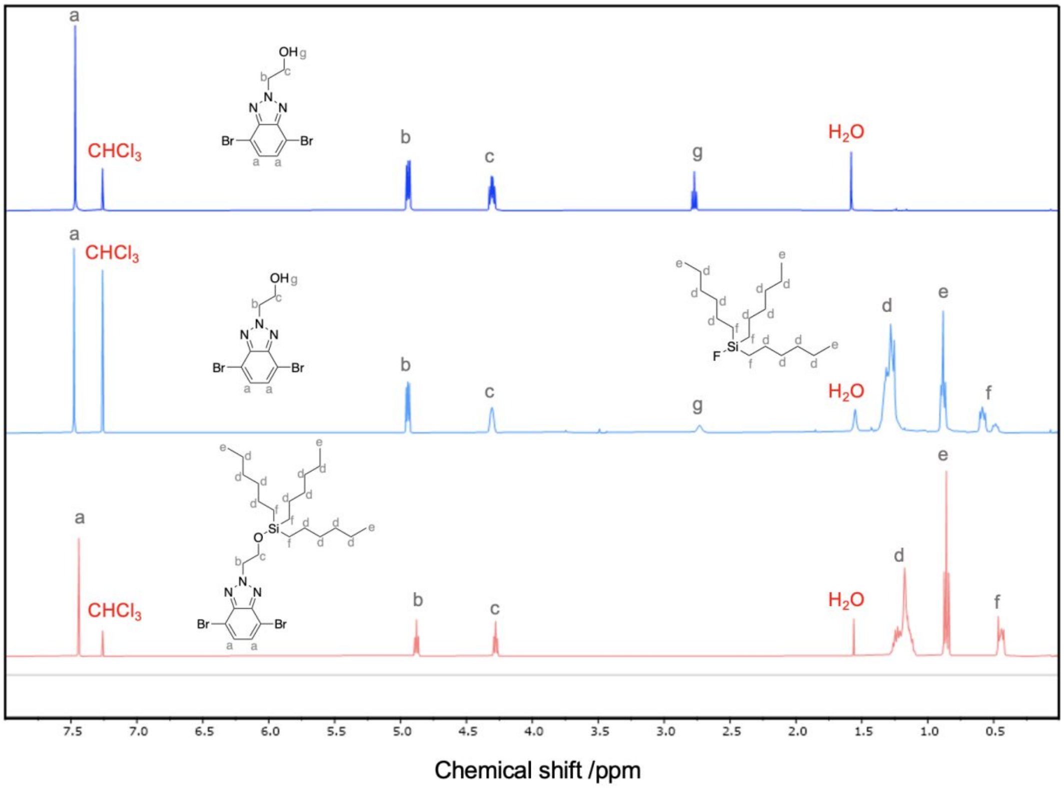

Figure S4. 400 MHz ^{1}H NMR in $CDCl_3$ showing silyl ether monomer **13** (bottom), compared to the crude product of **13** (middle) following subjection to the same deprotection conditions as the polymers using TBAF, and pure 2H-benzotriazole-2-ethanol (top) for comparison.

3. Sample Preparation and Photocatalytic Testing

To measure the amount of H_2 evolved by photocatalysts during photocatalytic testing, a bespoke setup was built. For photocatalysis measurements, 5 mg of organic semiconductor was sonicated using a probe sonicator at 200 W in 10 mL NMP for 2 minutes to either disperse or dissolve the semiconductor. This was subsequently added via pipette into an aqueous solution of ascorbic acid and H_2PtCl_6 in a sonicator bath to give 3 wt % Pt loading assuming quantitate photodeposition yield (Figure S6 below), as well as 1.6 mL 4 M NaOH solution (to give a total volume if 35 mL), so that the final pH of the water, ascorbic acid, NMP and NaOH solution was 6.43 as measured by an electronic pH probe. Once prepared, this photocatalysis mixture was added into a glass cylindrical reactor cell chilled to 15 °C and clamped to create an airtight seal. The arms on either side of the reactor cell were connected to a circulatory pump in a closed loop with a gas chromatograph (GC) to ensure even distribution of gasses for sampling. A Xe lamp solar simulator fitted with a visible light 390-740 mirror module and visible filter, calibrated to 1 Sun using a reference cell which was then directed onto the photoreactor cell where the sample sits. Once in place, the sample was put under vacuum and purged with Ar twice, then left at 450-550 mbar argon atmosphere. The measurement was begun once the light is switched on, and the GC was programmed to inject a sample of headspace gas every hour, over the course of 10 hours.

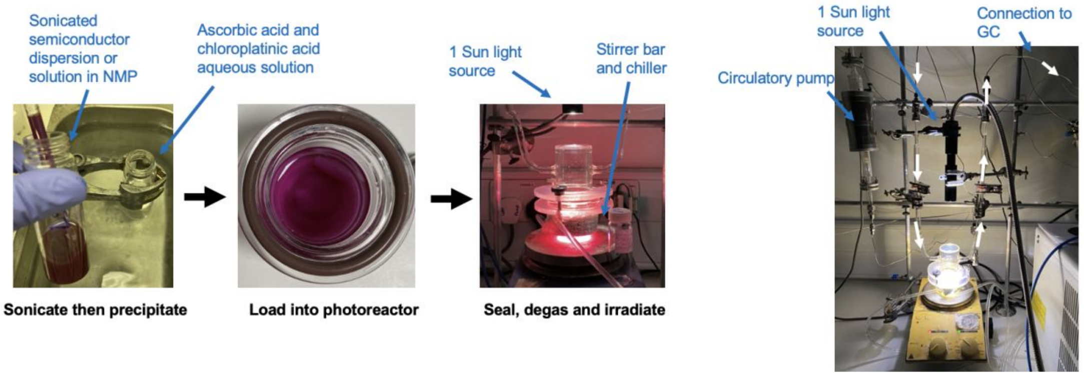

Figure S5. Left: images of the sample preparation. Right: H_2 evolution testing setup.

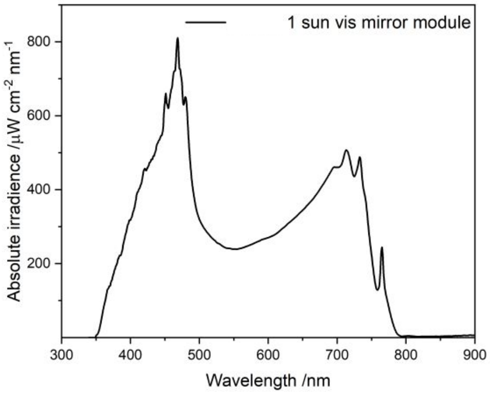

Figure S6. Spectral output of the light source used in photocatalysis experiments.

4. External Quantum Yield

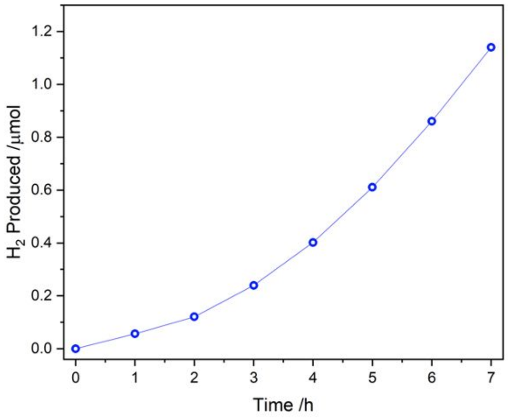

Figure S7. H_2 production profile **Sp-OH** at 550 nm monochromatized light source at 1.2 mW (1.7mW cm^{-2}).

The EQE of **Sp-OH** was measured with 0.2 M ascorbic acid prepared and tested in the same manner as described above, except it was irradiated using a filter to monochromatize the light source centred at 550 nm. Using a Thorlabs light intensity probe set to 550 nm, the illumination power was measured at the same position the sample sits during testing. The incident power was measured to be 1.2 mW. The light intensity detector aperture was 0.709 cm^2, making the light intensity received by the sample per unit area 1.693 mW cm^{-2}. Given that the (uniform) illuminated photocatalysis cell was 15.9 cm^2, the power incident on the photocatalyst was 26.919 mW. Each 550 nm photon possesses 3.61168 x10^{-19} J of energy, which meant the photon flux incident on the sample was 7.4533 x10^{16} photons s^{-1}. Dividing by Avogadro's constant to get the incident flux of photons in moles gives 1.237 x10^{-7} mol s^{-1}. The photocatalyst test was conducted for 7 h, and the H_2 production rate was calculated between 4 and 7 h (where it was constant following an induction period related to photodeposition) to be 0.246 µmol h^{-1}. This equates to 6.833 x10^{-11} mol s^{-1}. Given two photons are required to produce 1 mole of photons, the external quantum yield was calculated using the following equation:

$$EQE\,\% = \frac{2\, n_{H_2\, produced}}{n_{incident\ photons}} \times 100$$

Using the above data, EQE = 0.11 % at 550 nm monochromatic light for **Sp-OH** at pH 6.43 and 0.2 M ascorbic acid with 3 wt % Pt photodeposition precursor added.

5. Ascorbic Acid Concentration Study

In order to further investigate the role of ascorbic acid for the most active porous system possessing hydrophilic alcohol moieties, **Sp-OH** was tested at reduced ascorbic acid concentrations, while adjusting pH accordingly with NaOH to maintain a constant pH.

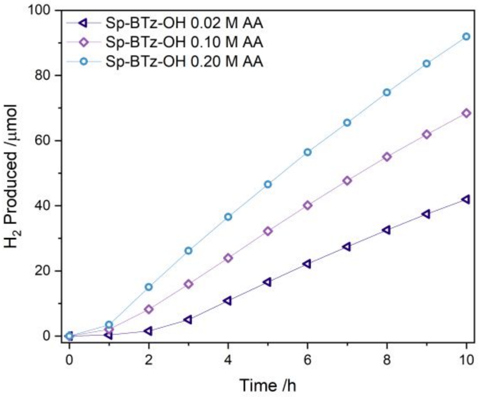

Figure S8. Photocatalytic activity as function of ascorbic acid electron donor concentration while keeping pH constant.

6. Additional Illumination Area Normalised Photocatalytic Activity

Polymer	Average H_2 Produced Neutral / µmol h^{-1}	Average H_2 Produced Neutral / µmol h^{-1} cm^{-2}
Sp-Si	0.58	0.036
Sp-OH	9.40	0.591
Sp-Hex	1.29	0.081
Bu-Si	0.54	0.034
Bu-OH	3.58	0.225

Table S2. Average rates of H_2 evolution for 5 mg dispersed in 10 mL NMP and 25 mL neutralised ascorbic acid (0.2 M) with and 3 wt% in situ photodeposited Pt from H_2PtCl_6 at the conditions described above. The illuminated photocatalysis cell had an area of 15.9 cm^2.

7. Transient Absorption Spectroscopy

Sample preparation:
For preparation of the Pt deposited samples, 3.5 mg polymer was dispersed in 2 mL NMP sonicated and pipetted into 5.0 mL water with 27 µL H_2PtCl_6 and 0.25 g ascorbic acid. This was irradiated at 1 Sun under stirring in a 35 ml vial with a small stirrer bar for 5 h. It was poured through a cellulose filter and washed with water several times, making sure to not let the polymer dry out. After washing the polymer was transferred to a vial, and a mixture of 1 mL NMP and 2.5 mL water was added to each, then sonicated at 200 W for 1 minute. The neat material was used as prepared and dispersed an NMP/water ratio of 1/2, as for the photocatalytic experiments. For TAS measurements, the samples had to be diluted to an optical density (O.D.) being in the range of 0.35 to 0.4. at 6100 nm, where the samples were excited. Dilution was realized using the same ratio of NMP and water. When ascorbic acid was added to the experiments, its concentration was kept at 0.2 M as in the photocatalytic experiments, while being neutralized by NaOH to neutral conditions. All samples were measured in ambient conditions since purging in the cuvettes significantly altered the particle density and their agglomeration behaviour.

Fs-ns transient pump-push optical absorption spectroscopy:
Ultrafast TAS measurements of the polymers dispersed in water and NMP were carried out by using a amplified Ti:sapphire laser (Solstice, Spectra Physics), with a 800 nm laser pulse (92 fs, 1 kHz repetition rate). The pump laser pulse (here at 610 nm excitation wavelength) is generated through an optical parametric amplifier (TOPAS Prime, Light Conversion) and a frequency mixer (NirUVis, Light Conversion). The probe pulse measures the sample's absorbance change at specific time delay is generated through a delay stage, which delay it by an adjustable period (maximum of 6 ns) relative to pump pulse. The probe light used in these experiments is generated by focusing the probe pulse into a sapphire crystal, generating a broad band NIR signal. The change in absorbance is recorded in the region between 850−1350 nm. The probe pulse is further divided before the sample into two pulses, where one is use as reference to compensate for intensity fluctuations. Both pulses are directed to separated multichannel spectrometer (Si or InGaAs sensor). To acquire pump-probe data, the continuum probe pulse on the samples is spatially overlapped with the pump pulse by maximizing the exciton signal. Every 2nd pump pulse was chopped by a synchronized chopper such that the ground state and excited state of the sample are probed in an alternating manner, and the absorbance difference is

calculated from these two signals. Pulse energies were measured using an energy meter (OPHIR Photonics, VEGA P/N 7Z01560), using a 500 μm diameter aperture. All suspensions were prepared to absorb an equal number of photons (absorbance of 0.35-0.4 at the excitation wavelength, 610 nm) in 2 mm quartz cuvettes (Hellma Analytics).
The deconvoluted transient kinetics for the exciton and polaron of our materials were determined with spectral models for these two species using a custom global analysis code. To this end, the neat charge signal after exciton decay into charges was used as input parameter for the charge, being adjusted freely by the code (no significant modifications were needed). The exciton signal input was generated from either the initial exciton data not containing charges, or by subtracting the charge signal from initial TAS data containing the charge and the exciton at early time scales (typically 1ps or faster). This method could be used consistently for all samples, since the charge signal appeared to be centred at approx. 970 nm, and the excitons at approx. 1300 nm. Signal attribution for the charge was verified by Photo-Induced Absorption Spectroscopy (PIAS) data, as explained in the main text.

Photo-Induced Absorption Spectroscopy (PIAS):
PIAS analysis was carried out on a modified microsecond—second TAS set-up. A high-power LED (*New Energy*, LST1-01H07-GRN1-01, 520-540 nm) was used as the excitation source, being directed to the sample in 2 mm cuvettes (all preparation as above) through a liquid light guide. Light pulses were generated via a MOSFET transistor (STMicroelectronics STF8NM50N) and the gate was modulated by the DAQ card (National Instruments, USB-6361) controlling light-dark sequences. The measurement cycles were: dark (5 s), illuminated (5 s), and dark again (50 s). All data were sampled without previous amplification using the DAQ card. Excitation intensities were measured with a digital power meter (Thorlabs PM100), using a silicon photodiode power sensor (Thorlabs S120UV), and kept at 4 mW/cm^2.

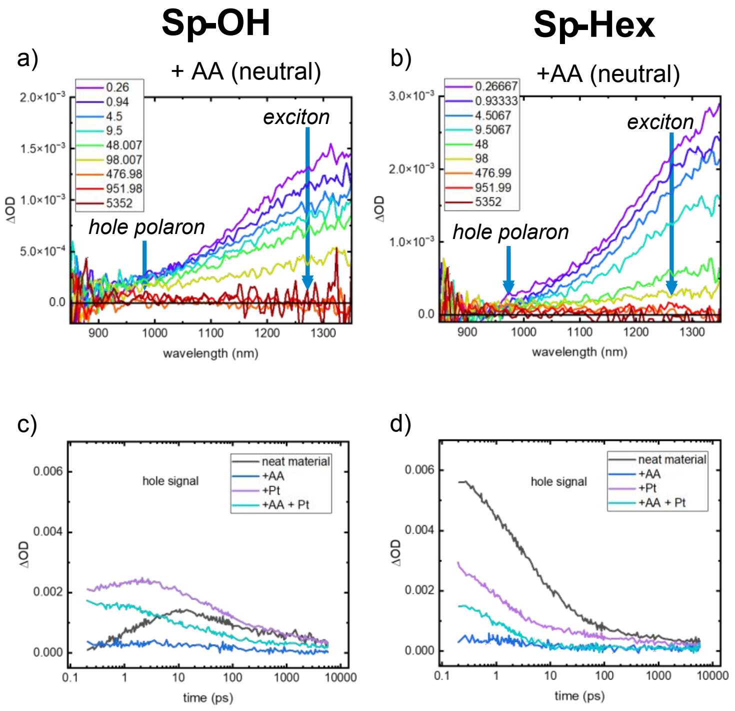

Figure S9. fs-ps transient absorption spectroscopy with ascorbic acid only, and the trace of the hole signals in all cases studied, for **Sp-OH** and **Sp-Hex**, supplementing the data shown in Figure 6.

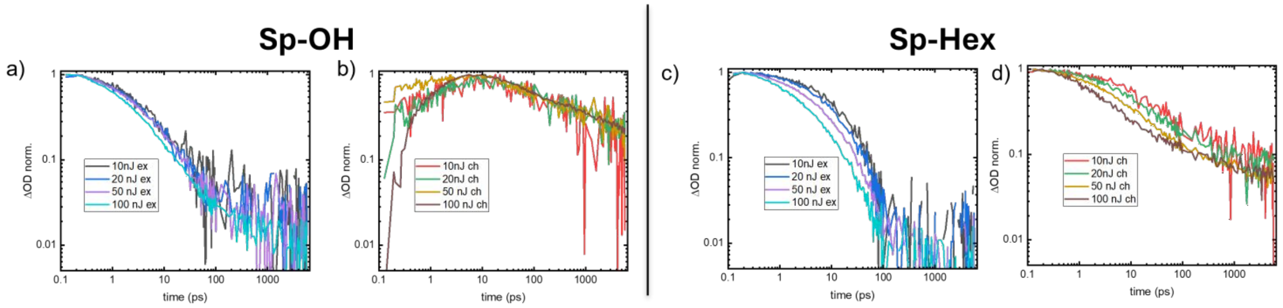

Figure S10. Normalized exciton and charge signal traces after deconvolution, as function of excitation pulse energy, for **Sp-OH** (a,b) and **Sp-Hex** (c,d). Up to 50 nJ, the signal intensity is sufficient for good signal deconvolution of exciton and charge, while no or only minor effects on the exciton behaviour are observed, excluding significant bimolecular recombination effects that otherwise could affect the data analysis.

Photo-induced absorption spectroscopy (PIAS) measurements:

In Figure S11, the charge signal peaks at 950nm and is hence in agreement to the charge signal attributed in fs-ns TAS. For **Sp-OH**, the charge signal is more pronounced, also in presence of Pt, indicating partially charge selective behavior of Pt only. The presence of ascorbic acid quenches the charge signal, but charges stay present more strongly in **Sp-OH** than in **Sp-Hex**. The overall increased background in **Sp-OH** with ascorbic acid indicates a faster reaction with it, with the background possibly being created by ascorbic acid oxidation products adsorbing to the material.

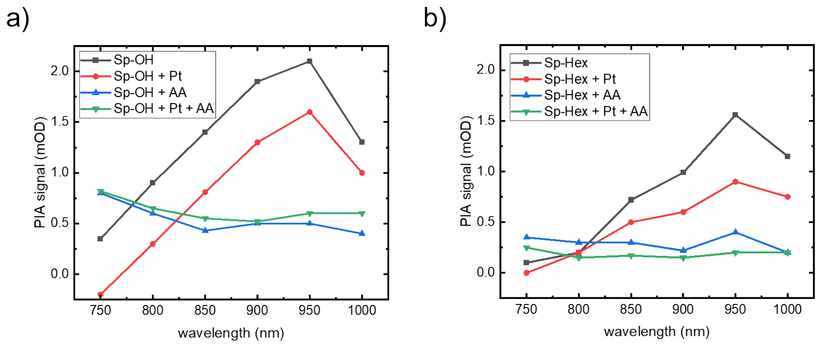

Figure S11. Spectral distribution of the PIA signal after 5s illumination in ambient conditions (electrolyte as in photocatalytic experiments), showing for a) **Sp-OH** (with Pt, ascorbic acid, and Pt and ascorbic acid) and b) **Sp-Hex** (with Pt, ascorbic acid, and Pt and ascorbic acid) including the contributions of the respective environments.

Figure S12 shows the significantly shortened decay of the hole signal in the **Sp-Hex** after 5s of illumination in presence of Pt indicates that Pt is more recombination active for holes in **Sp-Hex** than in **Sp-OH**, akin to the fs-ps TAS data discussed in the main text.

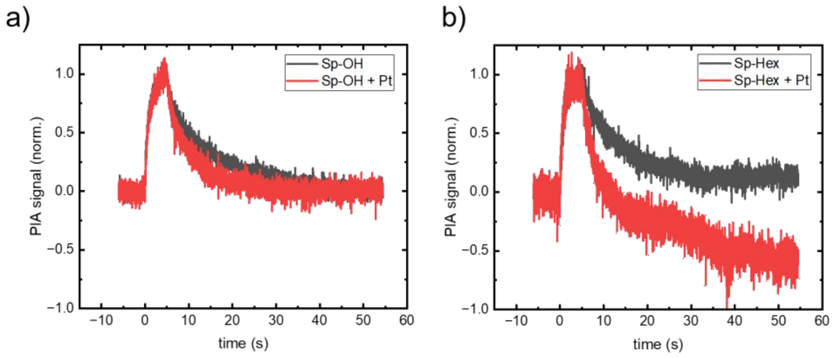

Figure S12. Normalized PIAS signal during and after illumination (5s, 530 nm diode, 4mW/cm²) of Sp-OH and Sp-OH & Pt (a), and **Sp-Hex** and **Sp-Hex** & Pt (b) at 950 nm, where the maximum of the charge signal is observed (Fig. S11).

8. Redox Potential Estimates for Proton Reduction and Ascorbic Acid Oxidation

The aqueous testing medium was made by sonicating 5 mg polymer in 10 mL NMP and adding this to a pre-dissolved solution of 1.23 g ascorbic acid, 1.6 mL 4 M NaOH and 39 μL 0.8 wt% H_2PtCl_6 solution. The pH of this aqueous solution was measured to be 6.43 using a pH meter. Using the Nernst equation, this makes the proton reduction reaction's redox potential equal to -0.38 V (SHE), equating to -4.06 eV when converted to the equivalent energy vs vacuum, using the conversion: [potential V SHE = − 4.44 - [energy eV vs vacuum], and [energy eV vs vacuum] = -4.44 – [potential V SHE].[1,2] For ascorbic acid oxidation, taking its two electron and two proton oxidation to form dehydroascorbic acid to have a redox potential +0.25 V (SHE) at of pH 2.6,[3] applying the Nernst equation puts the redox potential at 6.43 to be equivalent to -4.46 eV when converted to an absolute energy relative to vaccuum, assuming Nernstian beviour.

9. Sample Preparation for Laser Diffraction Analysis to Determine Particle Size Distributions

Polymer dispersions were prepared identically to how they were measured for photocatalysis experiments, except H_2PtCl_6 was omitted. The particle size distributions were measured using a Malvern Mastersizer 2000. This involved measuring a background with deionised water, then adding the 35 mL photocatalyst particle dispersion, sonicating briefly and circulating the dispersion around the instrument for 30 s, then 10 laser diffraction measurements were made, with brief sonication between measurements. The mean of these 10 measurements was taken to obtain the particle size distributions and D[3,2] values calculated, where D[3,2] is defined by the equation below, where n_i is the number of particles in the distribution of diameter d_i. D[3,2] is a geometric mean, where the total surface area of the particles in the distribution is calculated, and the diameter is given if there were the same number of uniform particles of the same diameter. This gives the external surface area normalised mean diameter of the particles in the sample and is most appropriate for catalytic applications where the external surface area is important.

$$D[3,2] = \frac{\sum_{i=1}^{n} n_i d_i^3}{\sum_{i=1}^{n} n_i d_i^2}$$

10. Inductively Coupled Plasma Mass Spectrometry Residual Pd Measurements (ICP-MS)

Between 2.5 to 3.5 mg polymer was digested in 2 mL nitric acid using an Anton Paar Microwave Digestive System Multiwave GO Plus for 2 h. The digested polymer acidic solution was diluted to a total volume of 100 mL with deionised water. Inductively coupled plasma mass spectrometry was conducted using a Shimadzu ICPMS-2030 with 5 calibration solutions between 0 and 500 ppb palladium. The results for the polymers are displayed below.

Polymer	Pd /ppm
Sp-Si	0.43
Sp-Hex	2.42
Bu-Si	1.03
Sp-Si	0.49

Table S3. Pd levels detected in ppm for polymer samples

11. Isotherm Measurements

All gases for sorption analysis were supplied by BOC at a purity of ≥99.999%. The polymers were precipitated into methanol following Soxhlet purification (or washed if they were insoluble) as described in the polymer synthesis section, then dried in a vacuum oven at 50 °C for overnight. For N_2 sorption measurements, between 30 to 100 mg polymer was loaded into a Micromeritics 3Flex gas sorption analyser, where they were heated at 100 °C for 4 h under vacuum in situ before beginning the measurement to thoroughly dry the polymers. The N_2 sorption isotherms were measured in a liquid N_2 dewar. BET surface areas were calculated from the linear regions of the BET plots between relative pressures of 0.01 and 0.03. For water sorption, 30 to 100 mg polymer was loaded into an Anton Paar Autosorb iQ-XR gas sorption analyser, where they were heated at 110 °C for 8 h under

vacuum on the apparatus before beginning the measurement to thoroughly dry the polymers. The water sorption isotherm was measured at 25 °C in a water bath.

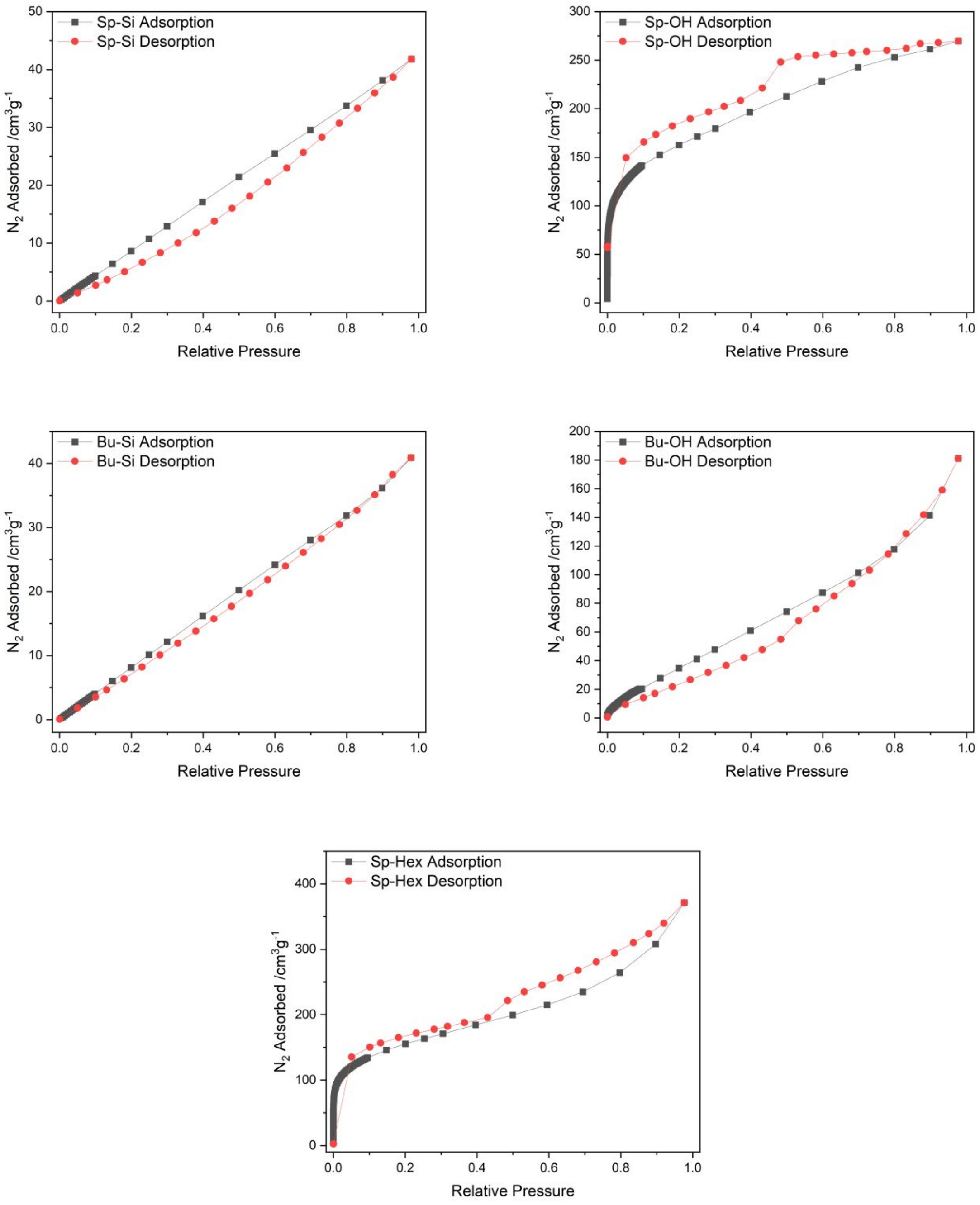

Figure S13. Full N_2 sorption isotherms for **Sp-Si**, **Sp-OH**, **Sp-Hex**, **Bu-Si** and **Bu-OH**

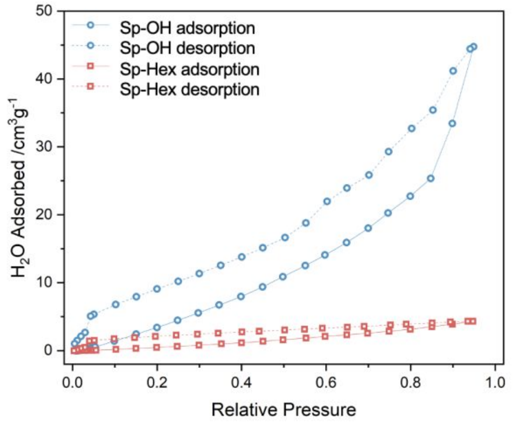

Figure S14. Full H_2O sorption isotherms for **Sp-OH** and **Sp-Hex.**

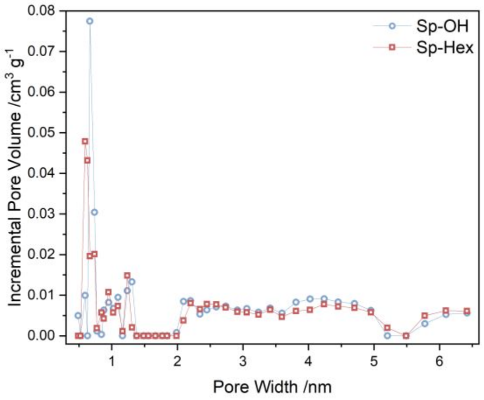

Figure S15. Estimated pore size distributions for porous polymers **Sp-OH** and **Sp-Hex**, calculated from the N_2 sorption isotherms using N2-Tarazona NLDFT.

12. Synthesis and Compound Characterization

All reagents and solvents were purchased from Merck, Fluorochem or Alfa Aesar and used without further purification. Unless otherwise stated, ^{1}H and ^{13}C NMR spectra were recorded at ambient temperature on a 400 MHz Bruker spectrometer at 400 MHz and referenced against the residual signal of the solvent. All NMR data was analysed using MestReNova software. Gel permeation chromatography (GPC) was performed on soluble polymers dissolved in chlorobenzene at 1 mg mL^{-1} using Agilent 1260 Infinity II.

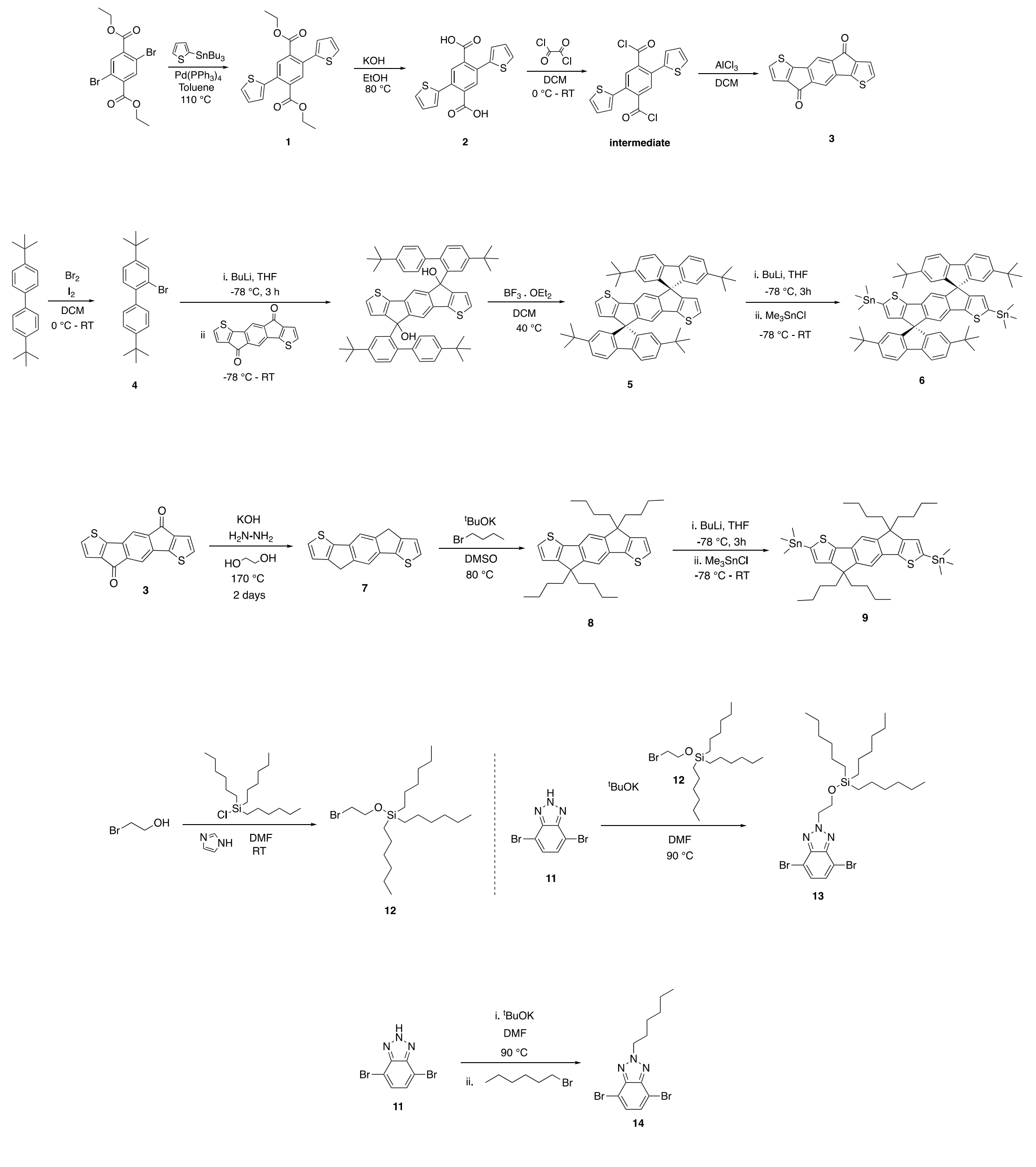

Scheme S1. Synthetic pathways for the synthesis of monomers 6, 9, 13 and 14 used to synthesise all polymers

1

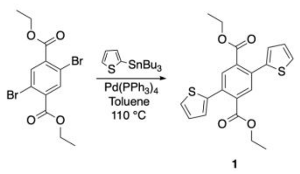

Scheme S2. Synthesis of **1**

The synthesis of **1** was adapted from a previously reported procedure.[4] 1,4-Diethyl 2,5-dibromo-1,4-benzenedicarboxylate (11.49 g, 30.2 mmol, 1 eq) and tetrakis(triphenylphosphine) (0.92 g, 0.8 mmol, 0.03 eq) were added to a 2-neck round bottom flask and purged with N_2. 2-(tributylstannyl)thiophene (24 mL, 75.6 mmol, 2.5 eq) was added followed by anhydrous toluene (60 mL). The solution was refluxed at 110 °C overnight. The next day after the reaction was complete, the solvent was removed under reduced pressure and the crude mixture was passed through a silica plug wetted with petroleum ether: ethyl acetate (80:20), using more eluent to wash it all through. The filtrate solvent was removed under reduced pressure to give a yellow solid. This was recrystallised in hexane: dichloromethane to produce large colourless crystals **1** (9.73 g, 83%). ^{1}H NMR (400 MHz, $CDCl_3$) δ 7.81 (s, 2H), 7.39 (dd, J = 4.9, 1.4 Hz, 4H), 7.10-7.06 (m, 4 H), 4.22 (q, J = 7.1 Hz, 4H), 1.15 (t, J = 7.1 Hz, 6H). ^{13}C NMR (400 MHz, $CDCl_3$) δ 167.8, 140.7, 134.2, 133.60, 132.0, 127.5, 127.1, 126.6, 61.8, 14.0.

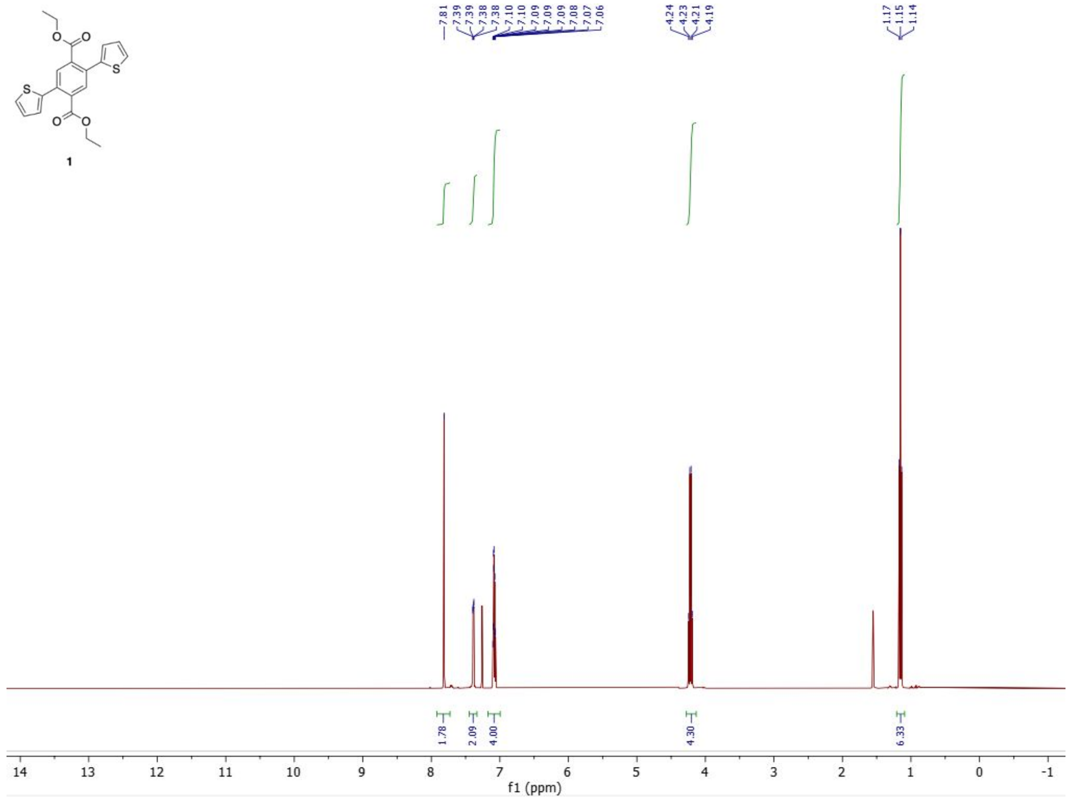

Figure S16. ^{1}H NMR of **1**, 400 MHz, $CDCl_3$

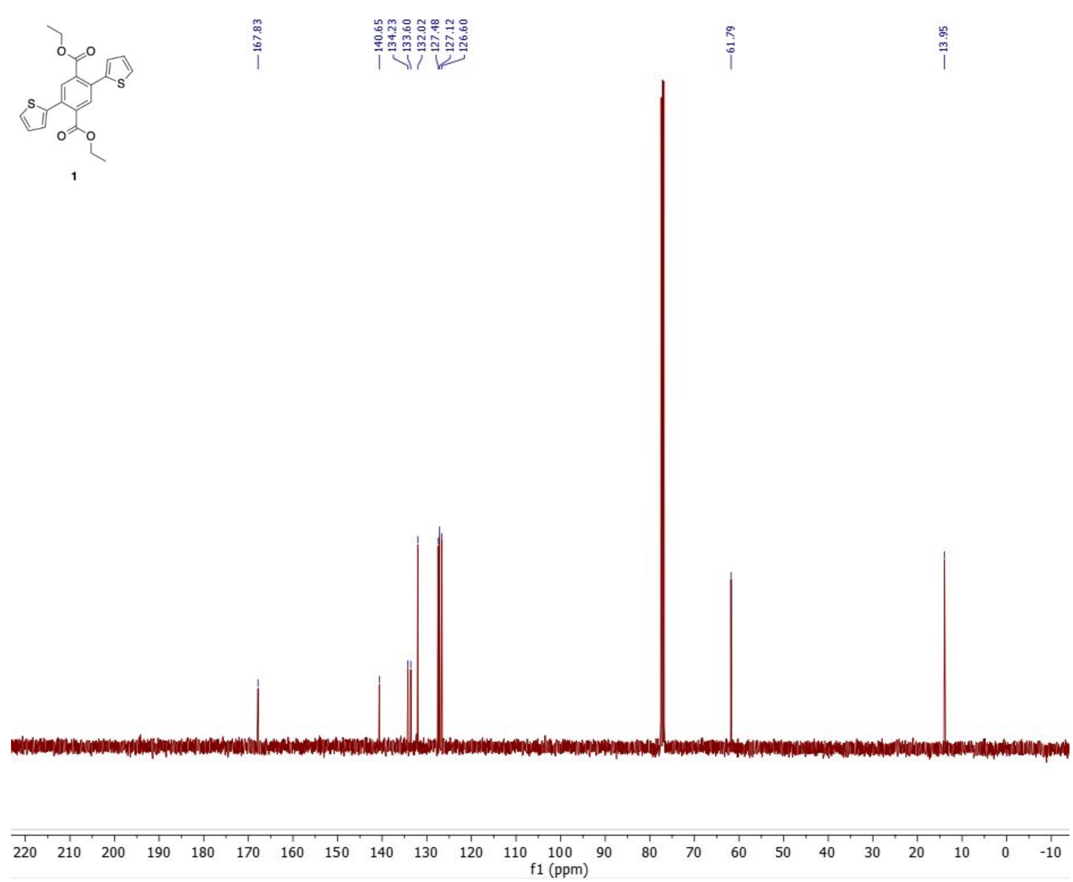

Figure S17. ^{13}C NMR of **1**, 400 MHz, $CDCl_3$

2

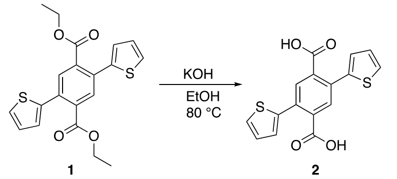

Scheme S3. Synthesis of **2**

The synthesis of **2** was adapted from a previously reported procedure.[5] **1** (9.66 g, 25.0 mmol, 1 eq) was stirred in ethanol (300 mL) and KOH (14.00 g, 249.9 mmol, 10 eq) was added. The solution was refluxed at 80 °C overnight. The next day the product had slightly precipitated. The solution was cooled, poured onto ice, and neutralised with HCl solution whereupon it precipitated fully. The precipitate was collected by vacuum filtration washing thoroughly with deionised water followed by methanol. The solid was collected and dried in a vacuum oven overnight to give a white powder **2** (6.81 g, 80%). ^{1}H NMR (400 MHz, $(CD_3)_2SO$) δ 13.43 (s, 2H), 7.70 (s, 2H), 7.67 (dd, $J = 5.0$, 1.2 Hz, 2H), 7.25 (dd $J = 3.6$, 1.2 Hz, 2H), 7.15 (dd, $J = 5.0$, 3.6 Hz, 2H). ^{13}C NMR (400 MHz, $(CD_3)_2SO$) δ 168.7, 139.7, 134.5, 131.4, 130.2, 127.9, 127.5, 127.1.

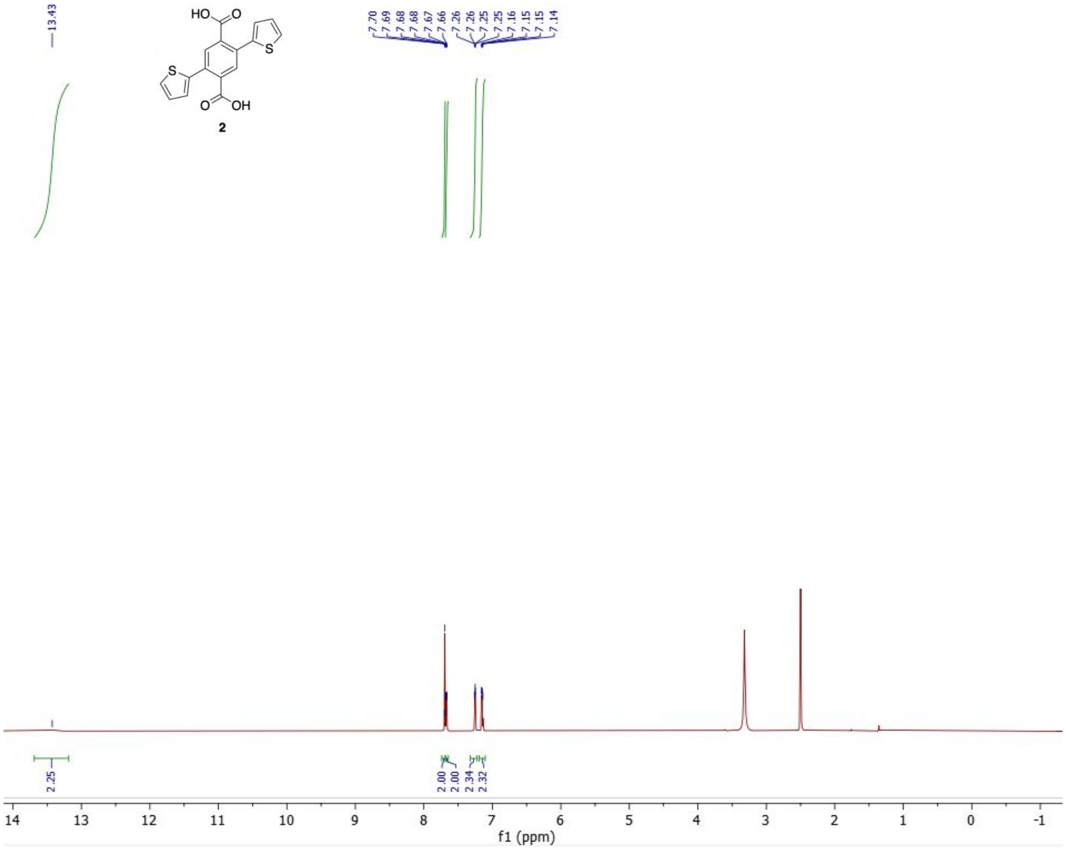

Figure S18. ^{1}H NMR of **2**, 400 MHz, $(CD_3)_2SO$

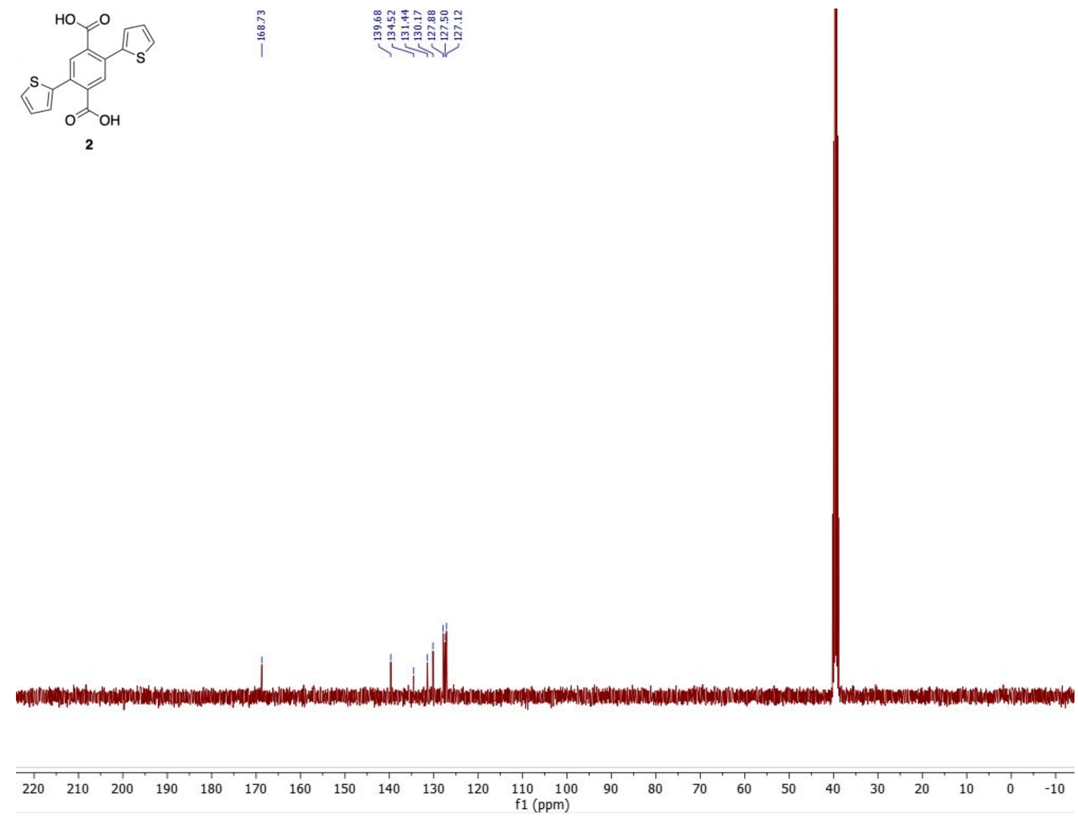

Figure S19. ^{13}C NMR of **2** 400 MHz, $(CD_3)_2SO$

3

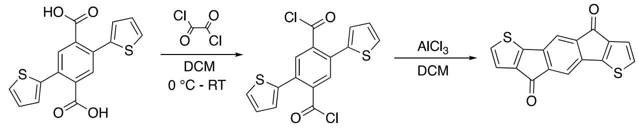

2 intermediate 3

Scheme S4. Synthesis of **3**

The synthesis of **3** was adapted from a previously reported procedure.[5] **2** (6.11g, 18.5 mmol, 1 eq), was added to a 2-neck round bottom flask and put under high vacuum attached to a Schlenk line while being gently heated with a heat gun for several minutes, then purged with N_2. Anhydrous dichloromethane (300 mL) was added, and the suspension was stirred at room temperature under N_2. Oxalyl chloride (6.4 mL, 74.0 mmol, 4 eq) was added, followed by anhydrous dimethylformamide (2 mL) *dropwise*, to keep the rate of gas evolution manageable. The suspension, which became a solution and turned slightly yellow, was stirred at room temperature for 3 h under N_2, after which time the solvent and excess oxalyl chloride was removed under reduced pressure. The resultant yellowish solid intermediate was immediately purged with N_2 and dissolved in anhydrous dichloromethane (200 mL). Aluminium trichloride (9.87 g, 74.0 mmol, 4 eq) was added through the side neck of the round bottom flask under a positive N_2 pressure and it was stirred at room temperature overnight under N_2. The next day it was poured into 2 M HCl in ice, then the solid precipitate underwent vacuum filtration, washing with deionised water, methanol, acetone, dichloromethane and diethyl ether. The solid was dried in a vacuum oven overnight to give blue powder **3** (5.06 g, 93%). ^{1}H NMR (400 MHz, $(CD_3)_2SO$) δ 7.59 (d, *J* = 4.9 Hz, 2H), 7.52 (s, 2H), 7.19 (d, *J* = 4.9 Hz, 2 H). This was too insoluble to obtain ^{13}C NMR.

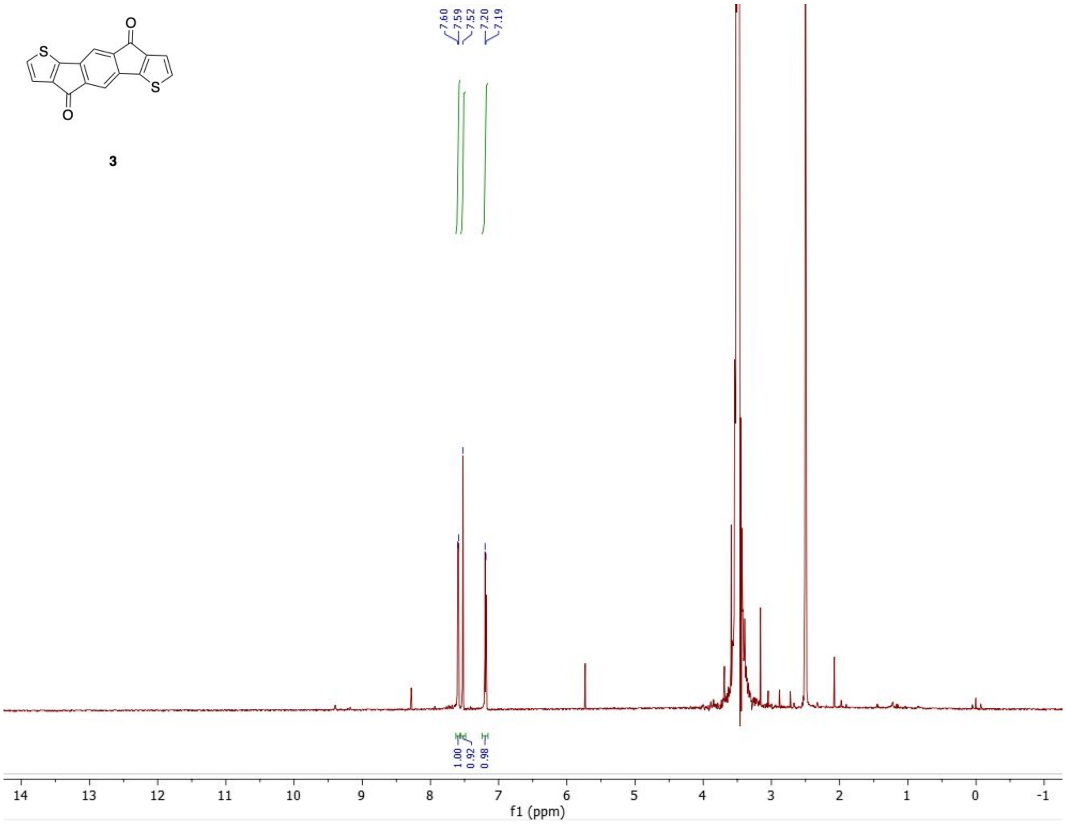

Figure S20. ^{1}H NMR of **3**, 400 MHz, $(CD_3)_2SO$

4

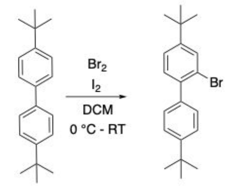

4

Scheme S5. Synthesis of **4**

The synthesis of **4** was adapted from a previously reported procedure.[6] 4,4′-Bis(1,1-dimethylethyl)-1,1′-biphenyl (31.63 g, 139.7 mmol, 1 eq) and I_2 (1.21 g, 4.7 mmol 0.03 eq) were dissolved in dichloromethane and cooled to 0 °C in a 2-neck round bottom flask fitted with a bubbler containing Br_2 quencher (1M Na_2SO_4 and 1 M NaOH solution). Br_2 (9.52 mL, 150.9 mmol, 1.08 eq) was added and it was stirred at room temperature for 2 days, monitoring the reaction by crude NMR. After complete reaction, water and Br_2 quencher were added and it was transferred to a separating funnel. The organic phase was collected and washed with water, brine and Na_2SO_4 was added to the organic phase. The dried organic phase was filtered, then the solvent was removed under reduced pressure to give a colourless solid. This was recrystallised in hexane and the colourless crystals were collected by vacuum filtration. The recrystallisation filtrate was kept and the solvent removed under reduced pressure. A second recrystallisation was conducted with this, and the two batches were combined to give colourless crystals **4** (41.5 g, 86%). ^1H NMR (400 MHz, $CDCl_3$) δ 7.66 (d, $J = 2.0$ Hz, 1H), 7.45-7.42 (m, 2H), 7.37-7.34 (m, 3H) 7.26 (d, $J = 8.0$ Hz, 1 H), 1.37 (s, 9H), 1.35 (s, 9H). ^{13}C NMR (400 MHz, $CDCl_3$) δ 152.0, 150.2, 139.5, 138.0, 131.0, 130.2, 129.1, 124.8, 124.5, 122.5, 34.6, 31.4, 31.3.

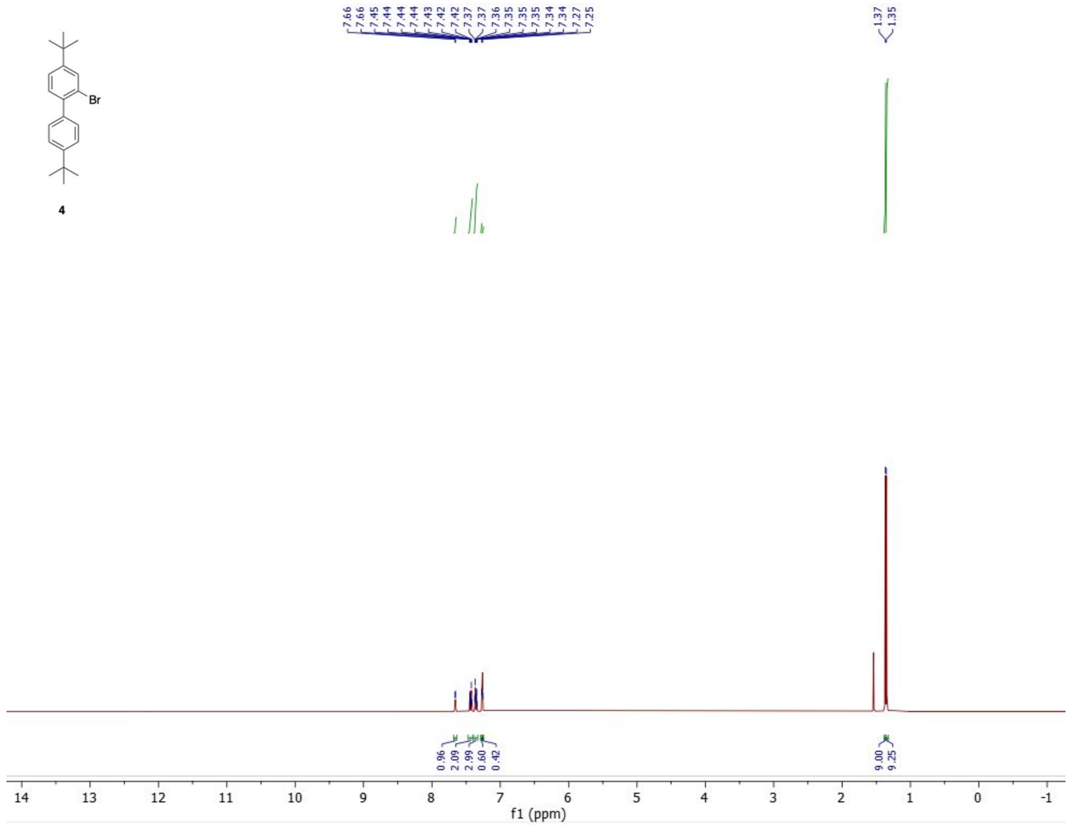

Figure S21. ^1H NMR of **4**, 400 MHz, $CDCl_3$

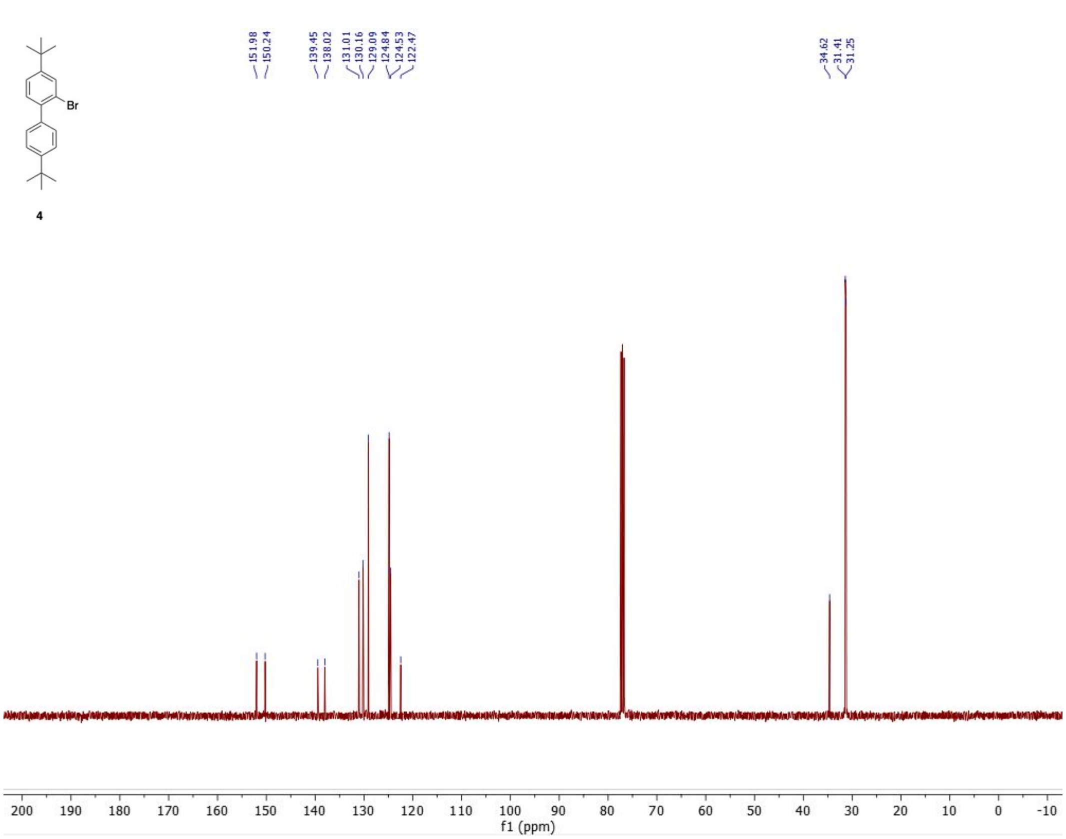

Figure S22. ^{13}C NMR of **4**, 400 MHz, $CDCl_3$

5

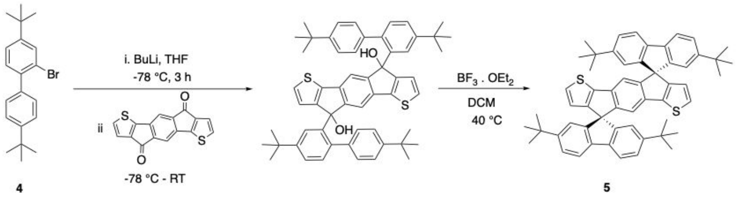

Scheme S6. Synthesis of **5**

The synthesis of **5** was adapted from previously reported procedures.[4,7] **4** (17.60 g, 51.0 mmol, 5 eq) was added to a 2-neck round bottom flask and put under high vacuum via a Schlenk line for 30 minutes, then purged with N_2. It was dissolved in anhydrous tetrahydrofuran (300 mL) and cooled to -78 °C. n-butyllithium (31.9 mL, 1.6 M, 5 eq) was added slowly, whereupon the solution turned yellow and solid began to precipitate. This was left stirring at -78 °C for 3 h under N_2. Finely divided and vacuum oven dried 4 (3.00 g, 10.2 mmol, 1 eq) was quickly added through the side neck under a positive pressure of N_2. This was left stirring overnight under N_2 and allowed to reach room temperature. The tetrahydrofuran was removed under reduced pressure, then the alcohol intermediate was extracted with dichloromethane in a separating funnel, washing with water, brine and Na_2SO_4 was added to the organic phase. This underwent gravity filtration and was transferred to a round bottom flask, with more dichloromethane added such that the total volume was about 200 mL. This was stirred vigorously and boron trifluoride etherate (1 mL) was added dropwise. The solution was refluxed for 2 h. After complete reaction, everything was poured through a short silica plug eluting with dichloromethane to remove most of the tar-like impurities. The filtrate was collected, and the solvent was removed under reduced pressure, then purified by dry loading on a silica column, eluting with a graduated eluent from pure petroleum ether to petroleum ether: dichloromethane (90:10). The purified material was subsequently collected, dissolved in hot chloroform (20 mL) and pipetted into stirring methanol to precipitate it. This was then collected by vacuum filtration, washed with methanol and vacuum oven dried to recover a white solid **5** (1.89 g, 23%). ^1H NMR (400 MHz, $CDCl_3$) δ 7.75 (dd, *J* = 8.1, 0.6 Hz, 4H), 7.42 (dd, *J* = 8.1, 1.8 Hz, 4 H), 7.08 (d, *J* = 4.9 Hz, 2H), 6.79 (s, 2H), 6.76 (dd, *J* = 1.6, 0.6 Hz, 4 H), 6.41 (d, *J* = 4.9 Hz, 2H), 1.19 (s, 36 H). ^{13}C NMR (400 MHz, $CDCl_3$) δ 152.7, 152.56, 150.9, 147.21, 143.5, 139.4, 136.6, 127.3, 125.1, 121.7, 120.7, 119.3, 114.7, 63.9, 35.0, 31.6.

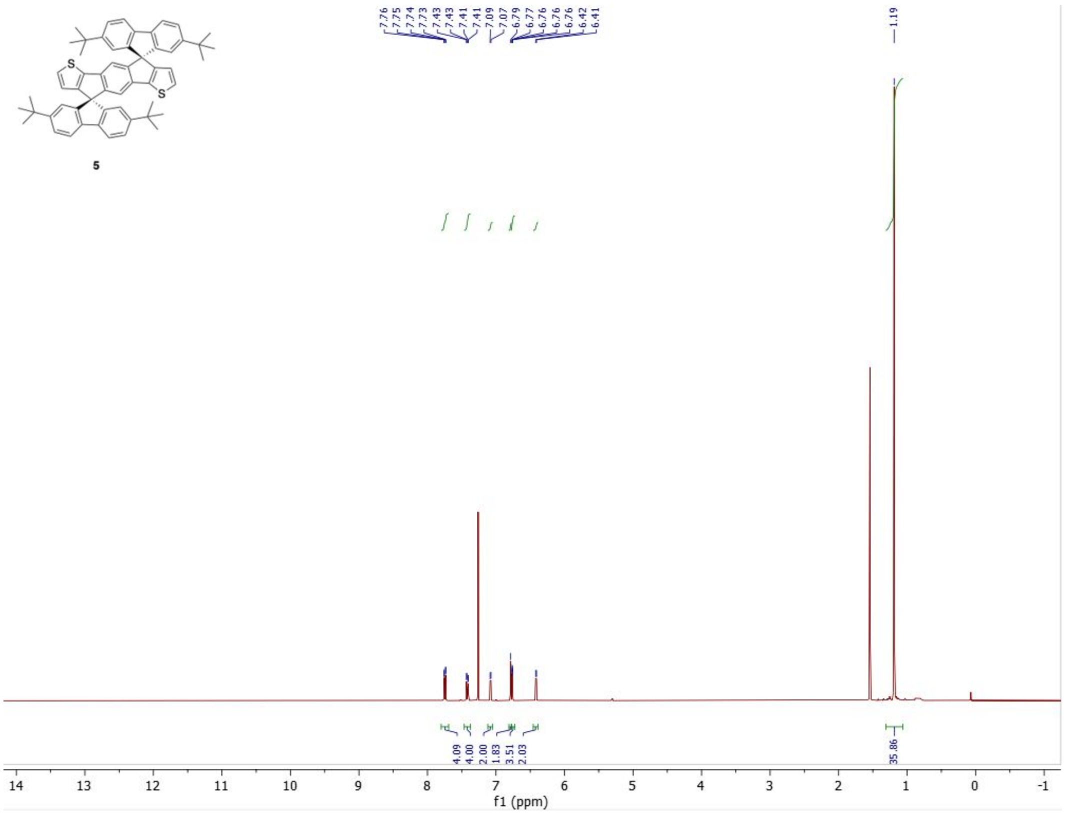

Figure S23. ^1H NMR of **5**, 400 MHz, $CDCl_3$

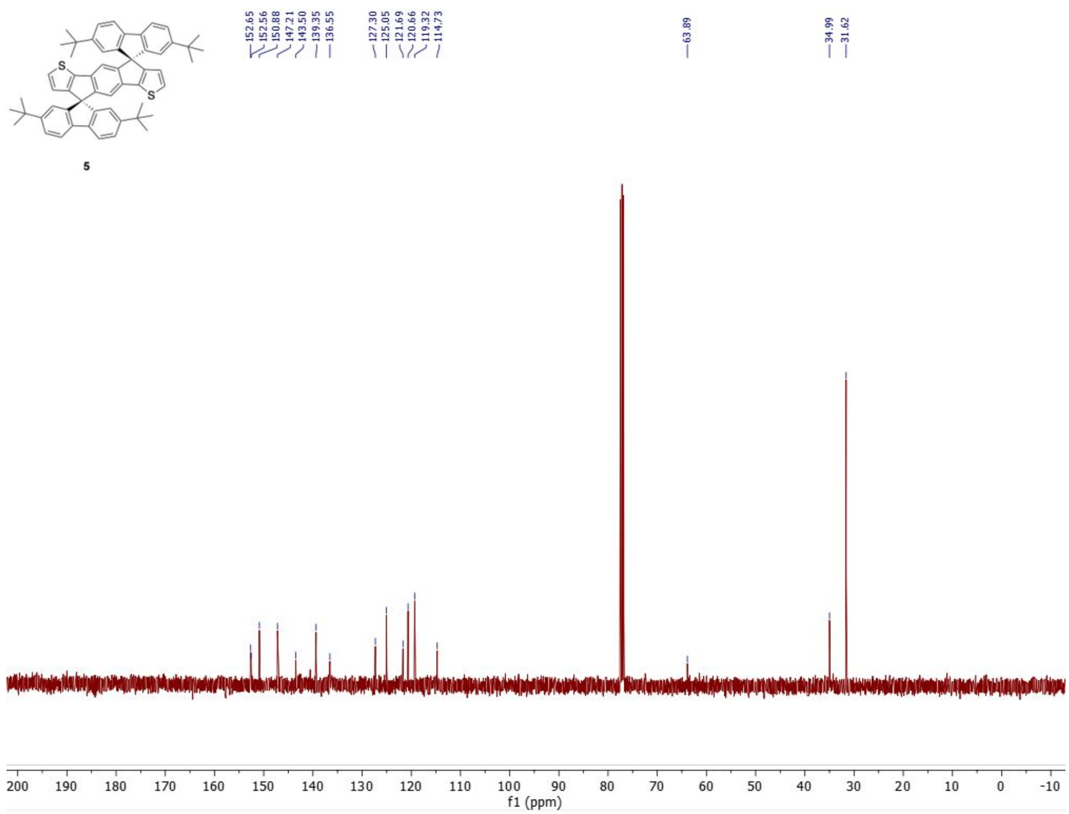

Figure S24. ^{13}C NMR of **5**, 400 MHz, $CDCl_3$

6

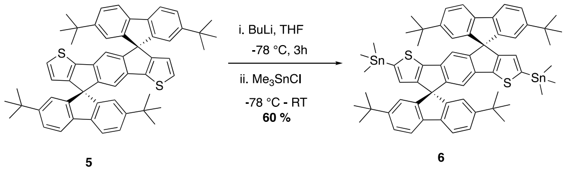

Scheme S7. Synthesis of **6**

5 (947 mg, 1.20 mmol, 1 eq) was assed to a 2-neck round bottom flask, purged with N_2 and dissolved by adding anhydrous tetrahydrofuran (100 mL). This was cooled to -78 °C, n-butyllithium (1.9 mL, 1.6 M, 2.5 eq) was added dropwise and it was stirred under N_2 for 3 h. Trimethyltin chloride (3.6, 1M, 3 eq) was added and it was allowed to stir under N_2 reaching room temperature overnight. The next day the product was extracted with dichloromethane in a separating funnel, washing twice with water, brine and Na_2SO_4 was added to the organic phase. Gravity filtration was undertaken, and the solvent from the filtrate was removed under reduced pressure. The off-white solid was recrystallised in chloroform: isopropanol overnight to recover a white crystalline solid **6** (670 mg, 60%). ^{1}H NMR (400 MHz, $CDCl_3$) δ 7.75 (d, $J = 8.0$ Hz, 4H), 7.42 (dd, $J = 8.0$, 1.8 Hz, 4H), 6.77 (d, $J = 1.8$ Hz, 4H), 6.74 (s, 2H), 6.46 (s, 2H), 1.19 (s, 36H), 0.22 (s, 18 H). ^{13}C NMR (400 MHz, $CDCl_3$) δ 154.4, 152.8, 150.8, 149.5, 147.7, 140.8, 139.3, 136.3, 129.4, 124.9, 120.8, 119.2, 114.9, 63.4, 35.0, 31.6, -8.1. MS (MALDI-TOF): MS calculated for $[C_{62}H_{70}S_2Sn_2]^+$: m/z = 1116.30; measured m/z = 1116.46

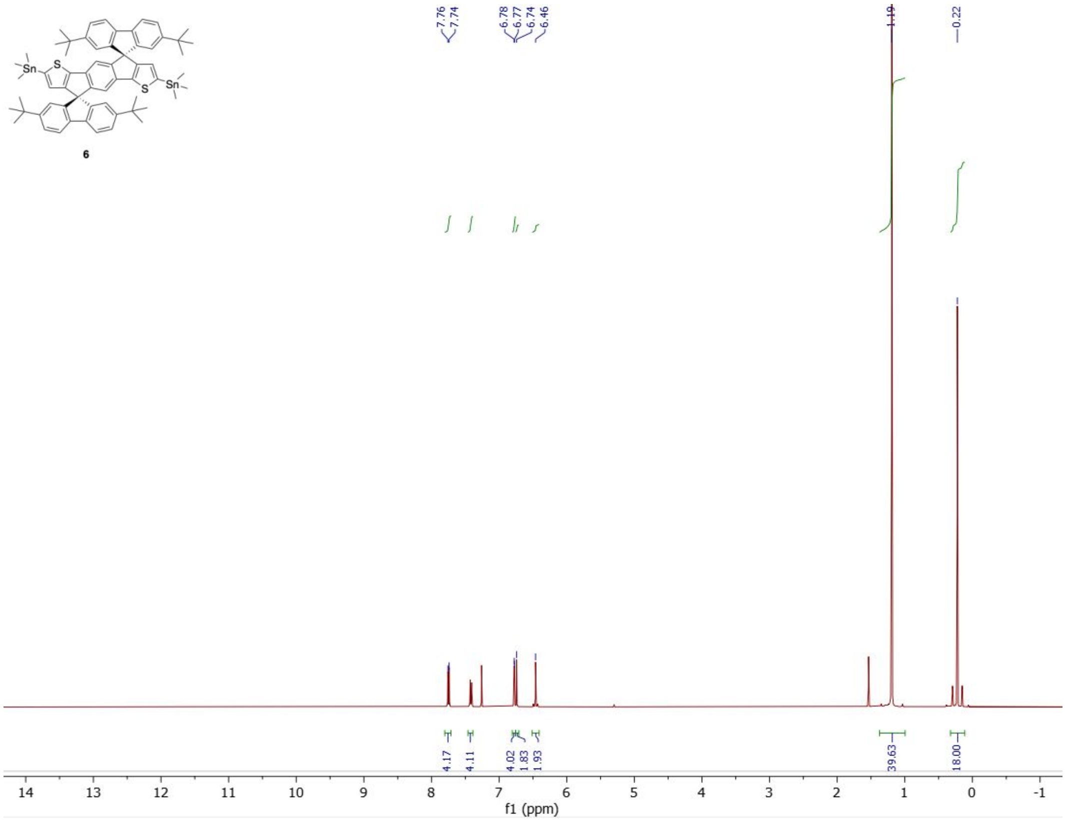

Figure S25. ^{1}H NMR of **6**, 400 MHz, $CDCl_3$

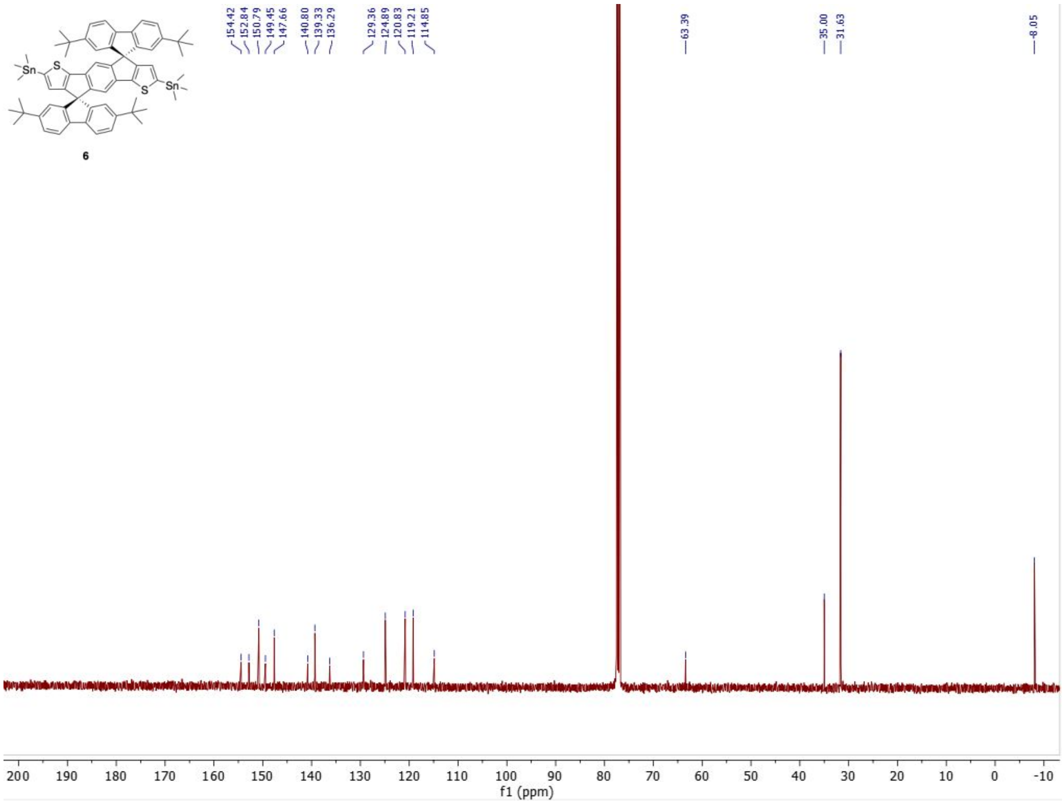

Figure S26. ^{13}C NMR of **6**, 400 MHz, $CDCl_3$

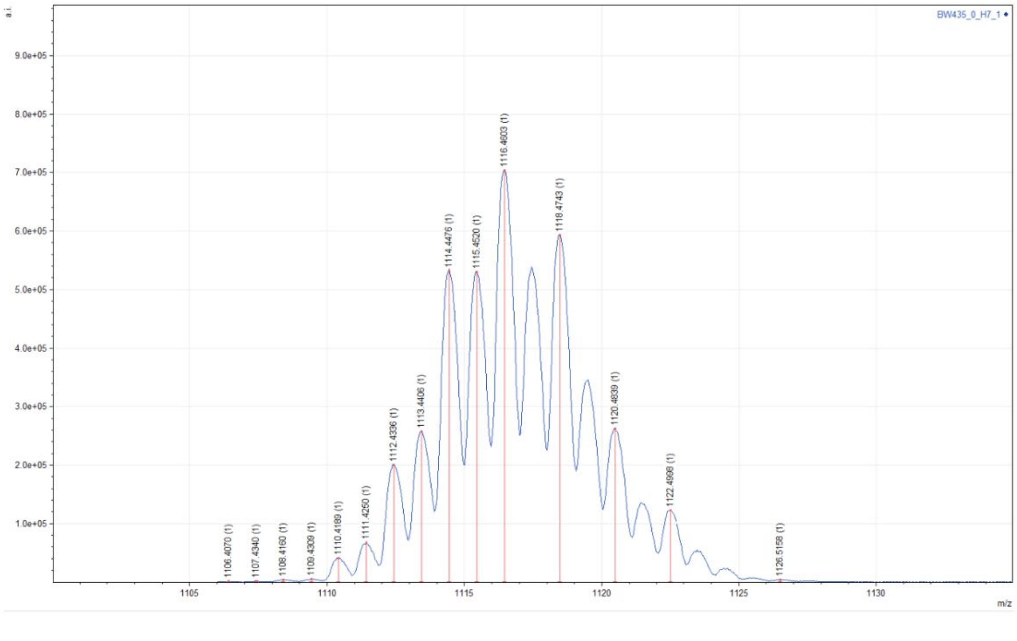

Figure S27. MS (MALDI-TOF) of **6**

7

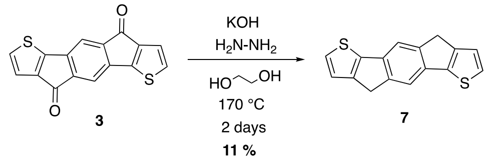

Scheme S8. Synthesis of **7**

The synthesis of **7** was adapted from a previously reported procedure.[5] **3** (4.70 g, 16.0 mmol, 1 eq), KOH (17.9 g, 319.4 mmol, 20 eq) and diethylene glycol (400 mL) were purged with N_2 for 20 minutes, then hydrazine monohydrate (8.0 mL, 20 eq) was added, and it was heated to 150 °C with a condenser fitted for 2 days. This was cooled then added to ice and 2 M HCl then underwent vacuum filtration to recover a dark brown solid. This was washed with water and acetone then collected by dissolving in chloroform. The solid was purified on a silica plug, eluting with dichloromethane: petroleum ether (70:30) to recover a light brown solid **7** (451 mg, 11 %). ^1H NMR (400 MHz, $CDCl_3$) δ 7.62 (s, 1H), 7.29 (d, $J = 4.8$ Hz, 1H), 7.12 (d, $J = 4.8$ Hz, 1H), 3.75 (s, 2H). ^{13}C NMR (400 MHz, $CDCl_3$) δ 147.1, 145.6, 143.6, 136.3, 126.5, 123.0, 116.0, 34.1.

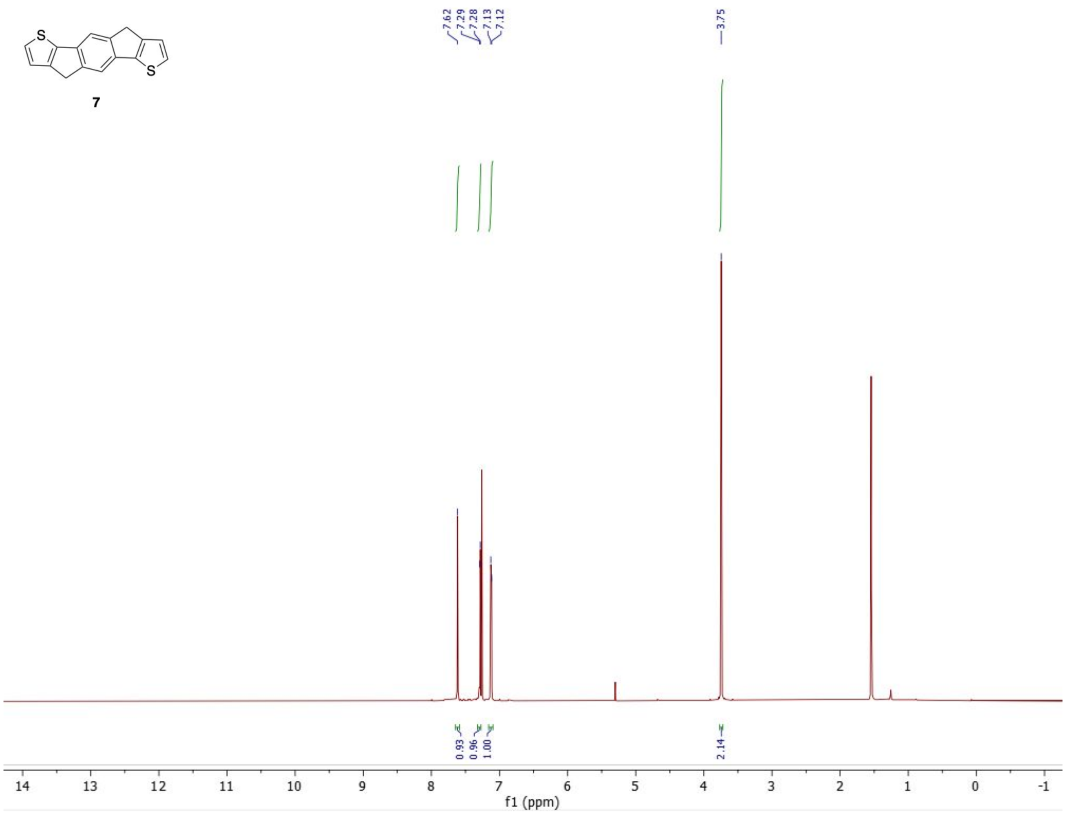

Figure S28. ^1H NMR of **7**, 400 MHz, $CDCl_3$

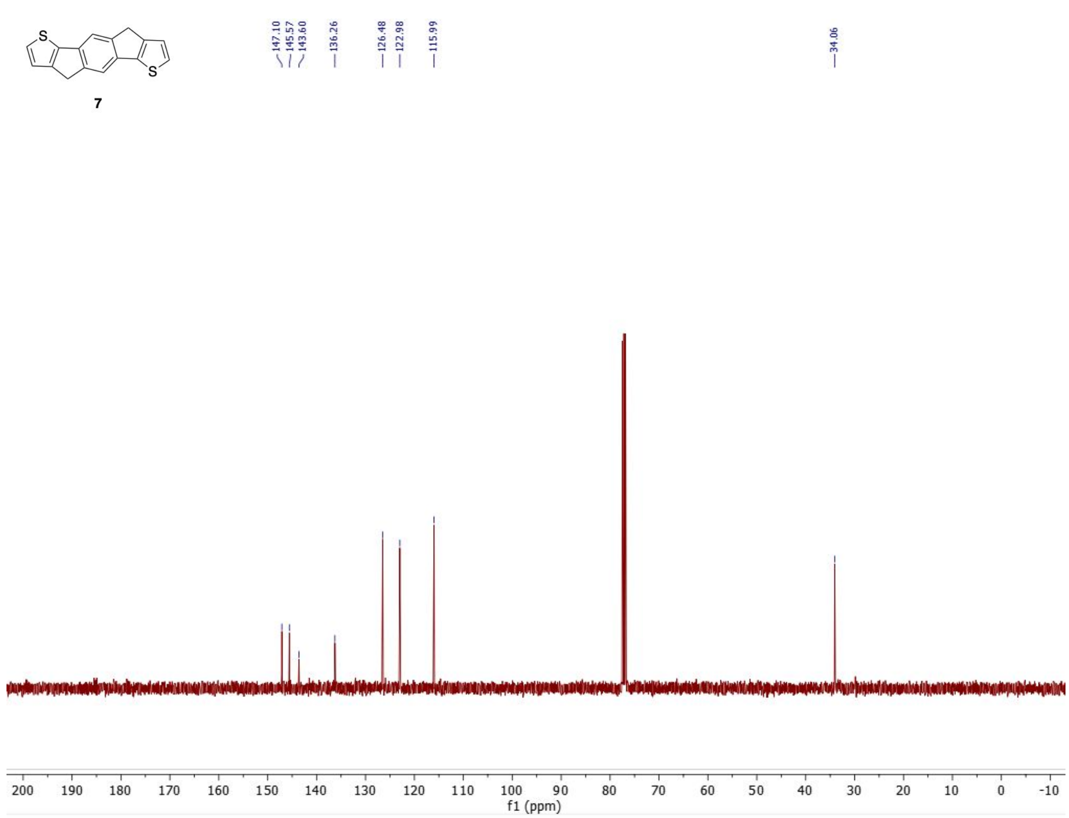

Figure S29. ^{13}C NMR of **7**, 400 MHz, $CDCl_3$

8

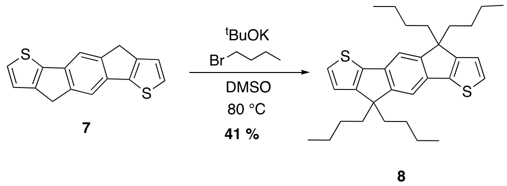

Scheme S9. Synthesis of **8**

The synthesis of **8** was adapted from a previously reported procedure of a similar compound.[5] **7** (0.24 g, 0.9 mmol, 1 eq) was added to a 2-neck round bottom flask and dried under high vacuum, then purged with N_2. Anhydrous dimethyl sulfoxide (10 mL) followed by potassium tert-butoxide (0.61, 5.4 mmol, 6 eq) portion wise while under stirring under N_2, whereupon the suspension turned red-brown. This was heated to 80 °C and stirred for 1 h, after which 1-bromobutane (0.6 mL, 5.4 mmol, 6 eq) was added dropwise and continued to stir at 80 °C under N_2 overnight. After complete reaction, it was cooled then poured into a separating funnel with water and extracted with dichloromethane, washing with water and brine, then Na_2SO_4 was added to the organic phase. This underwent gravity filtration, and the solvent was removed under reduced pressure to give a brown residue. This was purified on a silica column, eluting with petroleum ether to recover a pale brown crystalline solid. This was finally recrystallised in dichloromethane: methanol to give slightly orange-brown crystals **8** (0.19 g, 41%). ^{1}H NMR (400 MHz, CD_2Cl_2) δ 7.33 (s, 2H), 7.29 (d, $J = 4.8$ Hz, 2H), 7.00 (d, $J = 4.8$ Hz, 2H), 2.04-1.97 (m, 4H), 1.92 (m, 4H), 1.18-1.09 (m, 8H), 0.82-0.75 (m, 8H), 0.72 (t, $J = 7.4$ Hz, 12H). ^{13}C NMR (400 MHz, $CDCl_3$) δ 155.3, 153.4, 141.8, 135.7, 126.3, 121.9, 113.3, 53.7, 39.1, 26.6, 23.3, 14.1. GC-MS calculated for $[C_{32}H_{42}S_2]+$: $m/z = 490.27$; found: $m/z = 490.3$

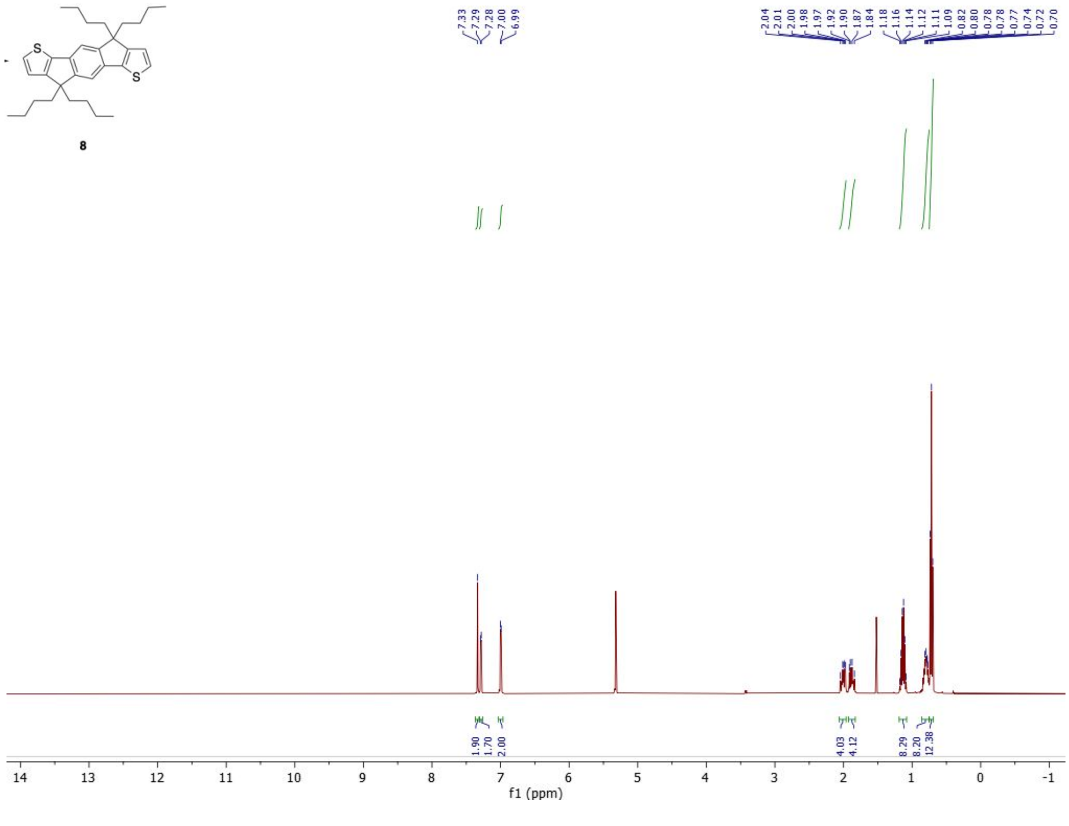

Figure S30. ^{1}H NMR of **8**, 400 MHz, CD_2Cl_2

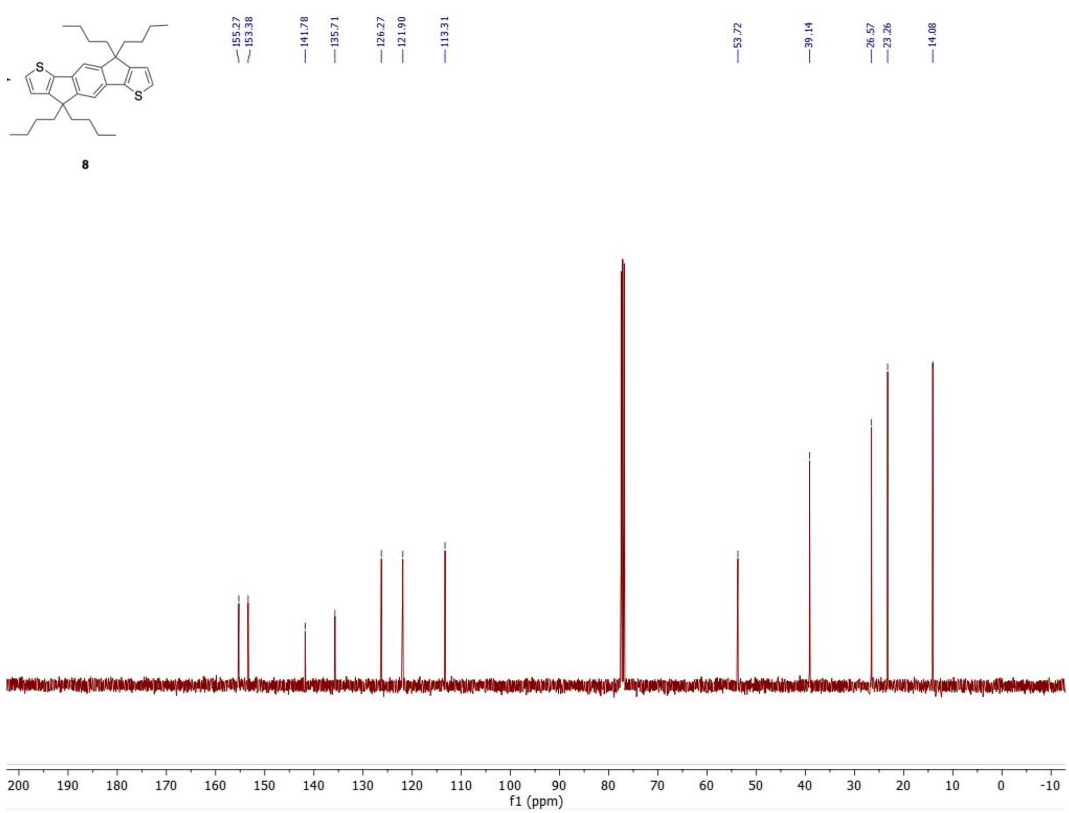

Figure S31. ^{13}C NMR of **8**, 400 MHz, $CDCl_3$

9

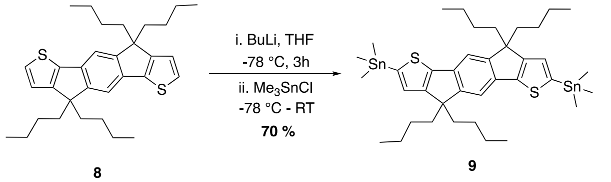

Scheme S10. Synthesis of **9**

8 (170 mg, 0.35 mmol, 1 eq) was added to a 2-neck round bottom flask and dried under high vacuum, then purged with N_2. Anhydrous tetrahydrofuran (10 mL) was added to fully dissolve the compound and it was cooled to -78 °C. n-butyl lithium (0.6 mL, 1.6 M, 2.5 eq) was added dropwise whereupon the solution turned slightly red and precipitate formed. This was continued to stir for 2 h and trimethyltin chloride (1.0 mL, 1.0 M, 3 eq) was added dropwise to quench the intermediate, whereupon the precipitate redissolved. This was stirred and allowed to reach room temperature overnight. This was poured into a separating funnel with water and extracted with dichloromethane, washing with water and brine, then Na_2SO_4 was added. This underwent gravity filtration, and the solvent was removed under reduced pressure to give a light brown solid. This was recrystallised in dichloromethane: methanol to give colourless crystals **9** (199 mg, 70 %). ^1H NMR (400 MHz, CD_2Cl_2) δ 7.31 (s, 2H), 7.03 (s, 2H), 1.94 – 2.03(m, 4H), 1.81 – 1.90 (m, 4H), 1.13 (m, 8H), 0.77 – 0.89 (m, 8H), 0.72 (t, $J = 7.3$ Hz, 12H), 0.40 (s, 18H). ^{13}C NMR (400 MHz, $CDCl_3$) δ 157.3, 153.6, 147.8, 139.5, 135.4, 129.6, 113.6, 53.1, 39.2, 26.6, 23.29, 14.1, -7.9. HRMS (ESI): HR-MS calculated for $[C_{38}H_{58}S_2Sn_2+H]^+$: m/z = 819.2097; measured m/z = 819.2126

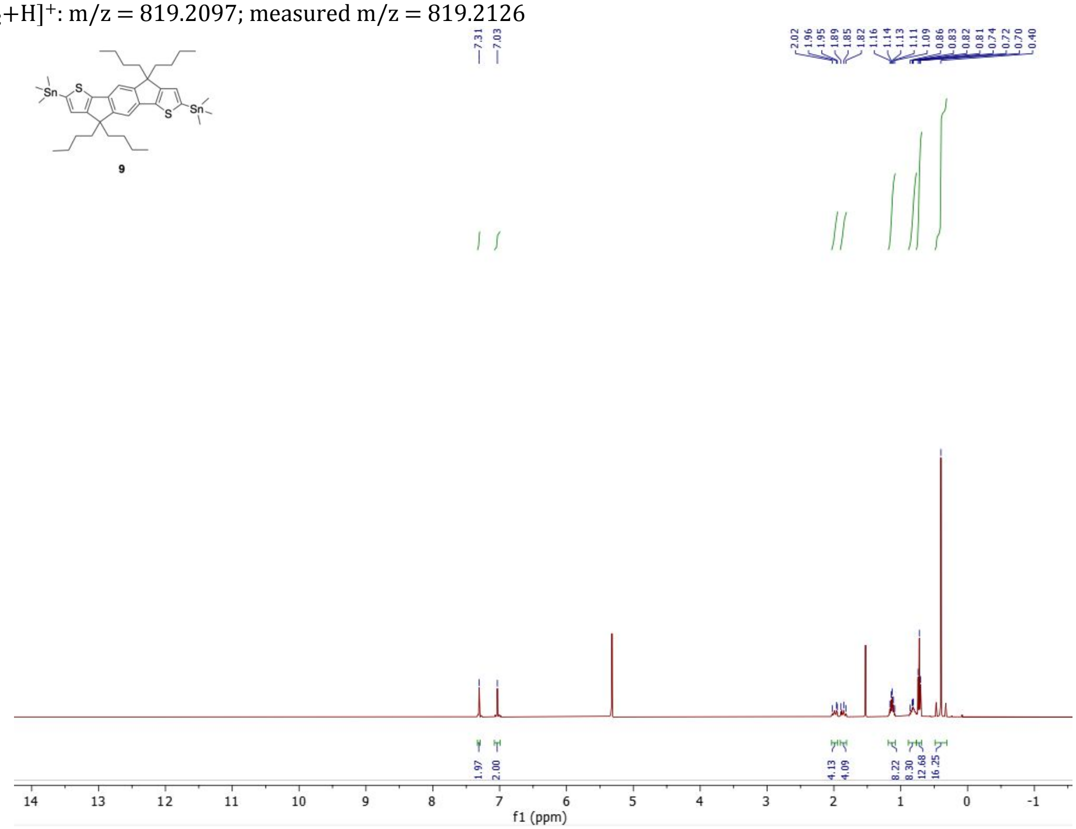

Figure S32. ^1H NMR of **9**, 400 MHz, CD_2Cl_2

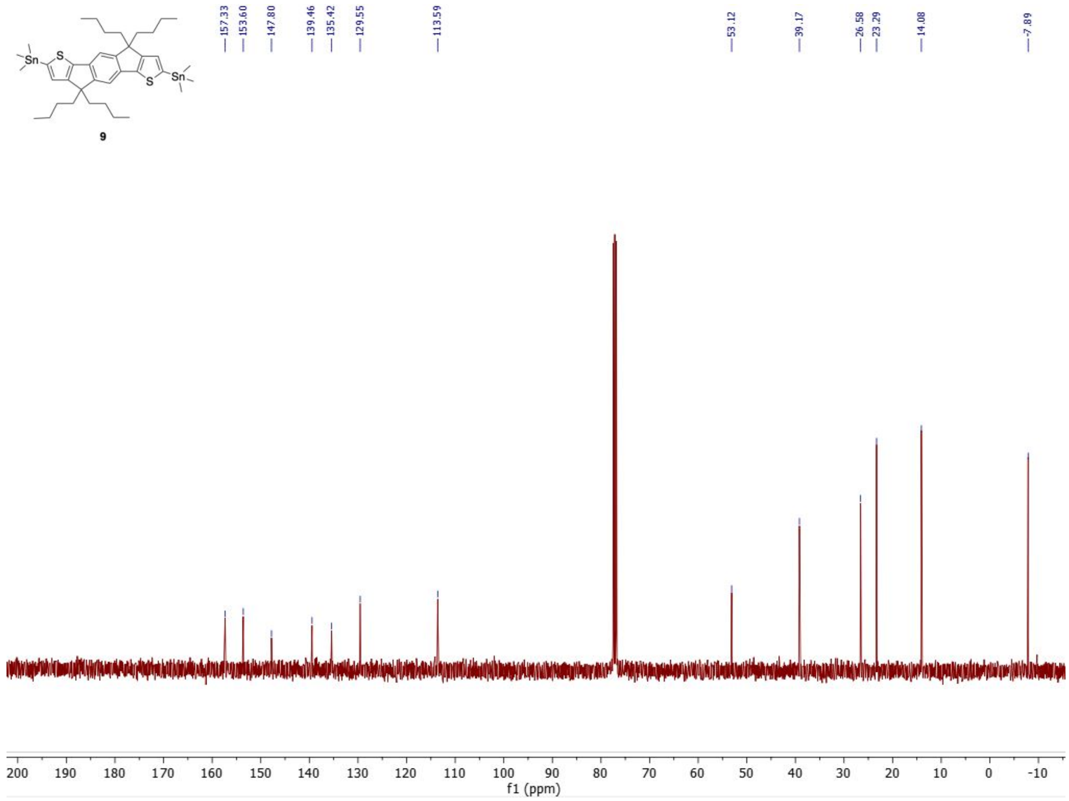

Figure S33. ^{13}C NMR of **9**, 400 MHz, $CDCl_3$

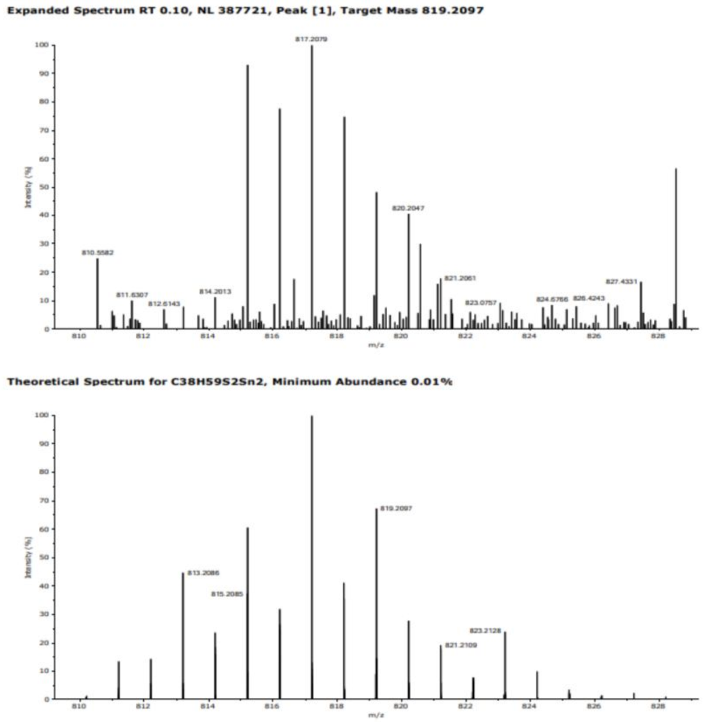

Figure S34. HRMS (ESI) of **9**

10

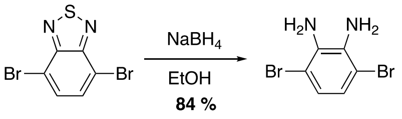

Scheme S11. Synthesis of **10**

The synthesis of **10** was adapted from a previously reported procedure.[8] 4,7-Dibromo-2,1,3-benzothiadiazole (20.0 g, 68.0 mmol, 1 eq) and ethanol (500 mL) were cooled to 0 °C. $NaBH_4$ (25.7 g, 680.4 mmol, 10 eq) was added in portions under stirring and it was allowed to reach room temperature overnight. The ethanol was removed under reduced pressure, ethyl acetate was added and everything was transferred to a separating funnel along with water. The organic fraction was extracted and washed with water, brine and Na_2SO_4 was added. This underwent gravity filtration, and the solvent was removed. This was purified on a silica column, eluting with petroleum ether: (40:60). After removing the solvent from the fractions containing product, a white solid **10** was recovered (15.4 g, 84%). ^1H NMR (400 MHz, $(CD_3)_2SO$) δ 6.64 (s, 2H), 5.01 (s, 4H).^{13}C NMR (400 MHz, $(CD_3)_2SO$) δ 133.2, 120.7, 106.6.

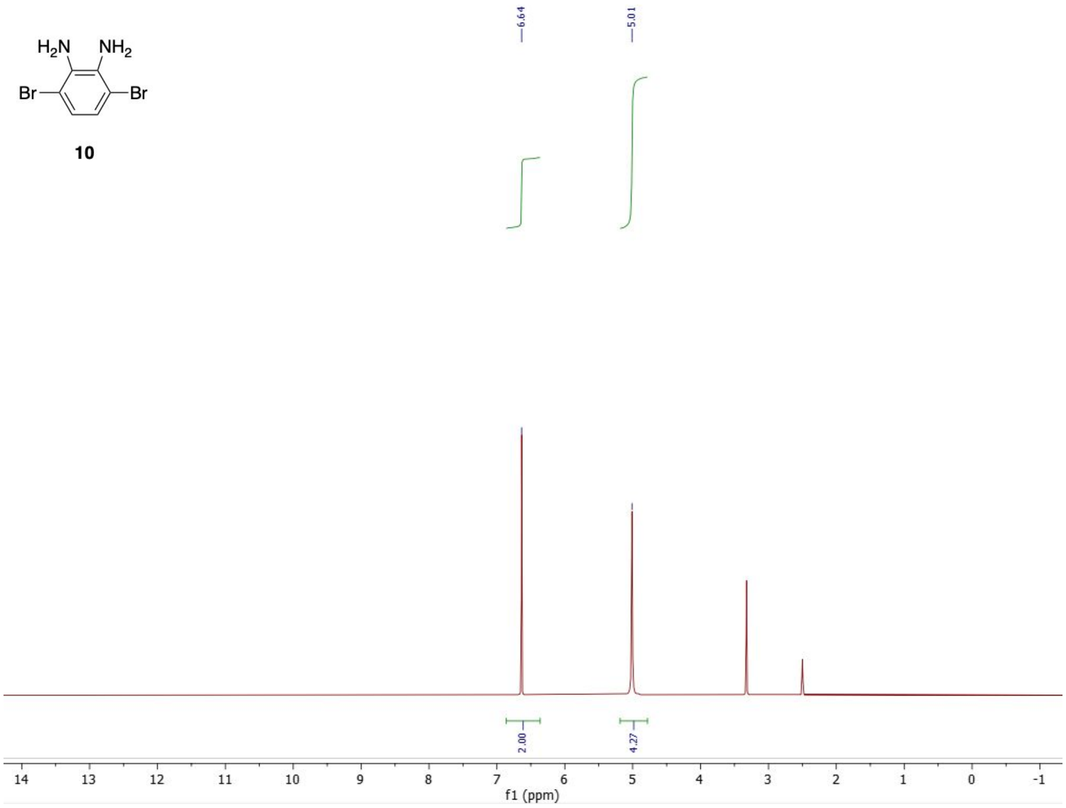

Figure S35. ^1H NMR of **10**, 400 MHz, $(CD_3)_2SO$

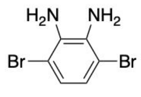

10

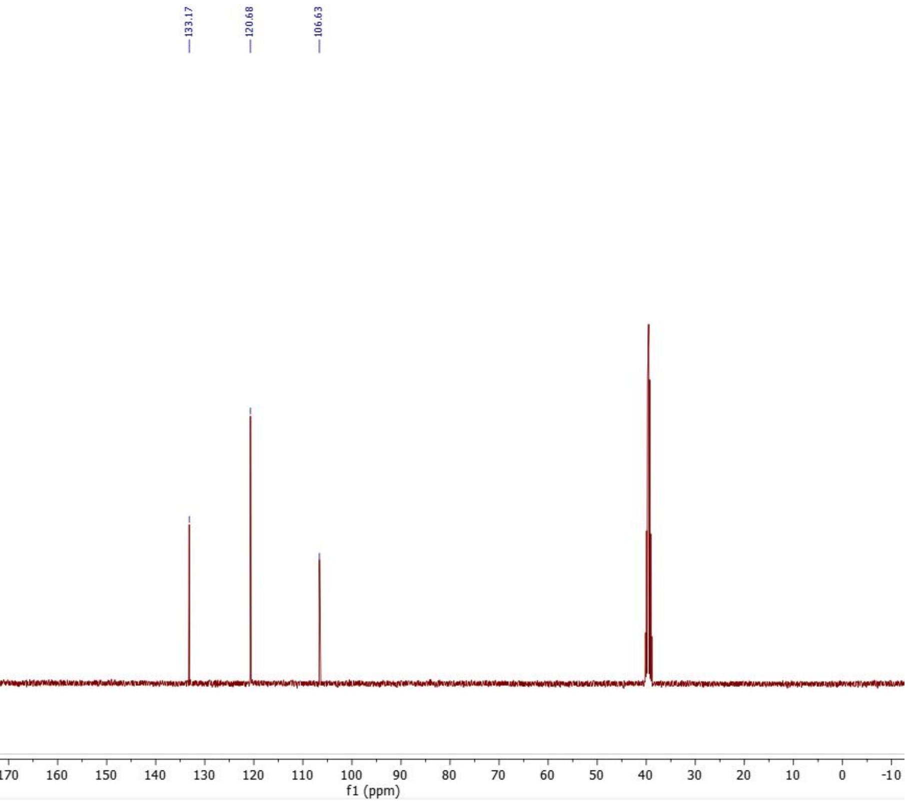

Figure S36. ^{13}C NMR of **10**, 400 MHz, $(CD_3)_2SO$

11

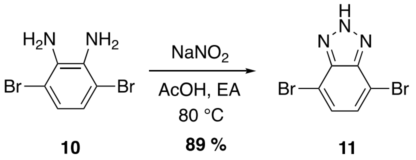

Scheme S12. Synthesis of **11**

The synthesis of **11** was adapted from a previously reported procedure.[9] **10** (3.69 g, 18.9 mmol, 1 eq) was dissolved in acetic acid (50 mL) and ethyl acetate (20 mL). $NaNO_2$ (1.15 g, 16.7 mmol, 1.2 eq) was added in portions under stirring and it was refluxed for 4 h, by which time product had precipitated out somewhat. This was poured into ice water, neutralised with NaOH solution then transferred to a separating funnel. Ethyl acetate was added to extract the product, which was subsequently washed with water, brine and Na_2SO_4 was added. This underwent gravity filtration, and the solvent was removed to give a pale slightly pink solid **11** (3.41, 89%). ^{1}H NMR (400 MHz, $CDCl_3$) δ 12.14 (s, br, 1H) 7.51 (s, 2H). This was too insoluble for ^{13}C NMR.

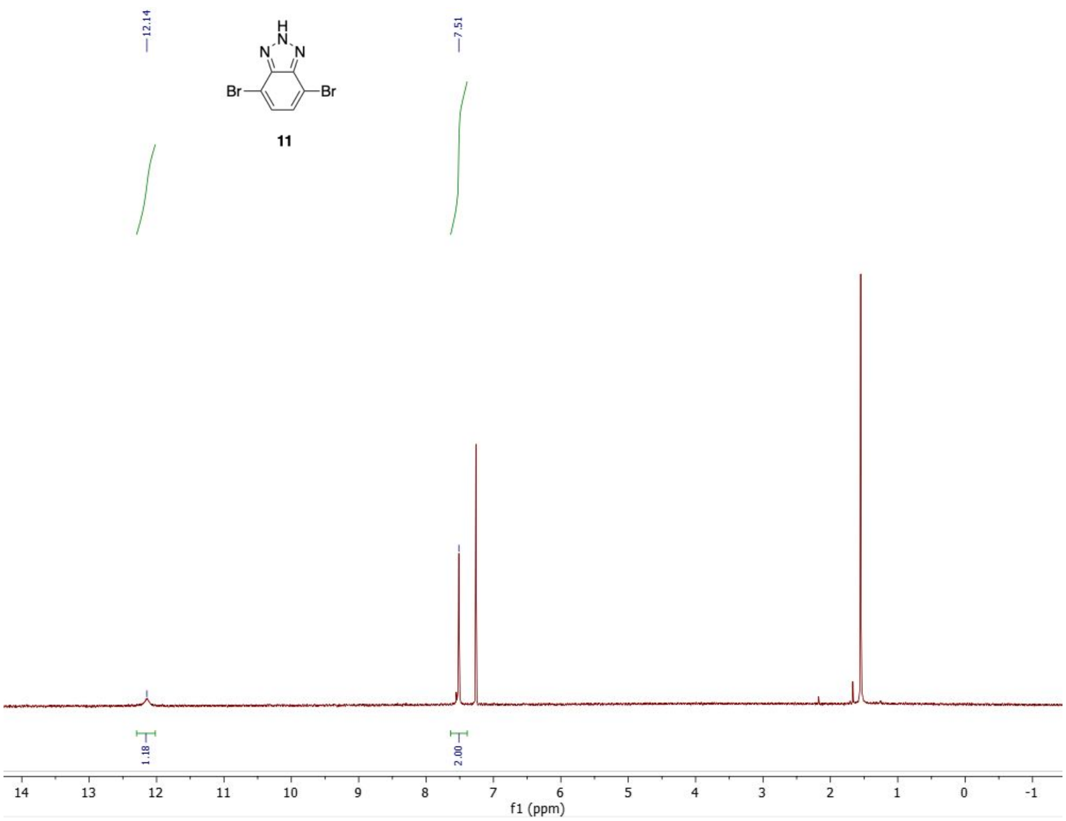

Figure S37. ^{1}H NMR of **11**, 400 MHz, $CDCl_3$

12

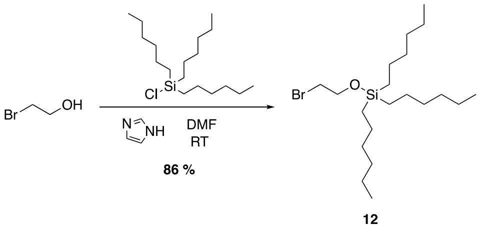

Scheme S13. Synthesis of **12**

2-bromoethanol (1.77 g, 14.2 mmol, 0.9 eq) trihexylsilyl chloride (5.03 g, 15.8 mmol, 1 eq) and imidazole (2.15g, 31.5 mmol, 2 eq) were purged with N_2 and dissolved in anhydrous dimethylformamide (100 mL). This was stirred for 4 h then poured into a separating funnel. The product was extracted with dichloromethane and washed twice with water, once with brine and Na_2SO_4 was added. This underwent gravity filtration, and the solvent was removed under reduced pressure to recover a colourless oil **12** which was used without further purification (4.98 g, 86%). ^{1}H NMR (400 MHz, $CDCl_3$) δ 3.86 (t, *J* = 6.7 Hz, 2H), 3.38 (t, *J* = 6.7 Hz, 2H), 1.32-1.26 (m, 24H), 0.90-0.87 (m, 9H), 0.62-0.58 (m, 6H). ^{13}C NMR (400 MHz, $CDCl_3$) δ 63.4, 33.4, 33.2, 31.7, 23.2, 22.8, 14.3, 13.7.

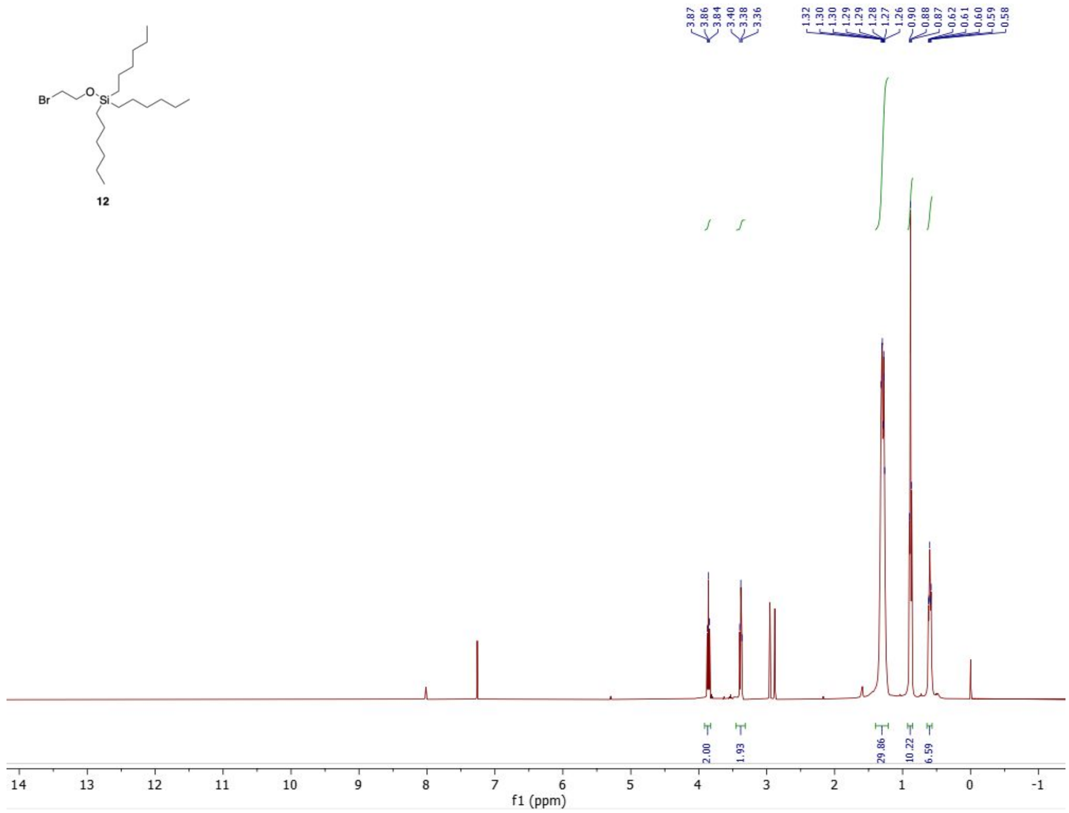

Figure S38. ^{1}H NMR of **12**, 400 MHz, $CDCl_3$

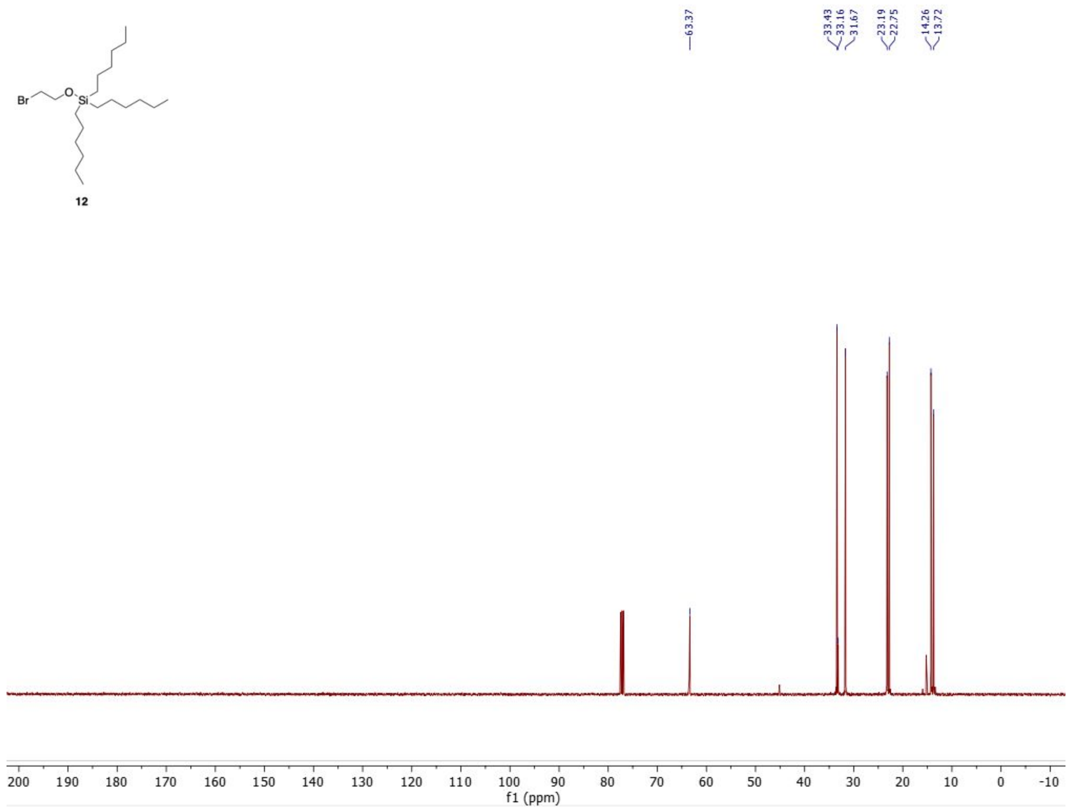

Figure S39. ^{13}C NMR of **12**, 400 MHz, $CDCl_3$

13

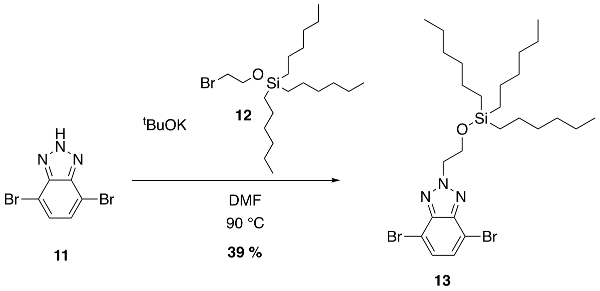

Scheme S14. Synthesis of **13**

11 (1.89 g, 6.8 mmol, 1 eq) and potassium tert-butoxide (0.92, 8.2 mmol, 1.2 eq) were added to a 2-neck round bottom flask and purged with N_2. Anhydrous dimethylformamide (50 mL) followed by **12** (3.34g, 8.2 mmol, 1.2 eq) and it was stirred at 90 °C for 4 h. After complete reaction it was poured into a separating funnel along with water. The product was extracted with dichloromethane and washed twice with water, once with brine and Na_2SO_4 was added. This underwent gravity filtration, and the solvent was removed under reduced pressure. The resultant oil was purified on a silica column, eluting with a graduated eluent, from pure petroleum ether to petroleum ether: dichloromethane (70:30). Removal of solvent from the product fractions gave a colourless viscous oil **13** (1.6 g, 39%). ^{1}H NMR (400 MHz, $CDCl_3$) δ 7.44 (s, 2H), 4.88 (t, $J = 5.6$ Hz, 2H), 4.28 (t, $J = 5.6$ Hz, 2H), 1.30 – 1.06 (m, 24H), 0.86 (t, $J = 7.0$ Hz, 9H), 0.54 – 0.36 (m, 6H). ^{13}C NMR (400 MHz, $CDCl_3$) δ 144.0, 129.70, 110.2, 61.6, 59.6, 33.4, 31.6, 23.0, 22.7, 14.3, 13.5. HSMS (ESI): HR-MS calculated for $[C_{26}H_{45}Br_2N_3OSi+H]^+$: m/z = 602.1771; measured m/z = 602.1784

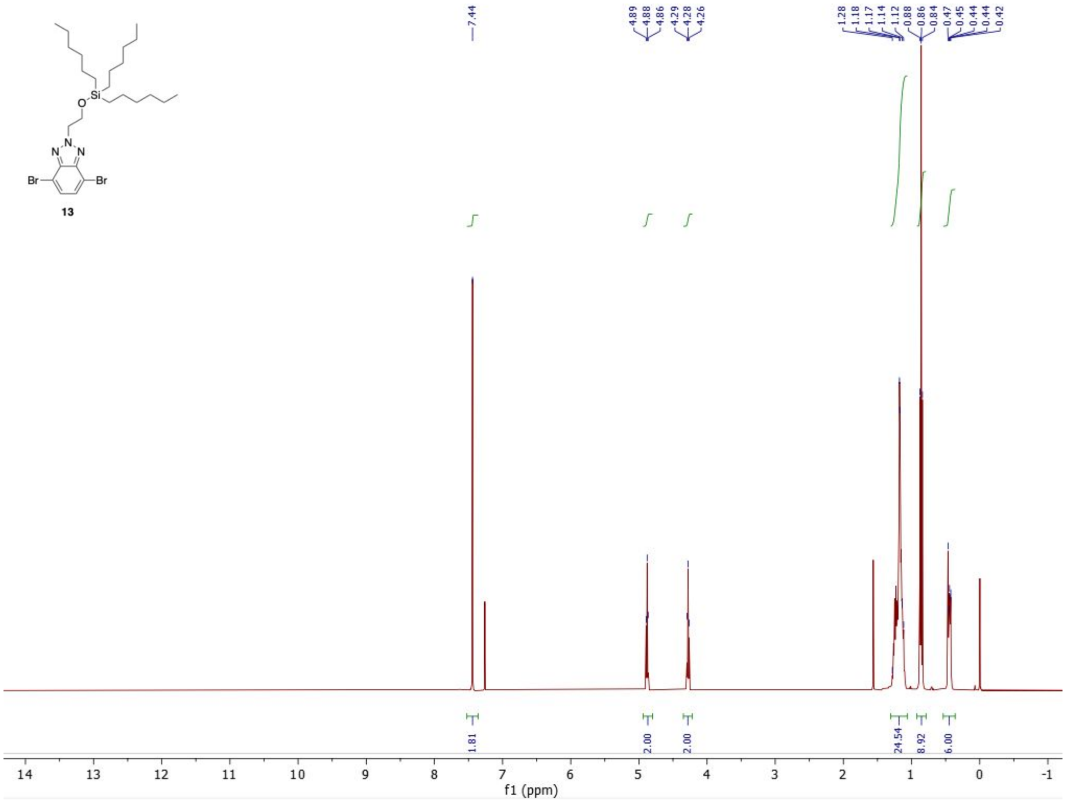

Figure S40. ^{1}H NMR of **13**, 400 MHz, $CDCl_3$

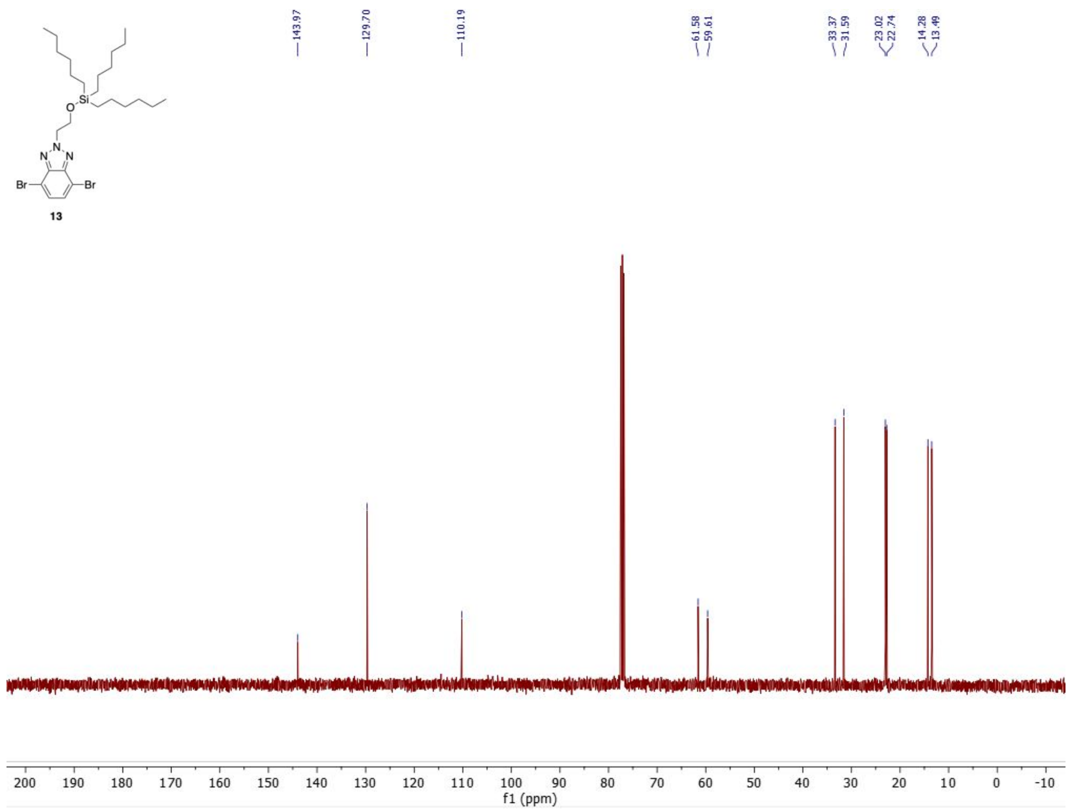

Figure S41. ^{13}C NMR of **13**, 400 MHz, $CDCl_3$

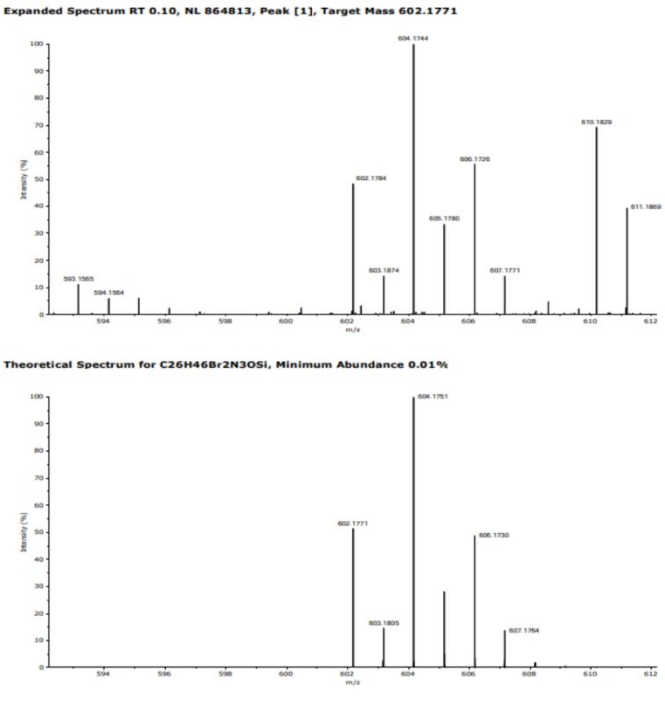

Figure S42. HSMS (ESI) of **13**

14

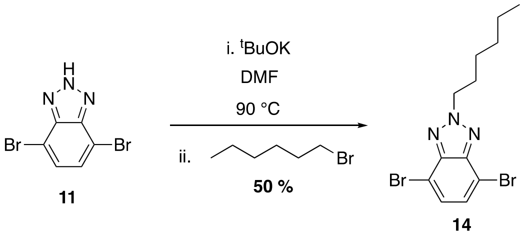

Scheme S15. Synthesis of **14**

11 (500 mg, 1.81 mmol, 1 eq), and potassium tert-butoxide (243 mg, 2.17 mmol, 1.2 eq) were added to a microwave vial and purged with N_2. Anhydrous dimethylformamide (10 mL) followed by 1-bromohexane (0.3 mL, 2.17 mmol, 1.2 eq) and it was stirred at 90 °C for 4 h. After complete reaction it was poured into a separating funnel along with water. The product was extracted with dichloromethane and washed twice with water, once with brine and Na_2SO_4 was added. This underwent gravity filtration, and the solvent was removed under reduced pressure. The resultant oil was purified on a silica column, eluting with a graduated eluent, from petroleum ether: dichloromethane (90: 10) to petroleum ether: dichloromethane (80:20). Removal of solvent from the product fractions gave a colourless oil **14** (328 mg g, 50%). ^1H NMR (400 MHz, $CDCl_3$) δ 7.44 (s, 2H), 4.77 (t, J = 7.5 Hz, 2H), 2.18 – 2.10 (m, 2H), 1.40 – 1.28 (m, 6H), 0.88 (t, J = 6.9 Hz, 3H). ^{13}C NMR (400 MHz, $CDCl_3$) δ 143.8, 129.6, 110.1, 57.9, 31.3, 30.3, 26.3, 22.5, 14.1. HSMS (ESI): HR-MS calculated for $[C_{12}H_{15}Br_2N_3+H]^+$: m/z = 359.9705; measured m/z = 359.9716

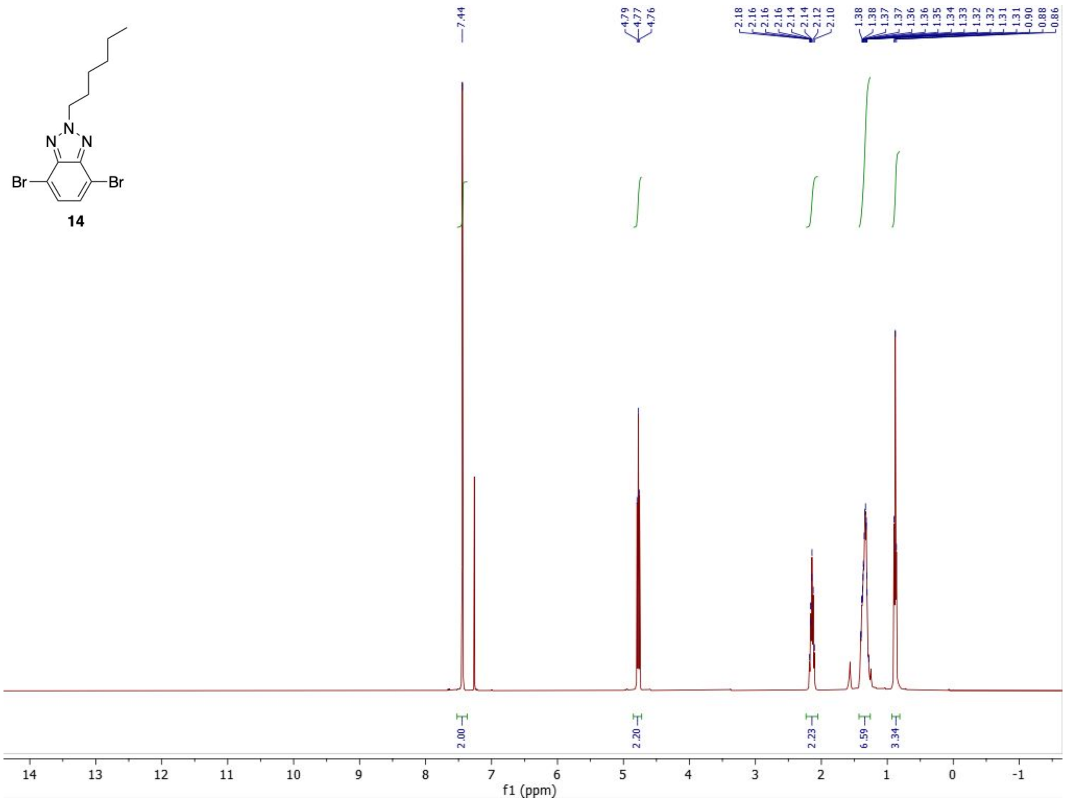

Figure S43. ^1H NMR of **14**, 400 MHz, $CDCl_3$

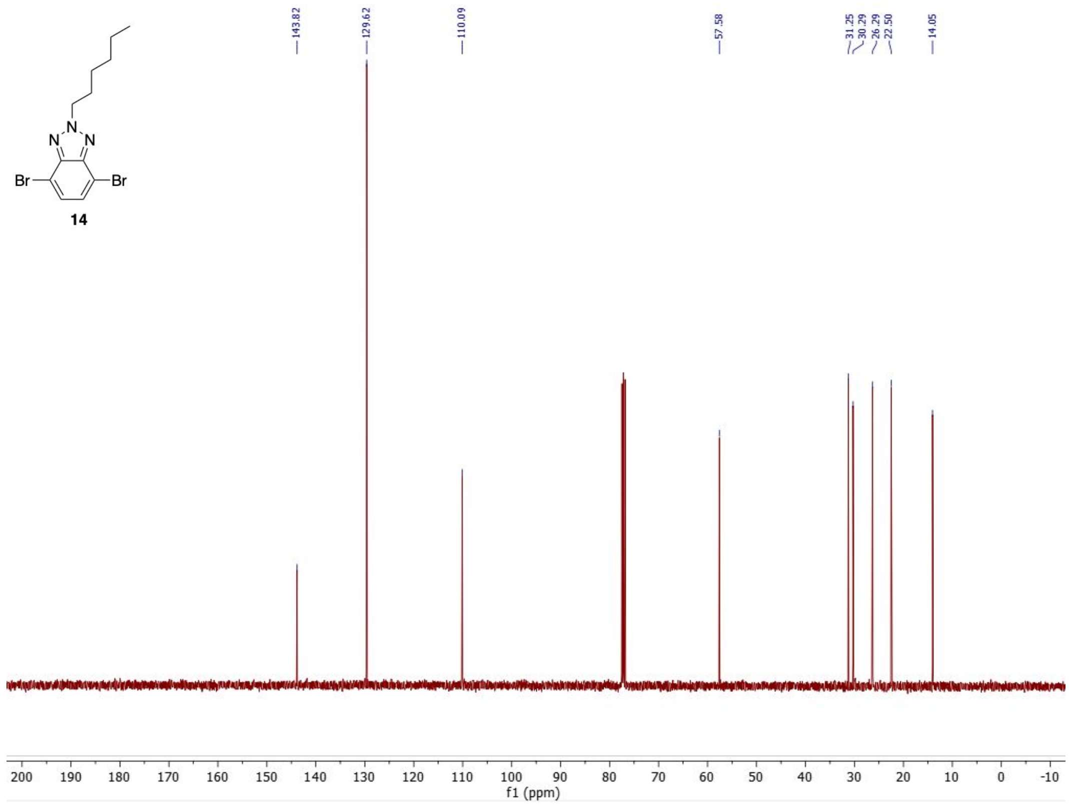

Figure S44. ^{13}C NMR of **14**, 400 MHz, $CDCl_3$

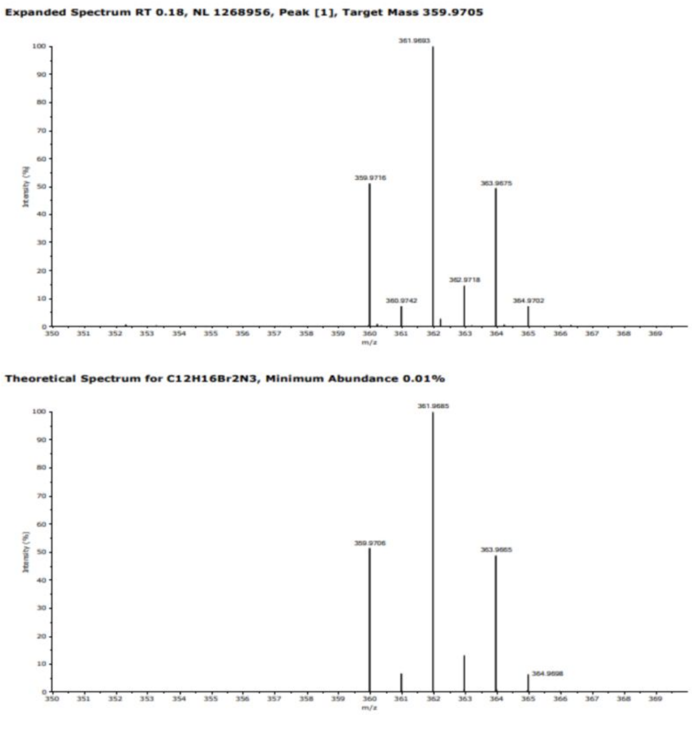

Figure S45. HSMS (ESI) of **14**

Sp-Si

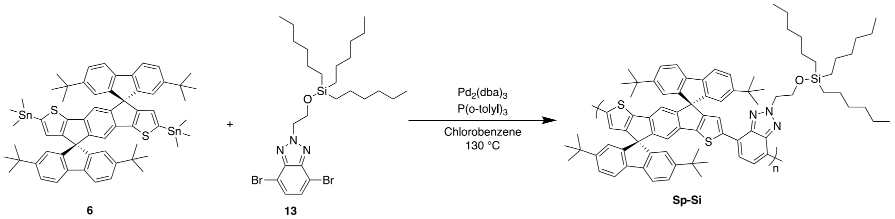

Scheme S14. Synthesis of **Sp-Si**

6 (241.34 mg, 216 µmol, 1 eq), **13** (130.43 mg, 216µmol, 1 eq), tri(o-tolyl)phosphine (5.3 mg, 17 µmol, 0.08 eq) and tris(dibenzylideneacetone) dipalladium(0) (4.0 mg, 4 µmol, 0.02 eq) were added to a microwave vial, it was capped then degassed with N_2 for 30 minutes. Anhydrous chlorobenzene (4.3 mL) was added and it was heated to 130 °C overnight. This was cooled, precipitated into methanol and poured into a cellulose Soxhlet thimble. This underwent a Soxhlet extraction washing with acetone followed by hexane, ethyl acetate then extracted with chlorobenzene. This was concentrated under reduced pressure, then precipitated into methanol and collected by vacuum filtration to give a dark purple solid **Sp-Si** (225 mg, 84%). ^{1}H NMR (500 MHz, $CDCl_3$) δ 7.75 (br,, d, $J = 8.0$ Hz, 4H), 7.45-7.31 (br, m, 6H), 7.17 (br, 2H), 6.84-6.70 (br, m,6H), 4.72 (br, 2H), 4.13 (br, 2H), 1.19-0.98, (br, m, 60H), 0.73 (t, $J = 7.2$ Hz, 9H), 0.31 (br, 6H). GPC (chlorobenzene) M_N 24107 gmol^{-1}, M_W 61073 gmol^{-1}, PD 2.53

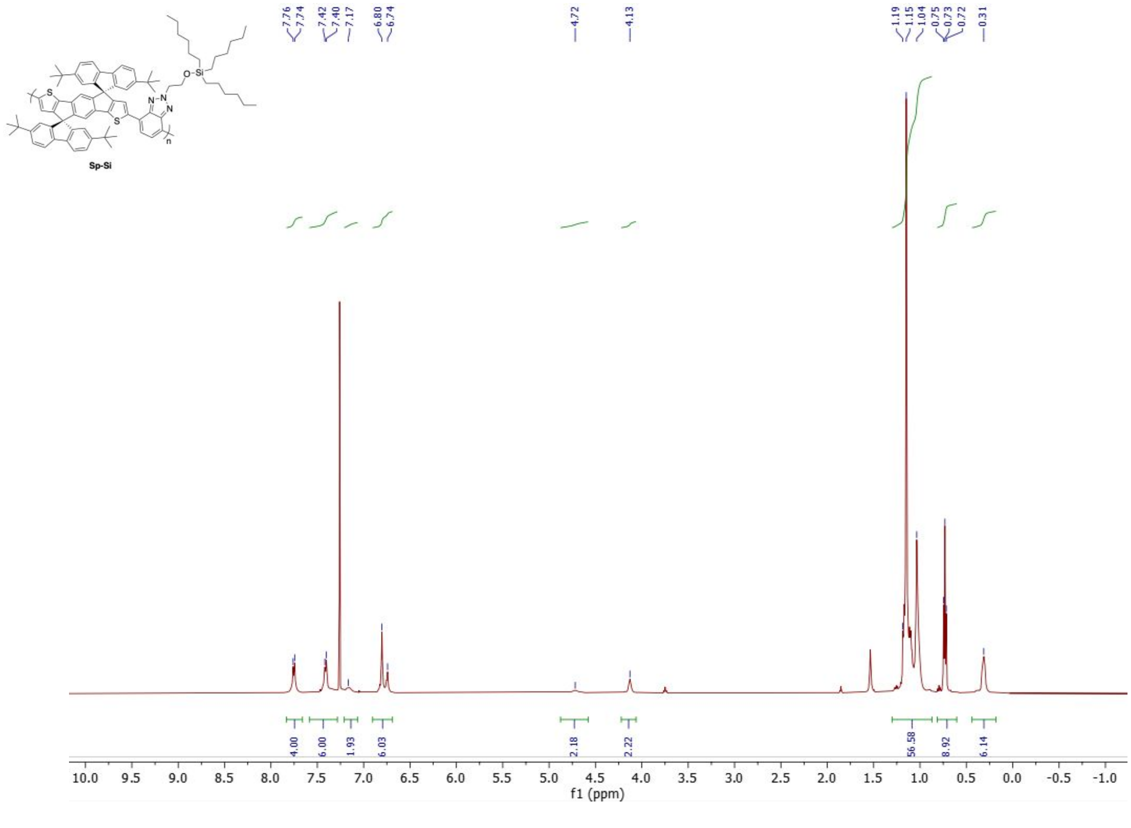

Figure S46. ^{1}H NMR of **Sp-Si**, 500 MHz, $CDCl_3$

Sp-Hex

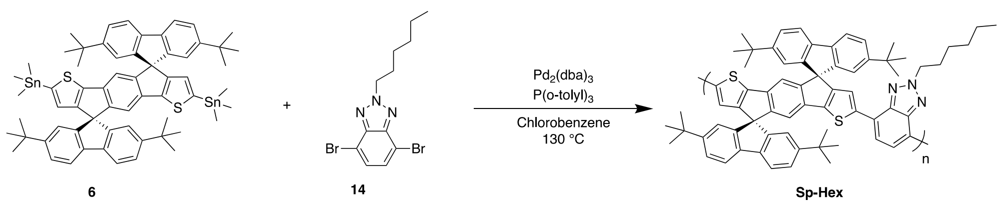

Scheme S15. Synthesis of **Sp-Hex**

6 (129.87 mg, 116 μmol, 1 eq), **14** (41.99 mg, 116 μmol, 1 eq), tri(o-tolyl)phosphine (2.8 mg, 9 μmol, 0.08 eq) and tris(dibenzylideneacetone) dipalladium(0) (2.1 mg, 2 μmol, 0.02 eq) were added to a microwave vial, it was capped then degassed with N_2 for 30 minutes. Anhydrous chlorobenzene (2.7 mL) was added and it was heated to 130 °C overnight. This was cooled, precipitated into methanol and collected by vacuum filtration. In the filter it was washed with hot acetone, hot hexane, hot ethyl acetate followed by more methanol, then collected to give a dark purple solid **Sp-Hex** (107 mg, 93%). Due to low solubility NMR and GPC could not be performed.

Bu-Si

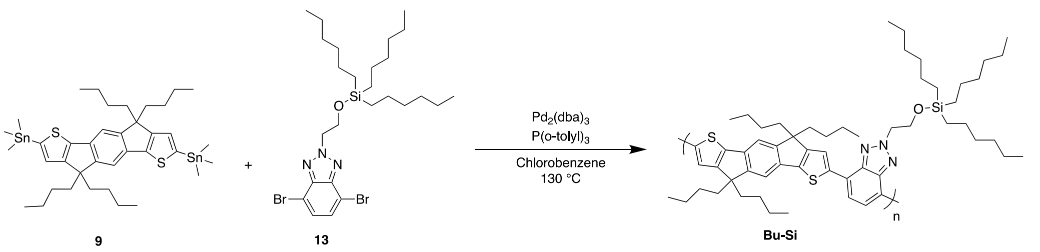

Scheme S16. Synthesis of **Bu-Si**

9 (165.43 mg, 203 µmol, 1 eq), **13** (122.30 mg, 203 µmol, 1 eq), tri(o-tolyl)phosphine (4.9 mg, 16 µmol, 0.08 eq) and tris(dibenzylideneacetone) dipalladium(0) (3.7 mg, 4 µmol, 0.02 eq) were added to a microwave vial, it was capped then degassed with N_2 for 30 minutes. Anhydrous chlorobenzene (4.0 mL) was added and it was heated to 130 °C overnight. This was cooled, precipitated into methanol and poured into a cellulose Soxhlet thimble. This underwent a Soxhlet extraction washing with methanol followed by hexane then extracted with chloroform. The chloroform fraction was concentrated under reduced pressure then precipitated into methanol and collected by vacuum filtration to give a dark purple solid **Bu-Si** (121 mg, 64%). ^{1}H NMR (500 MHz, $CDCl_3$) δ 7.99 (br, 2H), 7.72 (br, 2H), 7.38 (br, 2H), 5.03 (br, 2H), 4.46 (br, 2H), 2.17-1.92 (b, m, 8H), 1.33-0.71 (br, m, 69H), 0.54 (br, 6H). GPC (chlorobenzene) M_N 27027 gmol^{-1}, MW 45072 gmol^{-1}, PD 1.66

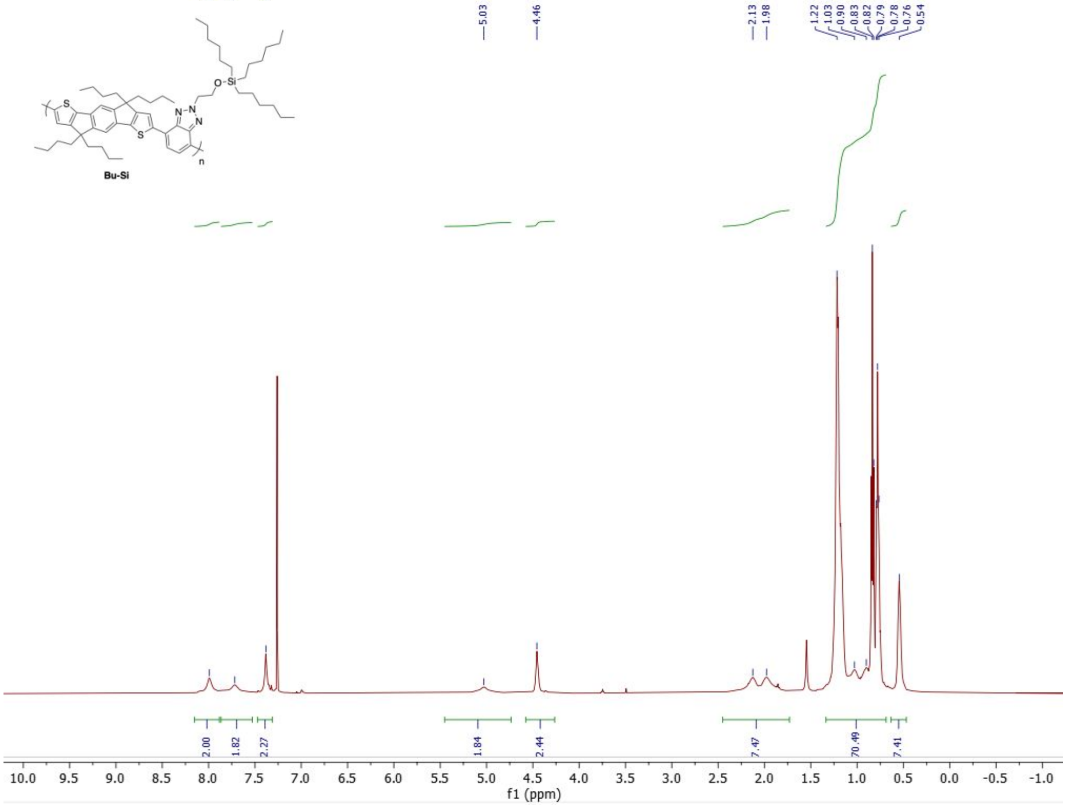

Figure S47. ^{1}H NMR of **Bu-Si**, 500 MHz, $CDCl_3$

Supporting References:

(1) Trasatti, S. The Absolute Electrode Potential: An Explanatory Note (Recommendations 1986). *Pure and Applied Chemistry* **1986**, *58* (7), 955–966. https://doi.org/10.1351/PAC198658070955.

(2) Kosco, J.; Bidwell, M.; Cha, H.; Martin, T.; Howells, C. T.; Sachs, M.; Anjum, D. H.; Gonzalez Lopez, S.; Zou, L.; Wadsworth, A.; Zhang, W.; Zhang, L.; Tellam, J.; Sougrat, R.; Laquai, F.; DeLongchamp, D. M.; Durrant, J. R.; McCulloch, I. Enhanced Photocatalytic Hydrogen Evolution from Organic Semiconductor Heterojunction Nanoparticles. *Nat Mater* **2020**, *19* (5), 559–565. https://doi.org/10.1038/s41563-019-0591-1.

(3) Wang, X.; Chen, L.; Chong, S. Y.; Little, M. A.; Wu, Y.; Zhu, W. H.; Clowes, R.; Yan, Y.; Zwijnenburg, M. A.; Sprick, R. S.; Cooper, A. I. Sulfone-Containing Covalent Organic Frameworks for Photocatalytic Hydrogen Evolution from Water. *Nat Chem* **2018**, *10* (12), 1180–1189. https://doi.org/10.1038/s41557-018-0141-5.

(4) Chen, C. T.; Chao, W. S.; Liu, H. W.; Wei, Y.; Jou, J. H.; Kumar, S. Spirally Configured Cis-Stilbene/Fluorene Hybrids as Ambipolar, Fluorescent Materials for Organic Light Emitting Diode Applications. *RSC Adv* **2013**, *3* (24), 9381–9390. https://doi.org/10.1039/C3RA41642A.

(5) Zhang, W.; Smith, J.; Watkins, S. E.; Gysel, R.; McGehee, M.; Salleo, A.; Kirkpatrick, J.; Ashraf, S.; Anthopoulos, T.; Heeney, M.; McCulloch, I. Indacenodithiophene Semiconducting Polymers for High-Performance, Air-Stable Transistors. *J Am Chem Soc* **2010**, *132* (33), 11437–11439. https://doi.org/10.1021/JA1049324.

(6) Xiao, H.; Yin, H.; Wang, L.; Mei, C.; Zhang, X. Synthesis of 2,2'-Diamino-7-Tert-Butyl-9,9'-Spirobifluorene Starting from 4,4'-Di-Tert-Butylbiphenyl. *Monatsh Chem* **2012**, *143* (4), 683–686. https://doi.org/10.1007/S00706-011-0620-5.

(7) Peltier, J. D.; Heinrich, B.; Donnio, B.; Jeannin, O.; Rault-Berthelot, J.; Poriel, C. Modulating the Physical and Electronic Properties over Positional Isomerism: The Dispirofluorene–Dihydroindacenodithiophene (DSF-IDT) Family. *Chemistry – A European Journal* **2017**, *23* (68), 17290–17303. https://doi.org/10.1002/CHEM.201703320.

(8) Wang, L. K.; Zhou, J. J.; Lan, Y. B.; Ding, S. Y.; Yu, W.; Wang, W. Divergent Synthesis of Chiral Covalent Organic Frameworks. *Angewandte Chemie International Edition* **2019**, *58* (28), 9443–9447. https://doi.org/10.1002/ANIE.201903534.

(9) Fujiki, S.; Amaike, K.; Yagi, A.; Itami, K. Synthesis, Properties, and Material Hybridization of Bare Aromatic Polymers Enabled by Dendrimer Support. *Nature Communications 2022 13:1* **2022**, *13* (1), 1–9. https://doi.org/10.1038/s41467-022-33100-7.
